# Supplementary material for: Tunable electrocaloric effect in lead scandium tantalate through calcium doping
Source: Nat Commun. 2026 Apr 15;17:5250. doi: 10.1038/s41467-026-71911-0 (PMC13260448; doi:10.1038/s41467-026-71911-0)
Supplement: Supplementary file 1 — Supplementary Information [file 41467_2026_71911_MOESM1_ESM.pdf]

Supplementary Information: Tunable Electrocaloric Effect in Lead Scandium Tantalate through Calcium Doping

Youri Nouchokgwe*[1,2], Natalya S. Fedorova[1,2], Veronika Kovacova[1,2], Pranab Biswas[2,3], Ivana Gorican[4], Nejc Suban[4], Silvo Drnovsek[4], Matej Sadl[4], Michele Melchiorre[3], Binayak Mukherjee[1,2], Uros Prah[1,2], Guillaume F. Nataf[5], Torsten Granzow[1,2], Mael Guennou[2,3], Hana Ursic[4], Jorge Iñiguez-González[1,2,3], and Emmanuel Defay*[1,2,3]

[1] *Smart Materials Research Unit, Luxembourg Institute of Science and Technology, Maison des Matériaux, 28 Av. des Hauts Fourneaux, L-4362 Esch-Belval, Esch-sur-Alzette.*

[2] *Inter-institutional Research Group University of Luxembourg - LIST on Ferroic Materials,41 rue du Brill, L-4422 Belvaux, Luxembourg*

[3] *Department of Physics and Materials Science, University of Luxembourg, L-4422 Belvaux, Luxembourg*

[4] *Jožef Stefan Institute, Jamova cesta 39, Ljubljana 1000, Slovenia*

[5] *GREMAN UMR7347, CNRS, University of Tours, INSA Centre Val de Loire, 37000 Tours, France*

[*] *corresponding authors: youri.nouchokgwe@list.lu ; emmanuel.defay@list.lu*

March 27, 2026

Contents

Supplementary Note 1: Grain size of pure and Ca-doped PST 3

Supplementary Note 2: X-ray diffraction of pure and Ca-doped PST 5

Supplementary Note 3: Polarization versus electric field loops of pure and Ca-doped PST 6

Supplementary Note 4: Dielectric measurements of pure and Ca-doped PST 9

Supplementary Note 5: Thermal properties of pure and Ca-doped PST 12

Supplementary Note 6: Electrocaloric effect in PCa2ST 14

Supplementary Note 7: Electrocaloric effect in PCa4.6ST 20

Supplementary Note 8: Maximum electrocaloric effect in pure and Ca-doped PST 22

Supplementary Note 9: Raman spectra of pure and Ca-doped PST 23

Supplementary Note 10: Piezoresponse force microscopy 29

Supplementary Note 11: Density Functional Theory Calculations 33

References 46

Supplementary Note 1: Grain size of pure and Ca-doped PST

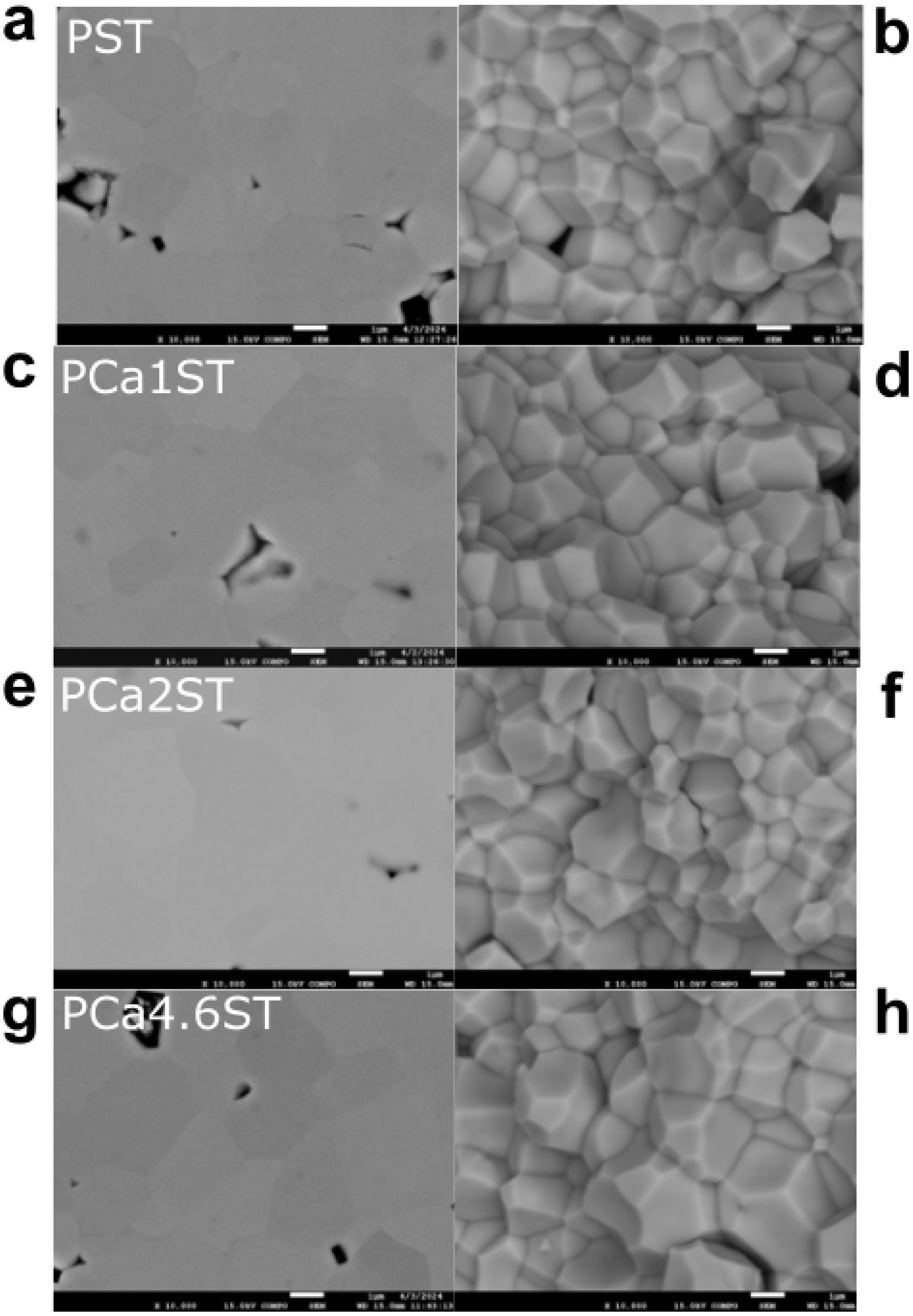

Supplementary Figure 1: **Scanning electron microscope (SEM) images of pure and Ca-doped PST.** The SEM images of PST (a, b), PCa1ST (c,d), PCa2ST(e,f), and PCa4.6ST(g,h). The length of the scale bar is 1 μm.

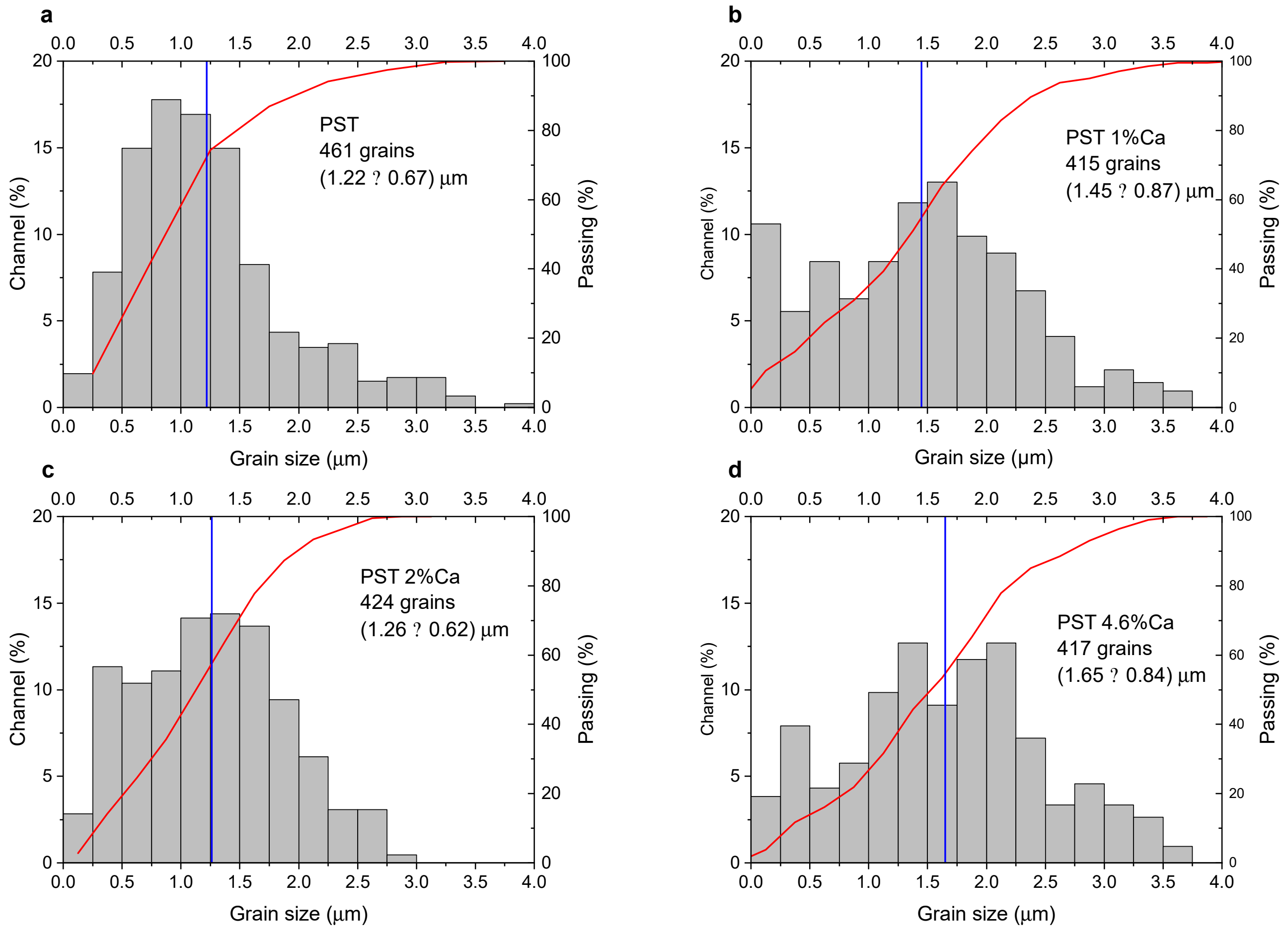

Supplementary Figure 2: **Grain size of pure and Ca-doped PST.** (a), (b), (c), and (d) are respectively the grain size calculated of PST, PCa1ST, PCa2ST, and PCa4.6ST. It was determined by analyzing digitalized microstructure images in Image Tool software (version 3.0, United States).

Supplementary Note 2: X-ray diffraction of pure and Ca-doped PST

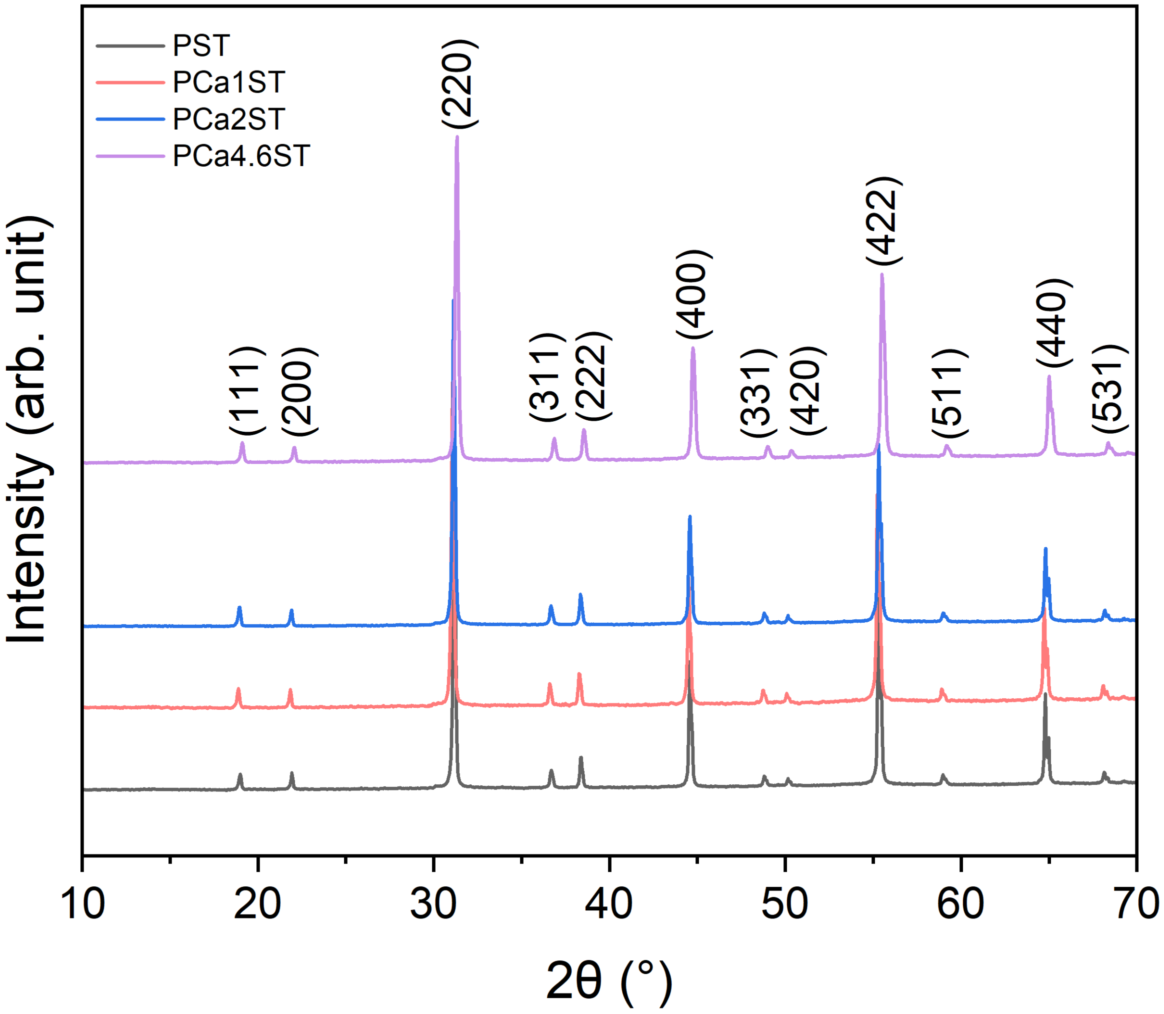

Supplementary Figure 3: **X-ray diffraction (XRD) of Ca-doped PST.** The XRD measurements were carried out at 50 ℃ (in the cubic phase).

Supplementary Note 3: Polarization versus electric field loops of pure and Ca-doped PST

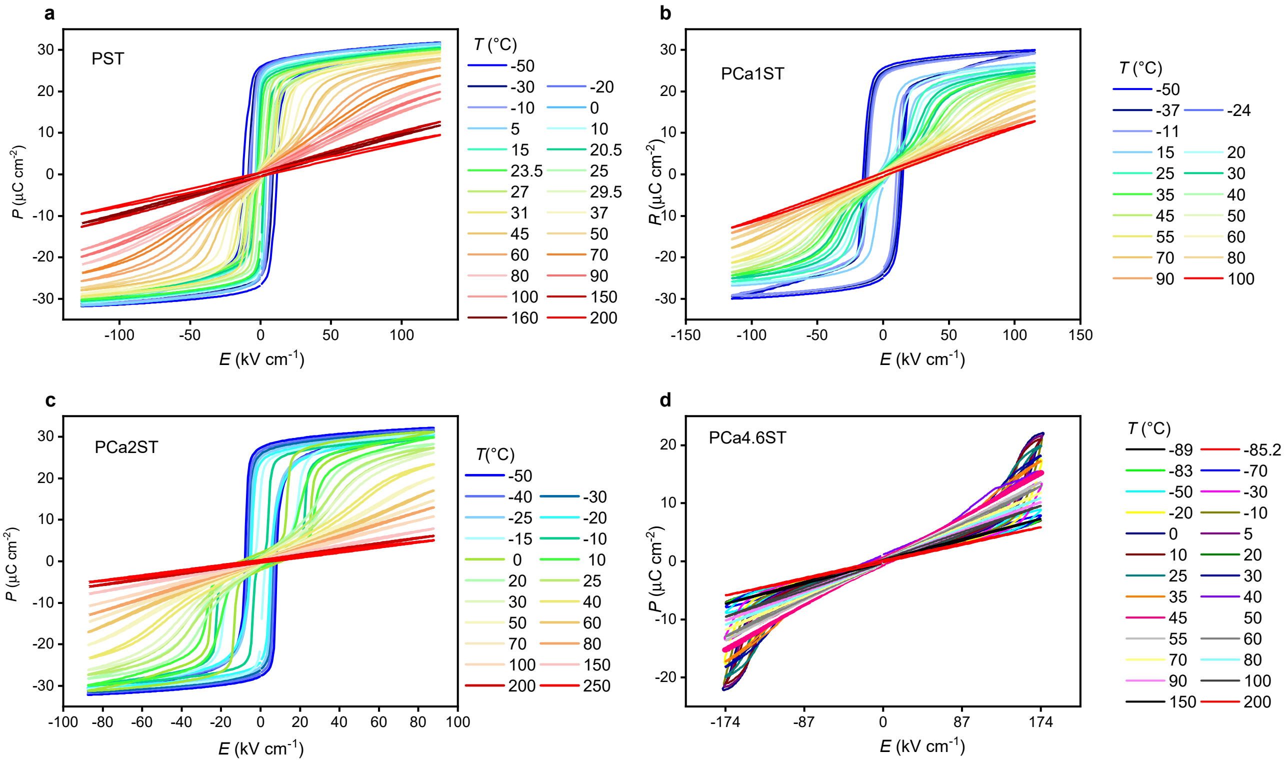

Supplementary Figure 4: **Polarization-electric field (PE) loops of pure and Ca-doped PST** a) pure PST, b) PCa1ST, c) PCa2ST, d) PCa4.6ST.

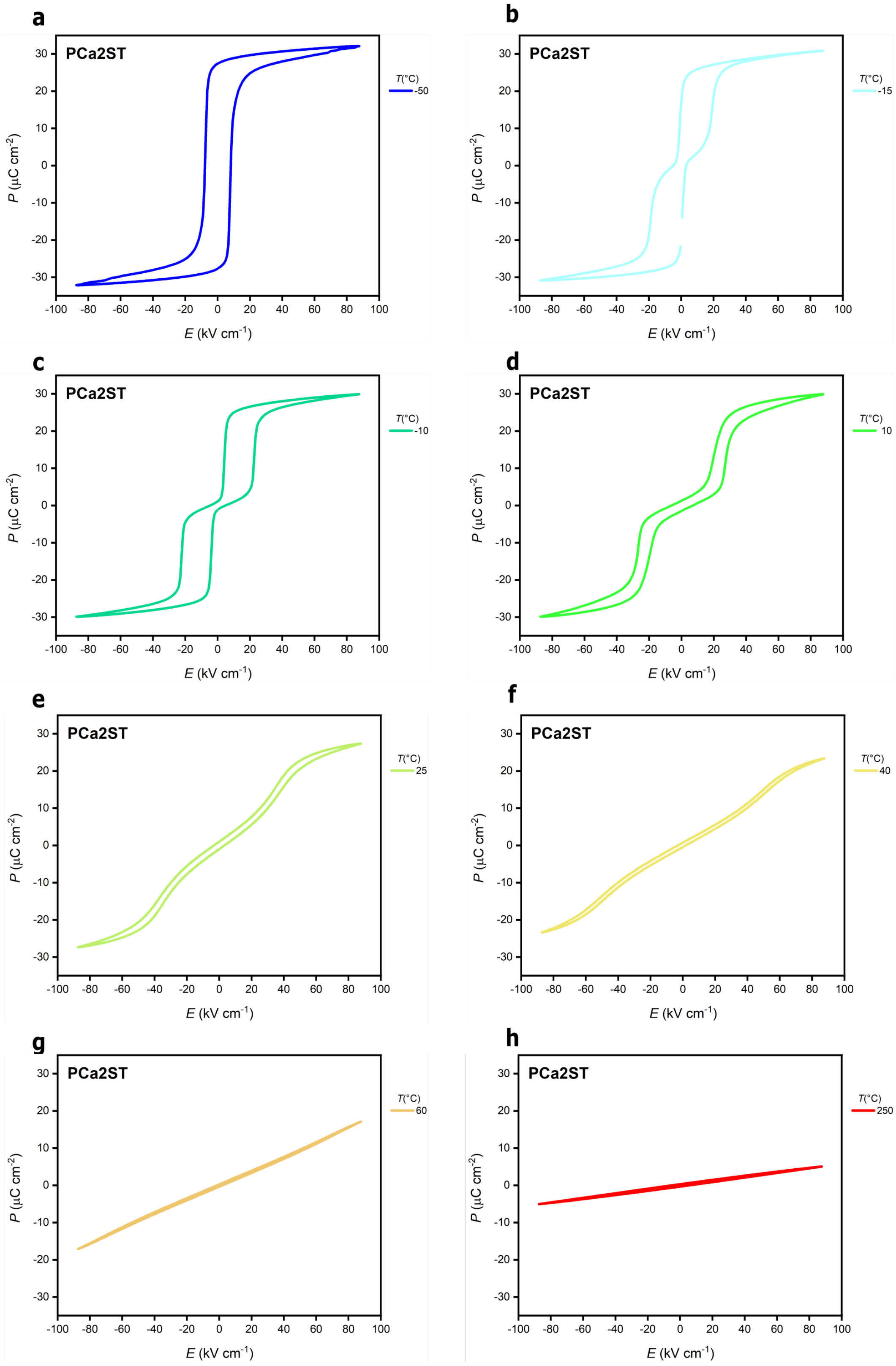

Supplementary Figure 5: **Temperature dependence of polarization-electric field loops of 2%Ca-doped PST.** a) -50℃, the material is FE; b) -15℃, the material transitions to an AFE phase; c) -10℃, the material is AFE; d) 10℃ and e) 25℃; the material transitions to a PE phase f) 40℃; g) 60℃; h) 250℃.

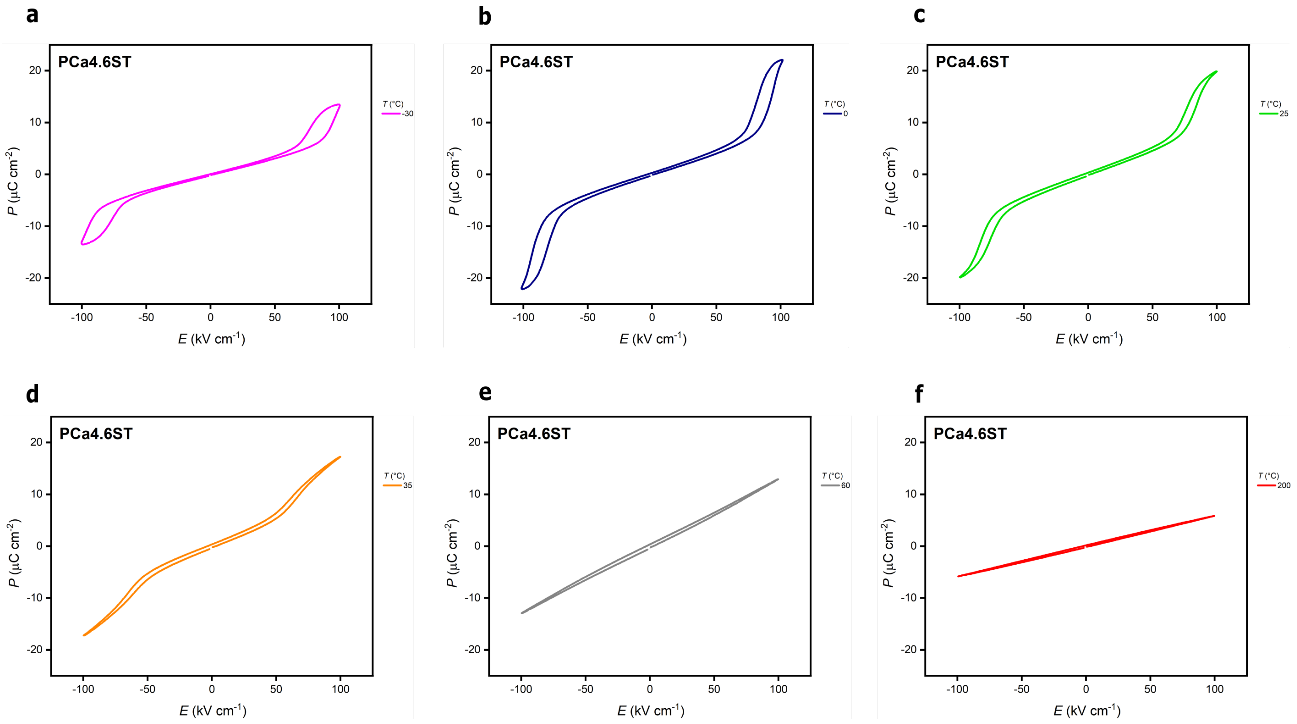

Supplementary Figure 6: **Temperature dependence of polarization-electric field loops of 4.6%Ca-doped PST.** a) -30℃, b) 0℃, c)25℃, d) 35℃, e) 60℃ and f) 200℃.

Supplementary Note 4: Dielectric measurements of pure and Ca-doped PST

In this Supplementary Note, we present dielectric measurements collected at 1 kHz for Ca-doped PST samples during both heating and cooling at a rate of 1 K/min. The measured transition temperatures are consistent with those obtained from DSC. More specifically, in PCa2ST we observe a shoulder around $-17\,^{\circ}\mathrm{C}$, which coincides with the field-induced FE-AFE transition identified by *in-situ* field DSC (Figures 1c and 3c). In addition, dielectric measurements of the antiferroelectric PCa4.6ST sample reveal an anomaly at approximately 15 ℃. Such anomalies are commonly observed in lead-based antiferroelectric materials [1–4] and are attributed to a transition from an incommensurate AFE phase to a commensurate AFE phase. Finally, the dielectric constant exhibits an increasingly diffuse transition with rising Ca content. This diffuse behaviour is attributed to calcium doping, which induces the coexistence of competing AFE and FE phases, a phenomenon reported in doped Pb-based perovskites [5, 6].

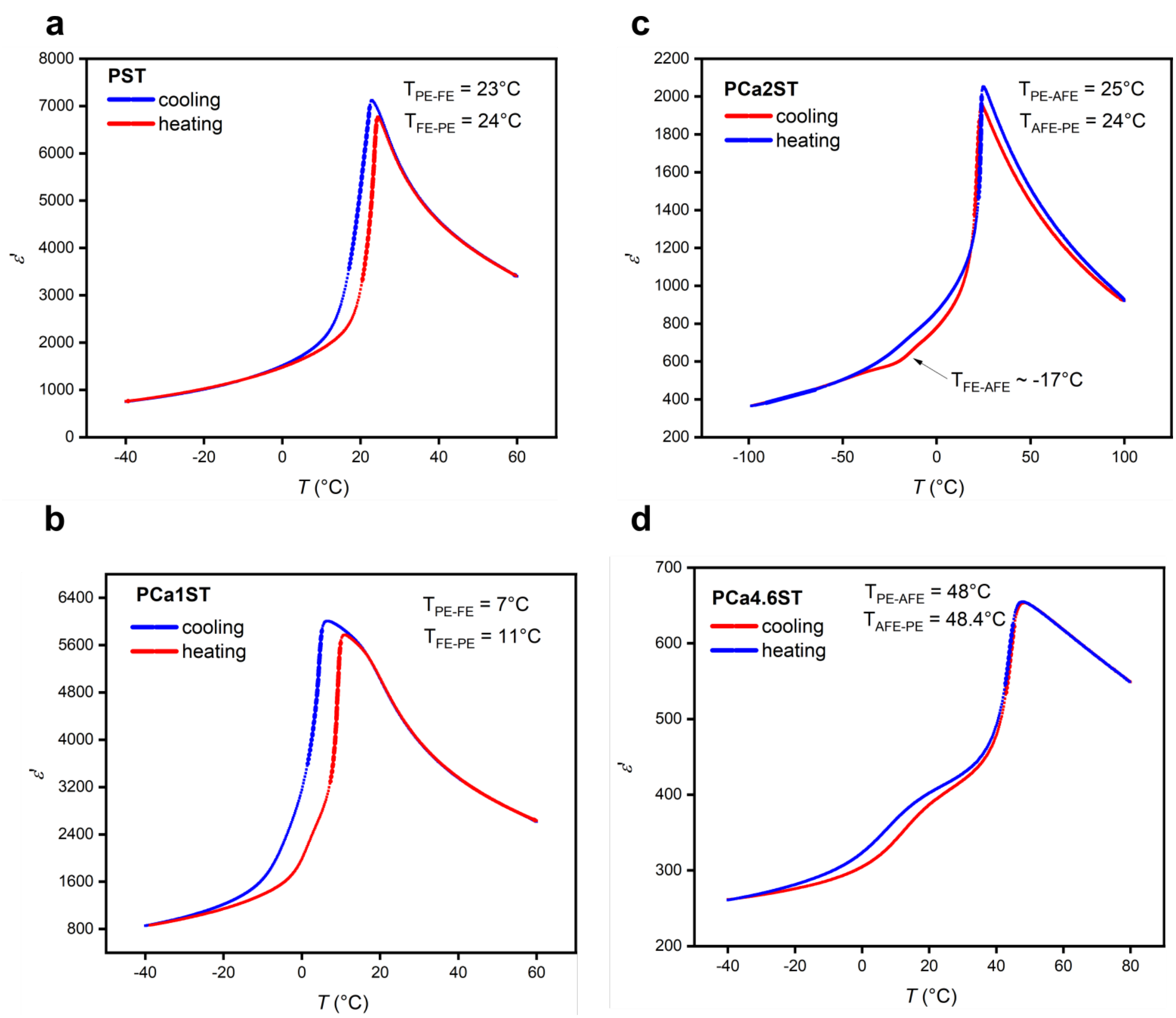

Supplementary Figure 7: **Dielectric measurements of PCaxST.** Dielectric constant ϵ as a function of temperature T. FE, AFE and PE denote, respectively, the ferroelectric, antiferroelectric and paraelectric phases. a) PST, b) PCa1ST, c) PCa2ST, and d) PCa4.6ST.

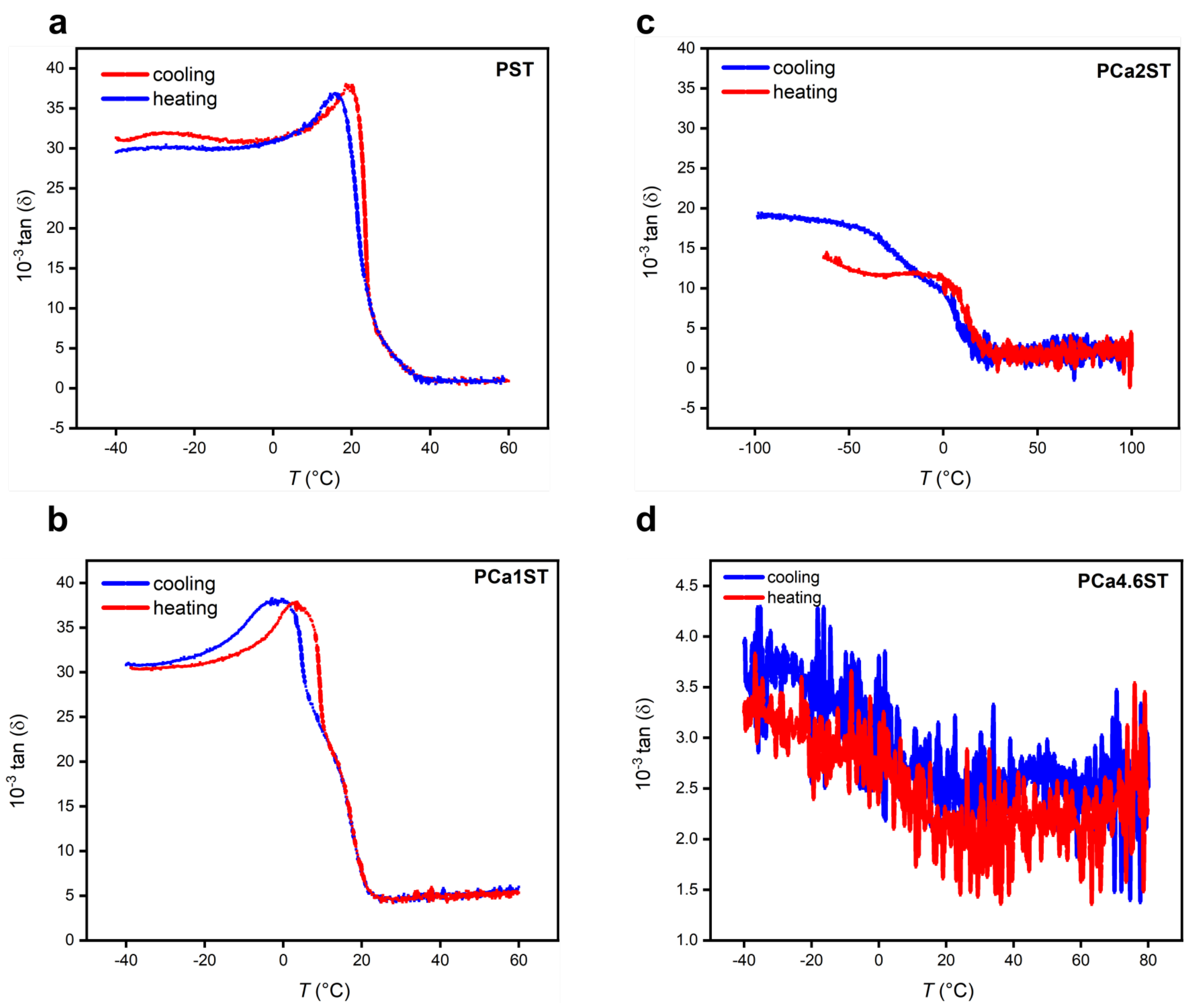

Supplementary Figure 8: **Dielectric losses tan(delta) of PCaxST.** a) PST, b) PCa1ST, c) PCa2ST, and d) PCa4.6ST.

Supplementary Note 5: Thermal properties of pure and Ca-doped PST

Supplementary Table 1: **Thermal properties of pure and Ca-doped PST.** Transition temperatures T_0, latent heat Q_0, and entropy change ΔS_0 for PCaxST compositions at different transitions. FE, AFE and PE refer to respectively ferroelectric, antiferroelectric and paraelectric phases. The columns highlighted on orange and the blue are data collected on heating and cooling respectively. Note that the AFE to FE peak is a field induced transition (see Supplementary Note 6) expected below the temperature range of our equipment.

	**PST**		**PCa1ST**		**PCa2ST**				**PCa4.6ST**	
	FE → PE	PE → FE	FE → PE	PE → FE	FE → AFE	AFE → PE	PE → AFE	AFE → FE	AFE → PE	PE → AFE
T_0 (K)	297	294	282	276	256	297	294		317	317
Q_0 (J/kg)	932	620	544	422	109	275	182		261	277
ΔS_0 (J/kg/K)	3.1	2.1	1.9	1.5	0.4	0.9	0.6		0.8	0.9

Latent heat of pure and Ca-doped PST

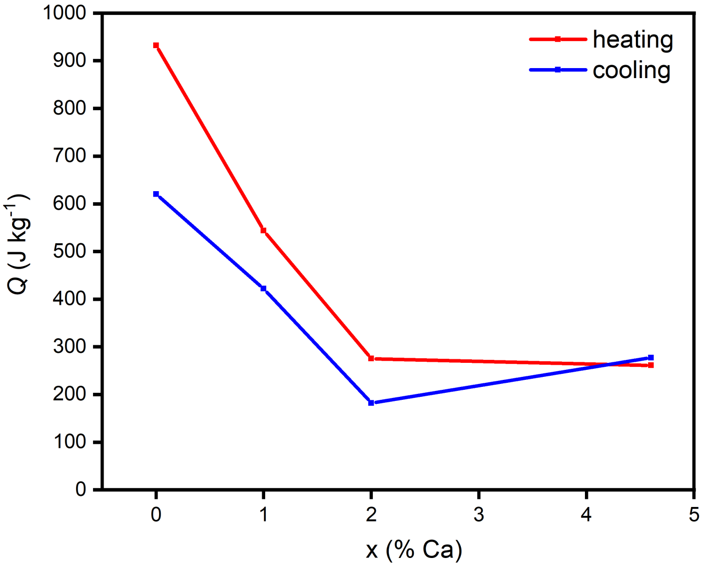

Supplementary Figure 9: **Latent heat Q of Ca-doped PST as a function of calcium content x.**

Samples dimensions pure and Ca-doped PST

Supplementary Table 2: **Thickness and mass of PCaxST samples.**

	**PST**	**PCa1ST**	**PCa2ST**	**PCa4.6ST**
Thickness (μm)	86	82	125	80
Mass (mg)	6.96	11.7	33.02	25.06

Supplementary Note 6: Electrocaloric effect in PCa2ST

DSC measurements on PCa2ST

As illustrated in Figure 2a of the main text, the differential scanning calorimetry (DSC) measurements taken without any bias don't show a transition from ferroelectric (FE) to antiferroelectric (AFE) phase. This transition only becomes apparent during isofield measurements when an electric field of at least 15 kV cm^{-1} is applied. In this experiment, PCa2ST was initially cooled in the presence of an electric field of 16 kV cm^{-1} and then heated without bias to pinpoint its transition temperatures. We observe two distinct thermal anomalies (see Supplementary Figure 10): the first peak appears at 256 K, followed by a second peak at 297 K. The first peak clearly indicates a first-order transition from the FE phase to the AFE phase. Note that the transition temperature from FE to AFE is very close to the value we get from the electrical polarization hysteresis loops (refer to Figure 1c of the main text).

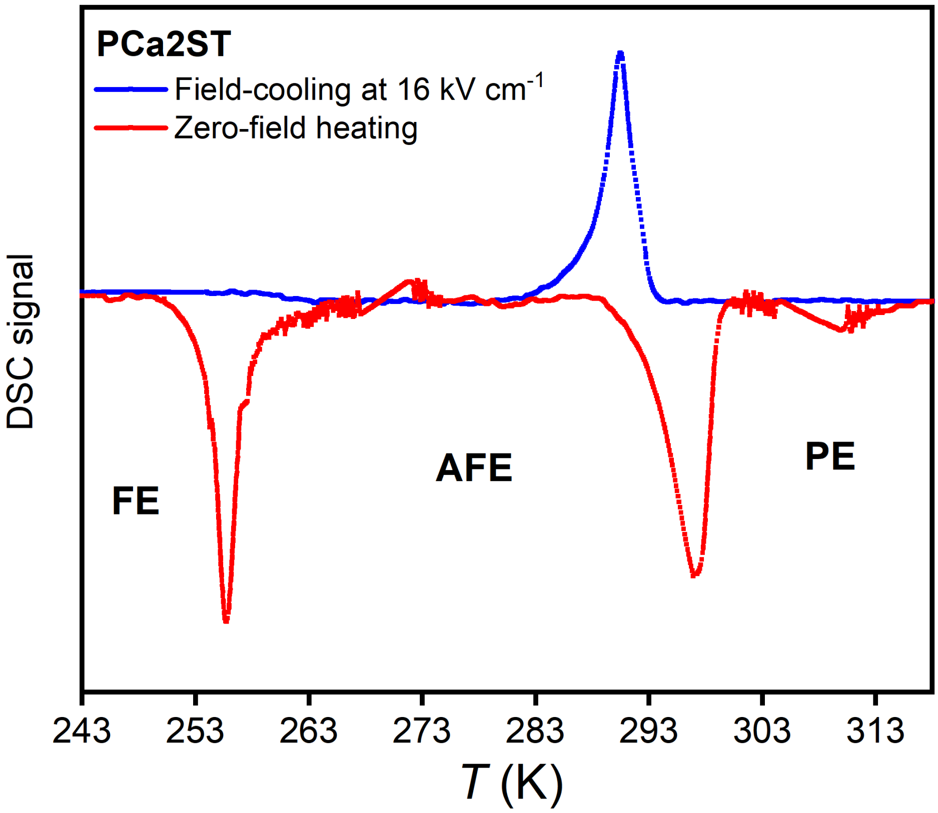

Supplementary Figure 10: **Field cooling and zero-field heating in PCa2ST.** FE, AFE and PE refer to respectively, ferroelectric, antiferroelectric and paraelectric phases. Here, the sample was first field-cooled at 16 kV cm^{-1} and subsequently zero-field heated.

Inverse electrocaloric effect in PCa2ST

The phase diagram of 2%Ca-PST predicts that an inverse electrocaloric effect should occur near the AFE-PE transition at low electric fields (below 30 kV cm^{-1}). Here, we investigated this region in greater detail (blue rectangle in Supplementary Figure 11) using an infrared camera.

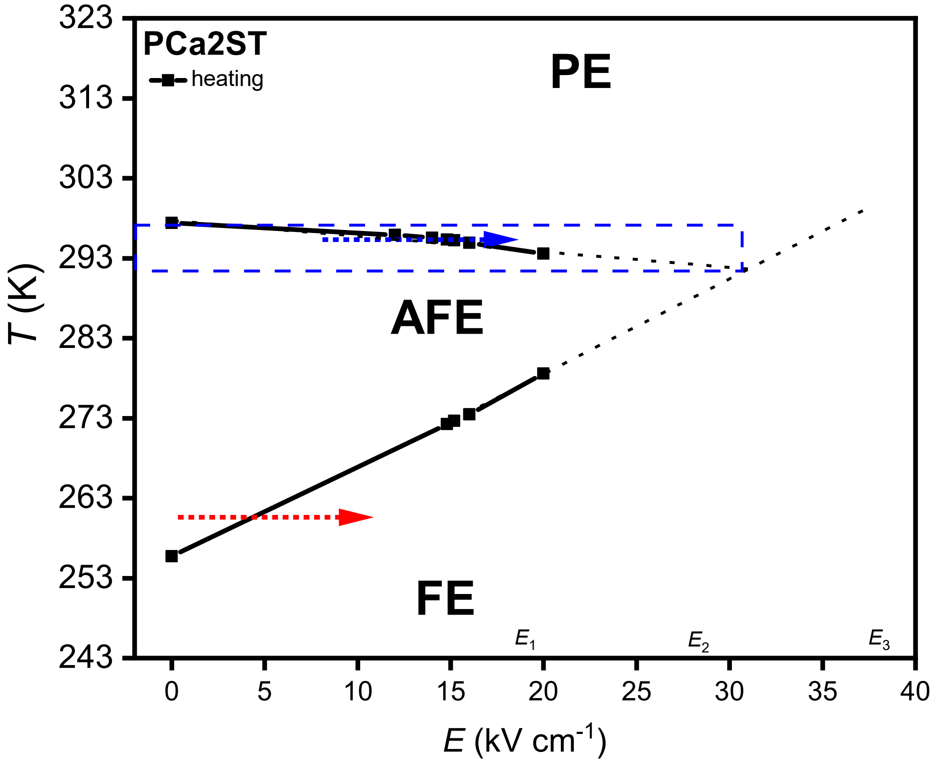

Supplementary Figure 11: **Phase diagram of PCa2ST.**Transition temperature as a function of electric field in 2%Ca-doped PST. FE, AFE and PE refer to the ferroelectric, antiferroelectric and paraelectric phases, respectively. The red arrow describes the conventional electrocaloric effect from the AFE to the FE. The blue arrow describes the inverse electrocaloric effect. The blue rectangle is the region at the vicinity of the AFE-to-PE transition where an inverse electrocaloric is expected.

At 292 K, within the antiferroelectric (AFE) phase, we applied a series of electric fields (19, 23, 27, 28, 30, 34, and 38 kV cm^{-1}). As shown in Supplementary Figure 12, an inverse electrocaloric effect is observed for electric fields below 30 kV cm^{-1}. The magnitude of the inverse electrocaloric temperature change is -0.1 K at 19 kV cm^{-1} and increases to a maximum of -0.25 K at 23 kV cm^{-1}. With further increasing electric field, the magnitude of the inverse EC effect decreases and vanishes near the triple point at 30 kV cm^{-1}.

For electric fields exceeding 30 kV cm^{-1}, the EC response becomes conven-

tional. This behavior arises because high electric fields drive the material into a ferroelectric phase, which possesses a lower entropy than the AFE phase.

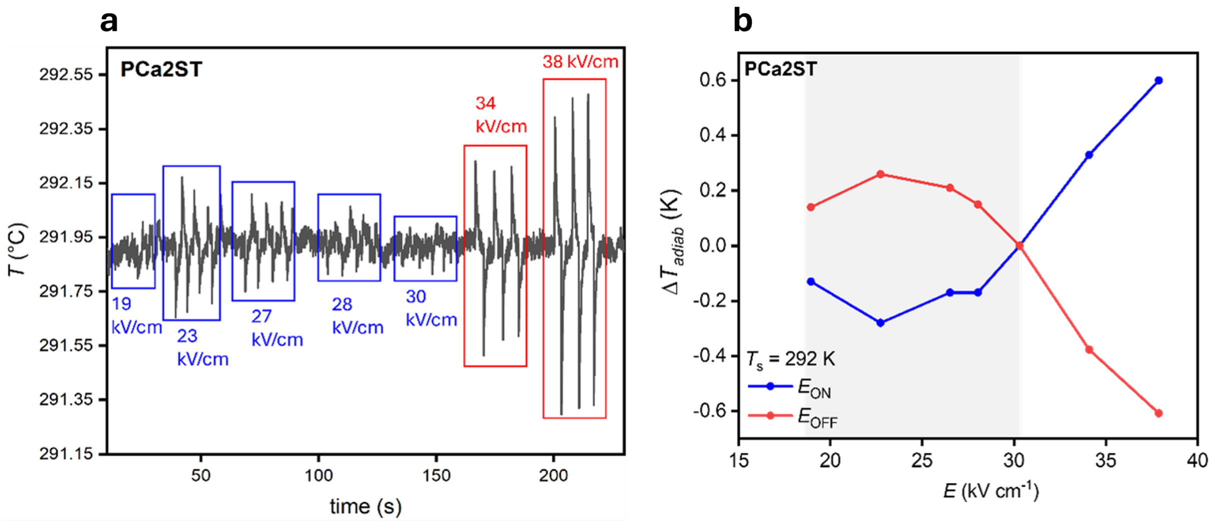

Supplementary Figure 12: **Direct electrocaloric measurements of PCa2ST at low electric fields and around the AFE-to-PE transition. a** The temperature change under different electric bias is shown. **b** the adiabatic temperature change is shown as a function of an applied electric field. The grey area highlights the region where an inverse electro-caloric effect was observed.

These measurements were performed at various electric fields and different starting temperatures and are presented in Supplementary Figure 13. Below the AFE-PE transition temperature, an inverse electrocaloric effect is observed. This response becomes conventional upon crossing the AFE-PE transition temperature and when the applied electric field exceeds 30 kV cm^{-1}. In this regime, the material is in the paraelectric phase, and the application of a strong electric field immediately induces a transition to the ferroelectric phase, thereby resulting in a conventional electrocaloric effect. For the electrocaloric effect at higher electric fields refer to Figure 4c of the main text.

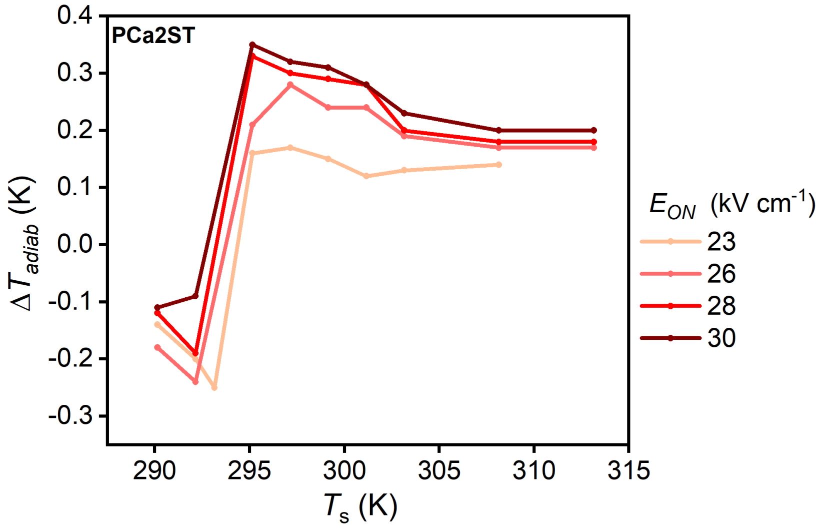

Supplementary Figure 13: **Electrocaloric effect of PCa2ST at different starting temperatures upon application of different electric fields.**

To better understand the tunable electrocaloric effect in PCaST, three scenarios can be described from the phase diagram, corresponding to three different starting temperatures (T_1, T_2, and T_3) and three applied electric fields (E_1, E_2, and E_3). See Supplementary Figure 14.

- The vertical dashed black lines define the three electric fields ($E_1 = 18$, $E_2 = 27$, and $E_3 = 39$ kV cm^{-1}).
- The horizontal short dotted arrows indicate the phase transitions for different electric fields applied at a given starting temperature.
- The blue and red colors represent the inverse and conventional electrocaloric effects, respectively.

First case. At $T_1 = 260$ K, close to the AFE–FE transition, applying E_1, E_2, or E_3 drives the material into the stable FE phase. A conventional electrocaloric effect is expected, as the AFE phase possesses a higher entropy than the FE phase.

Second case. At $T_2 = 294$ K, within the AFE region, applying E_1 or E_2 drives the material from the AFE phase to the PE phase, resulting in an inverse electrocaloric effect since the AFE phase has a lower entropy than the PE phase. When the higher electric field E_3 is applied, the material transitions to the FE phase, producing a conventional electrocaloric effect because the AFE phase has a higher entropy than the FE phase.

Third case. At $T_3 = 297$ K, applying E_1 or E_2 keeps the material in the PE phase, resulting in a conventional electrocaloric effect. At the higher electric field E_3, the material transitions into the FE phase, also exhibiting a conventional electrocaloric effect.

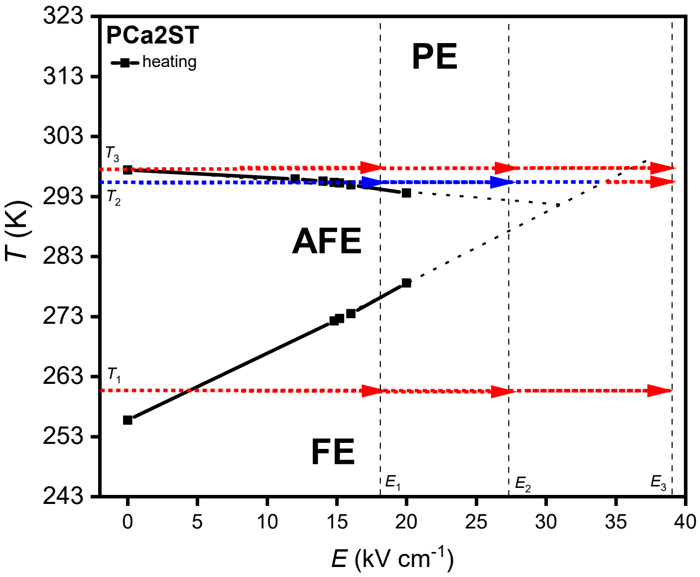

Supplementary Figure 14: **Phase diagram of PCa2ST.** Transition temperature *T* as a function electric field *E*. FE, AFE and PE are respectively the ferroelectric, antiferroelectric and paraelectric phases. T_1, T_2, and T_3 denotes three starting temperatures from which three applied electric fields E_1, E_2, and E_3 are applied. The vertical dashed black lines define the three electric fields ($E_1 = 18$, $E_2 = 27$, and $E_3 = 39$ kV cm^{-1}). The horizontal short dot arrows indicate the phase transition for different electric fields applied at a given starting temperature. The blue and red colors represent the inverse and conventional electrocaloric effects, respectively.

Supplementary Note 7: Electrocaloric effect in PCa4.6ST

Supplementary Figure 15 describes the inverse electrocaloric effect measured in the antiferroelectric materials PCa4.6ST upon application (Supplementary Figure 15b) and removal (Supplementary Figure 15a) of different electric fields. These data were collected using an infrared camera.

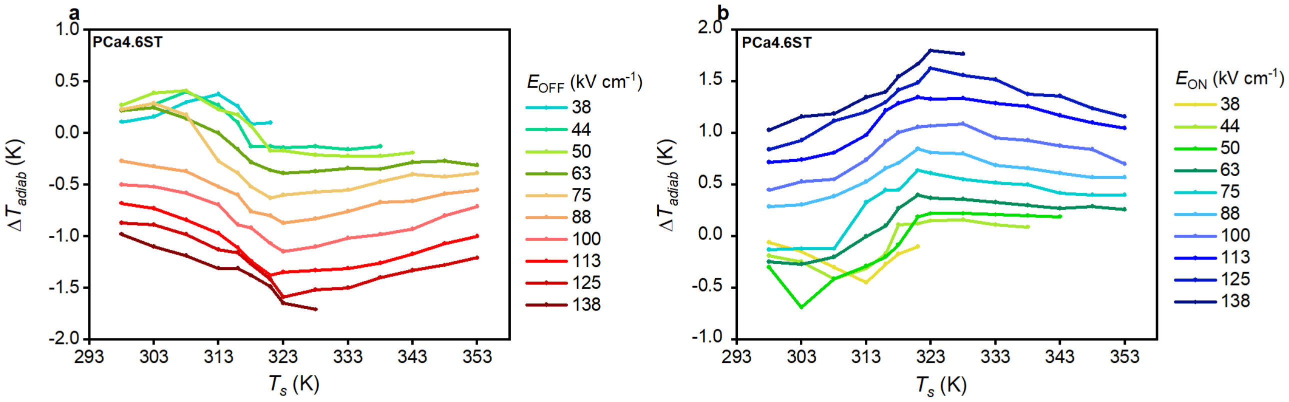

Supplementary Figure 15: **Electrocaloric effect in PCa4.6ST measured using IR camera.** a) Fields off b) fields on

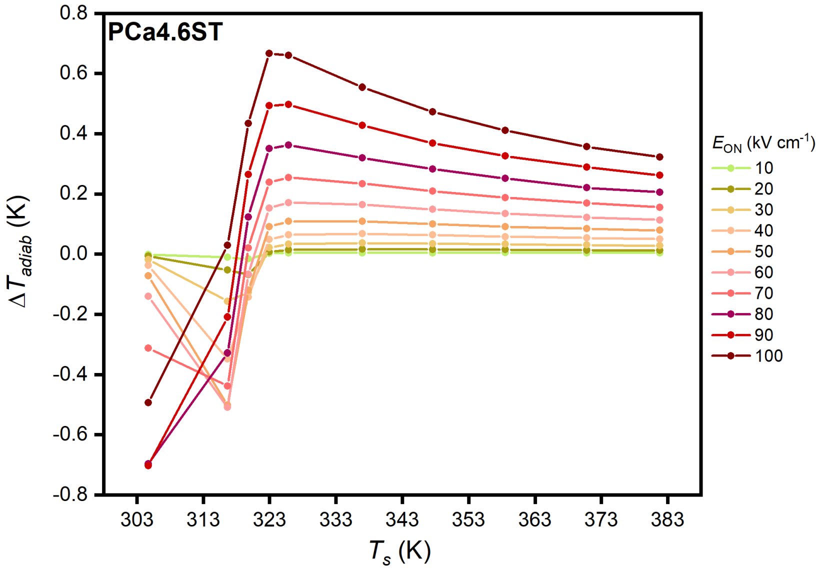

Supplementary Figure 16: **Electrocaloric effect in PCa4.6ST measured using thermistor.**

Supplementary Note 8: Maximum electrocaloric effect in pure and Ca-doped PST

Supplementary Table 3: **Adiabatic temperature change of PCaxST compounds.** ΔT_{adiab} measured at different temperatures and applied electric fields for Ca-doped PST compounds. The adiabatic temperature change was collected with an IR camera.

**PCa$_x$ST**	**T (K)**	ΔT_{adiab} **(K)**	E_{on} **(kV cm^{-1})**
PST	297	4.6	180
PCa1ST	282	3.0	134
PCa2ST	256	2.0	115
PCa2ST	292	-0.25	23
PCa2ST	297	2.5	115
PCa4.6ST	303	-0.6	50
PCa4.6ST	323	1.8	138

Supplementary Note 9: Raman spectra of pure and Ca-doped PST

Raman spectra of undoped PST

The phase transition sequence in our five PCaxST samples was studied by Raman spectroscopy (see Methods section). Supplementary Figure 17a shows the temperature dependence of the Raman spectra of PST without electric field, for temperatures ranging from 283 K to 353 K, i.e. across the known FE to PE transition. The most prominent signature of the phase transition is the emergence of a Raman mode around 420 cm^{-1}, as highlighted in the inset. This occurs at 294 K, very close to the transition temperature measured by DSC (297 K). This peak then becomes sharper and more intense with decreasing temperature. Other signatures are present, although weaker, with the emergence of two modes at 125 and 315 cm^{-1}. These observations are overall consistent with previous reports on Raman spectroscopy of ordered PST, as reported in [7, 8]. Perfectly ordered cubic PST has 4 Raman active modes and the transition to the FE phase with symmetry 3m or 3 results in the activation and splitting of 4 polar T_{1u} modes and one silent T_{1g} mode. However, those signatures are quite weak compared to the disorder-induced Raman modes that tend to activate all modes and broaden lineshapes even with a small degree of disorder. In [8], the modes were assigned using atomistic simulations. Based on this study, the Raman mode around 420 cm^{-1} taken here as the main signature of the FE phase can be attributed to a polar O-B-O bending mode and the weaker modes at 125 and 315 cm^{-1} to translation and rotation modes of the octahedra respectively, all having T_{1u} symmetry in the PE phase. Remarkably, all three show a very good agreement with polar LO modes identified from a previous infrared spectroscopy study [9], which tends to confirm this assignment.

We now turn to Raman spectra of PST under an electric field and at different temperatures. Supplementary Figure 17b shows the spectra at increasing electric field at 295 K, just above the transition, in the cubic phase. For low field values, no visible change occurs. Under the application of 4.7 kV cm^{-1}, a peak appears at 420 cm^{-1} that matches the one identified as the signature of the FE phase. This peak gains in intensity until the highest field applied of 7.8 kV cm^{-1}. The other weaker signature of the FE transition are present as well. Upon removal of the electric field, the spectrum reverts back to its initial state. This experiment was repeated at several temperatures up to 308 K and the same behavior was observed, with the critical field shifting to higher values with increasing temperature. Those results are summarized in Supplementary Figure 18 with a *T*-*E* phase diagram showing the boundary between the PE and the FE phase. This phase diagram is in line with the one obtained with DSC (see Figure 3a of the main text).

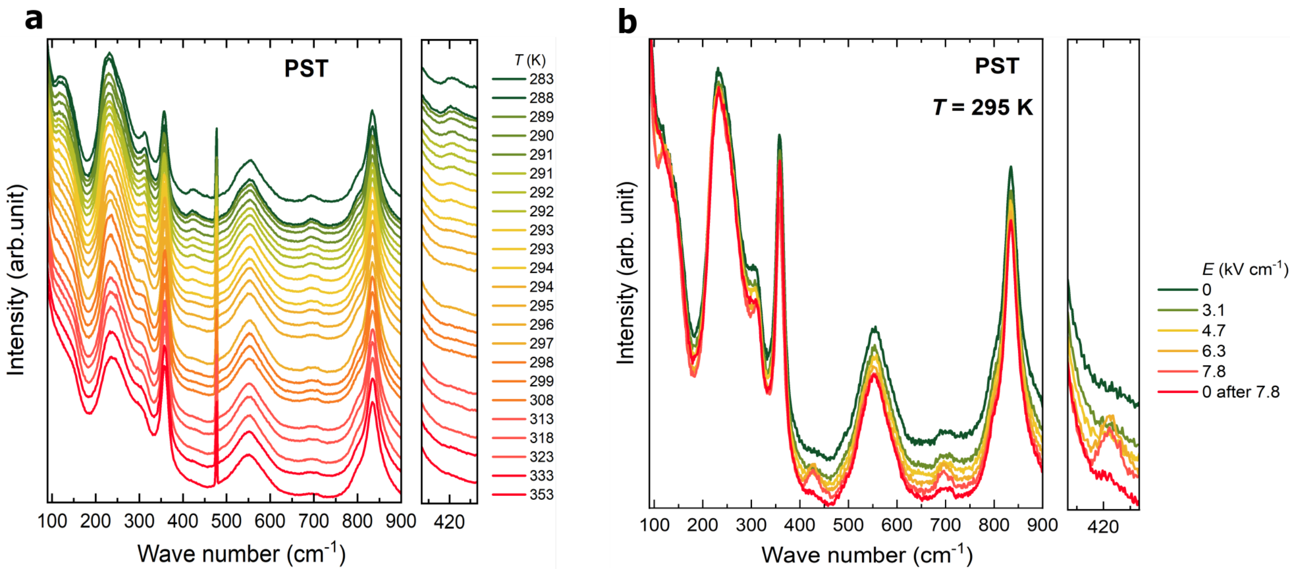

Supplementary Figure 17: **Raman spectroscopy of undoped PST.** (a) Raman spectra of pure PST at temperatures ranging from 283 K to 353 K and zoom in the 420 cm^{-1} Raman shift. (b) Raman spectra of PST pure under different electric fields at 295 K (above the material's transition temperature).

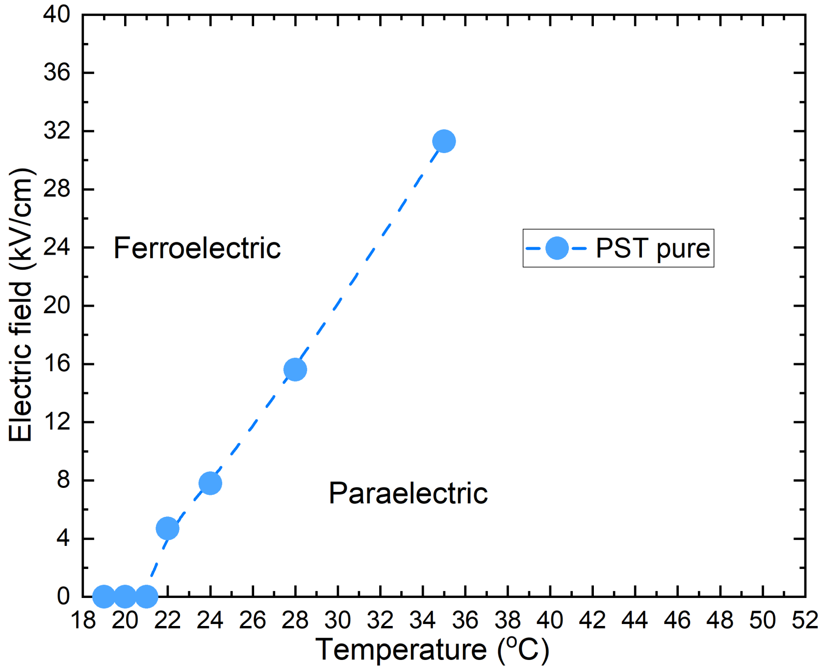

Supplementary Figure 18: **Phase diagram of undoped PST from Raman spectra**

Raman spectra of 2%Ca doped PST

Supplementary Figure 19a shows the Raman spectrum of PCa2ST upon zero-field heating between 283 K and 353 K. Overall, the evolution does not show any strong obvious evidence for phase transitions, and the evolution can at first sight be mostly attributed to thermal broadening. We do observe however the flattening of the peak at the Raman shift 312 cm^{-1} around 295 K, which coincides well with the AFE to PE transition measured in PCa2ST by *P*-*E* loops and confirmed by DSC. A similar observation is made for PCa4.6ST that exhibit such transition as well (see Supplementary Figure 21).

The situation is markedly different upon application of an electric field, as shown for PCa2ST in Supplementary Figure 19b. At 223 K, the application of an electric field of 28 kV cm^{-1} causes the same changes observed in pure PST when inducing

the FE phase: strong activation of the polar mode at 420 cm^{-1}, as well as the modes around 140, 310 and 700 cm^{-1}. In fact, the mode at 140 cm^{-1} (BO_3 translations) appears even stronger due to the relatively lower intensity at low frequencies – less quasi-elastic scattering. As shown in the zoom-in of Supplementary Figure 19b and indicated by blue arrows, this signature remains present up to 253 K, where it disappears. Indeed, the appearance of the peak at 420 cm^{-1} indicates the FE phase induced by an electric field in PCa2ST and its disappearance demonstrated the transition of PCa2ST from a FE to an AFE phase which occurs at 253 K. Note that this transition temperature from FE to AFE is close to the transition temperature (258 K) observed by isofield measurements (Figure 3c of the main text).

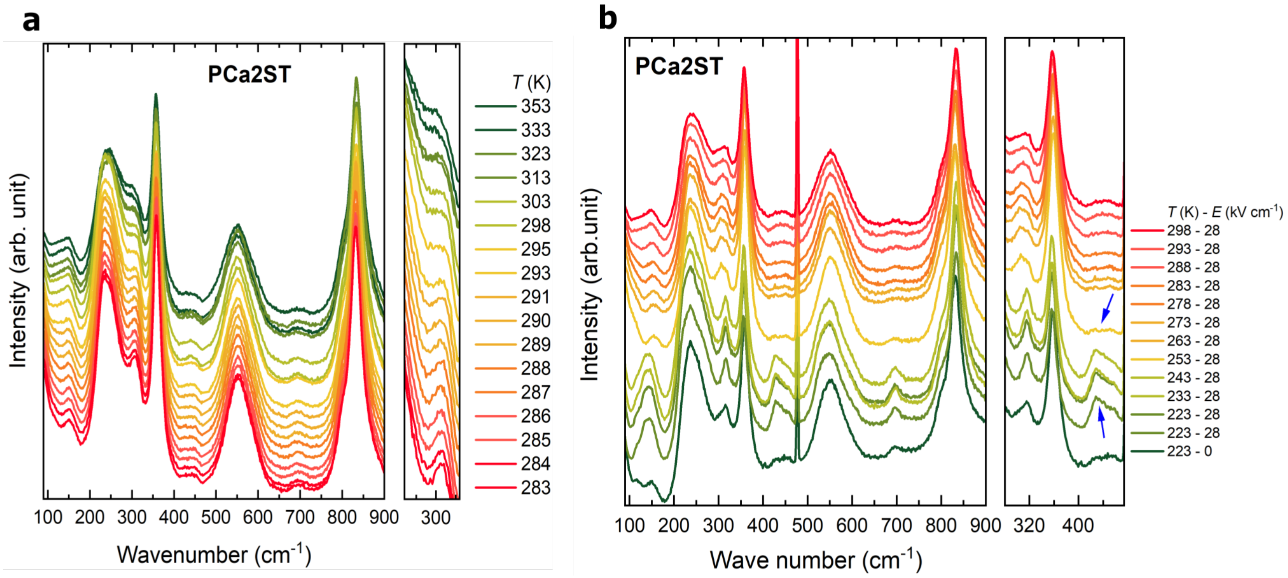

Supplementary Figure 19: **Raman Spectra of PCa2ST.**(a) Raman spectra of PCa2ST at different temperatures and zoom in the 312 cm^{-1} Raman shift. (b) Raman spectra of PCa2ST under the application of an electric field and zoom in the 420 cm^{-1} Raman shift. An electric field of 28 kV cm^{-1} was applied in PCa2ST, subsequently Raman spectra were collected at different temperatures (from 223 K to 298 K). The blue arrows indicate the appearance of a peak at low temperatures (223 K) and at 420 cm^{-1} under the application of the field and the disappearance of this peak at 253 K.

Raman spectra of 4.6%Ca-doped PST

In general, Ca-doping induces some additional disorder that may activate Raman modes. This is demonstrated in Supplementary Figure 20 where all samples are compared at a temperature of 323 K, i.e. in the same PE phase. With increasing Ca concentration, we observe that Raman modes broaden and decrease in intensity; we also observe the emergence of a small bump around 450 cm^{-1}, close to but clearly distinct from the mode at 420 cm^{-1} taken as a signature for the FE phase.

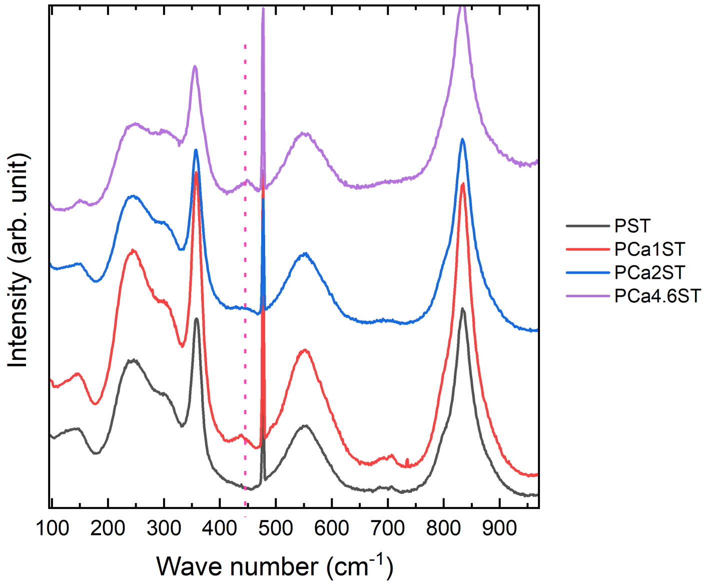

Supplementary Figure 20: **Calcium effect in Raman Spectra at 323 K** Temperature dependence of Raman spectra was carried out in the Ca-doped PST in the same phase (cubic).

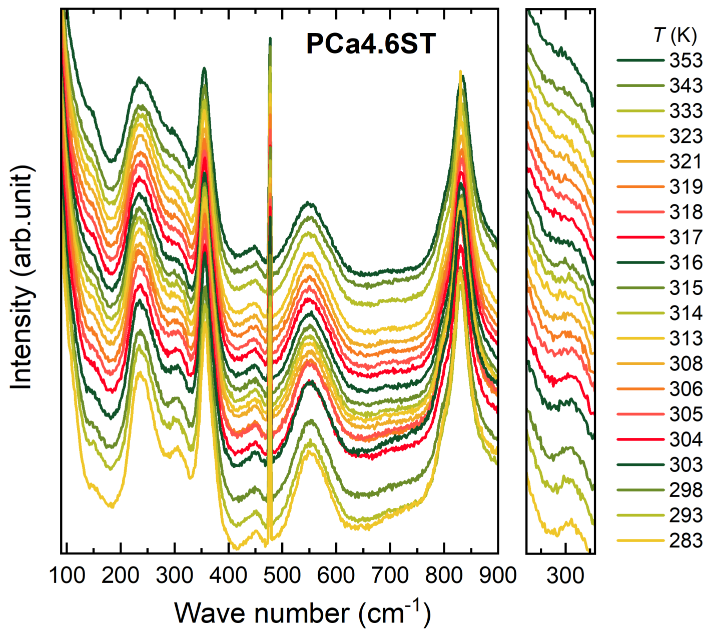

Supplementary Figure 21: **Raman Spectra of PCa4.6ST.**

Supplementary Note 10: Piezoresponse force microscopy

Supplementary Figure 22 shows the AFM topography images and PFM images of PST and PCa4.6ST. For PCa4.6ST, the PFM images under low AC excitation reveal only noise (Supplementary Figure 22b), indicating the absence of piezoelectric activity. In other words, the sample is non-polar. In contrast, the PFM image of undoped PST (Supplementary Figure 22d) shows distinct domain contrast, characteristic of a ferroelectric material. From the comparison of the topography and PFM phase images, we can conclude that the topography crosstalk is negligible. The ferroelectric domains features in PFM phase images (b, d) show no direct correlation with any topographic features.

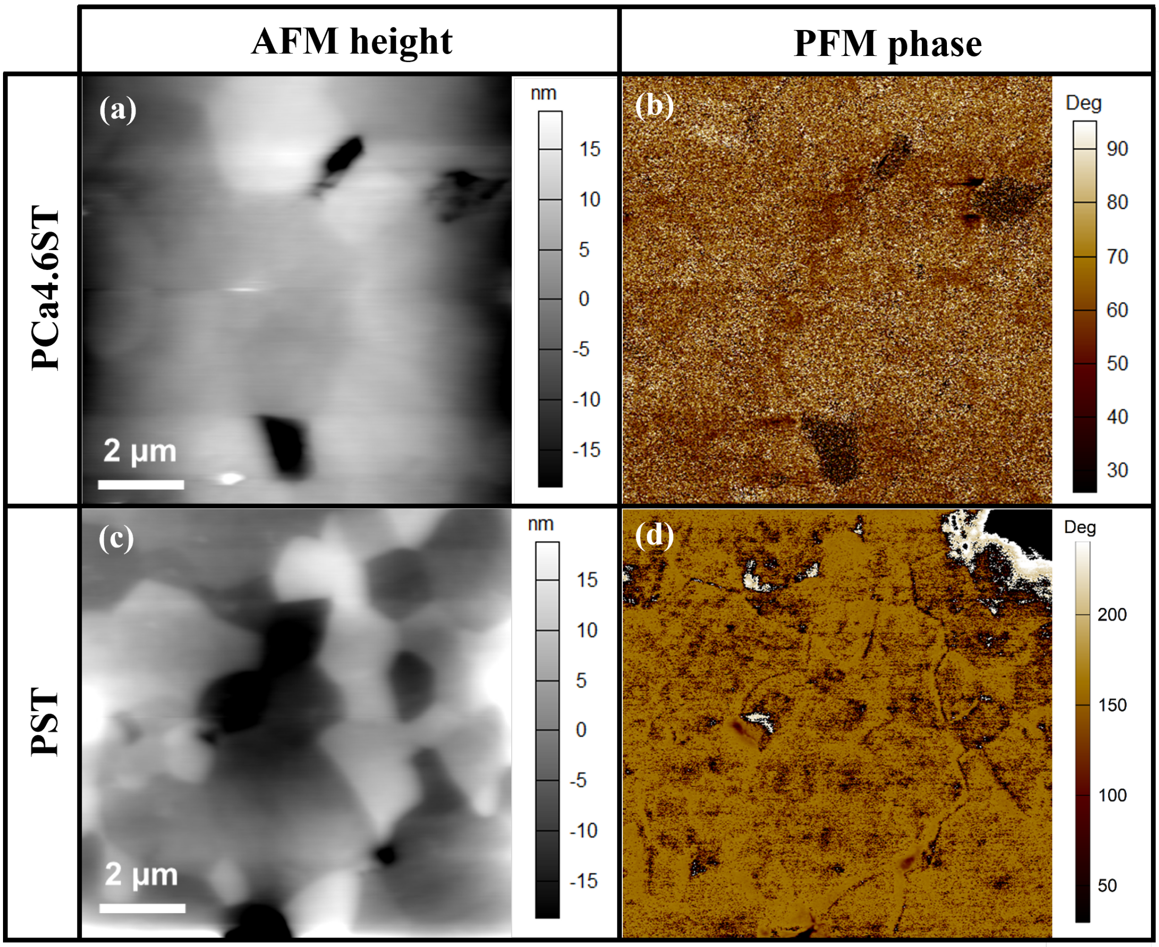

Supplementary Figure 22: **Piezoresponse force microscopy (PFM) of PST and PCa4.6ST.** AFM topography height (a, c) and PFM phase images (b, d) of PST and PCa4.6ST. Panels (a, b) correspond to PCa4.6ST, while panels (c, d) correspond to PST.

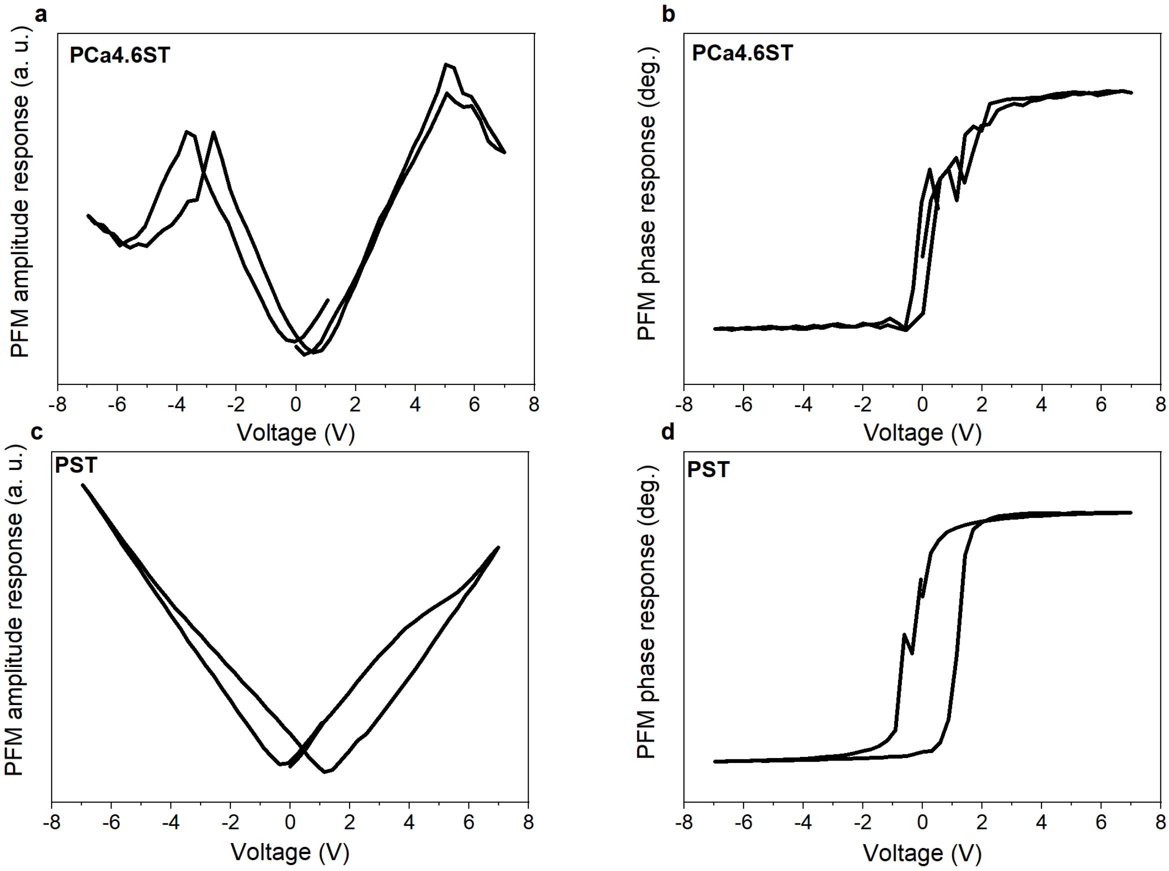

Supplementary Figure 23: **ON-electric field measurements**. Local PFM amplitude hysteresis loops (a and c), and PFM phase hysteresis loops (b and d). Panels (a-b) correspond to PCa4.6ST, while panels (c-d) correspond to PST

Supplementary Figure 23 presents the PFM amplitude loops, and PFM phase loops for both PST and PCa4.6ST samples. The PFM amplitude loop of PST (Supplementary Figure 23c) exhibits a butterfly-type hysteresis with two minima and a non-zero amplitude at zero bias, indicating a stable remanent ferroelectric polarization. This is confirmed by the PFM phase response (Supplementary Figure 23d), which shows an hysteresis loop. These features are consistent with ferroelectric behavior and confirm the polarization hysteresis loops previously measured in PST (see Figure 1a). On the other hand, the off-electric field measurements (Supple-

mentary Figure 24) confirm that PCa4.6ST is not ferroelectric. Moreover, the bias-on PFM amplitude loop of PCa4.6ST (Supplementary Figure 23a) displays four maxima, which aligns well with the features observed in the polarization–electric field loops. This is further confirmed with a double hysteresis-like loop in the PFM phase response (Supplementary Figure 23b). These are clear signatures of antiferroelectric behaviour as demonstrated in [10] and [11].

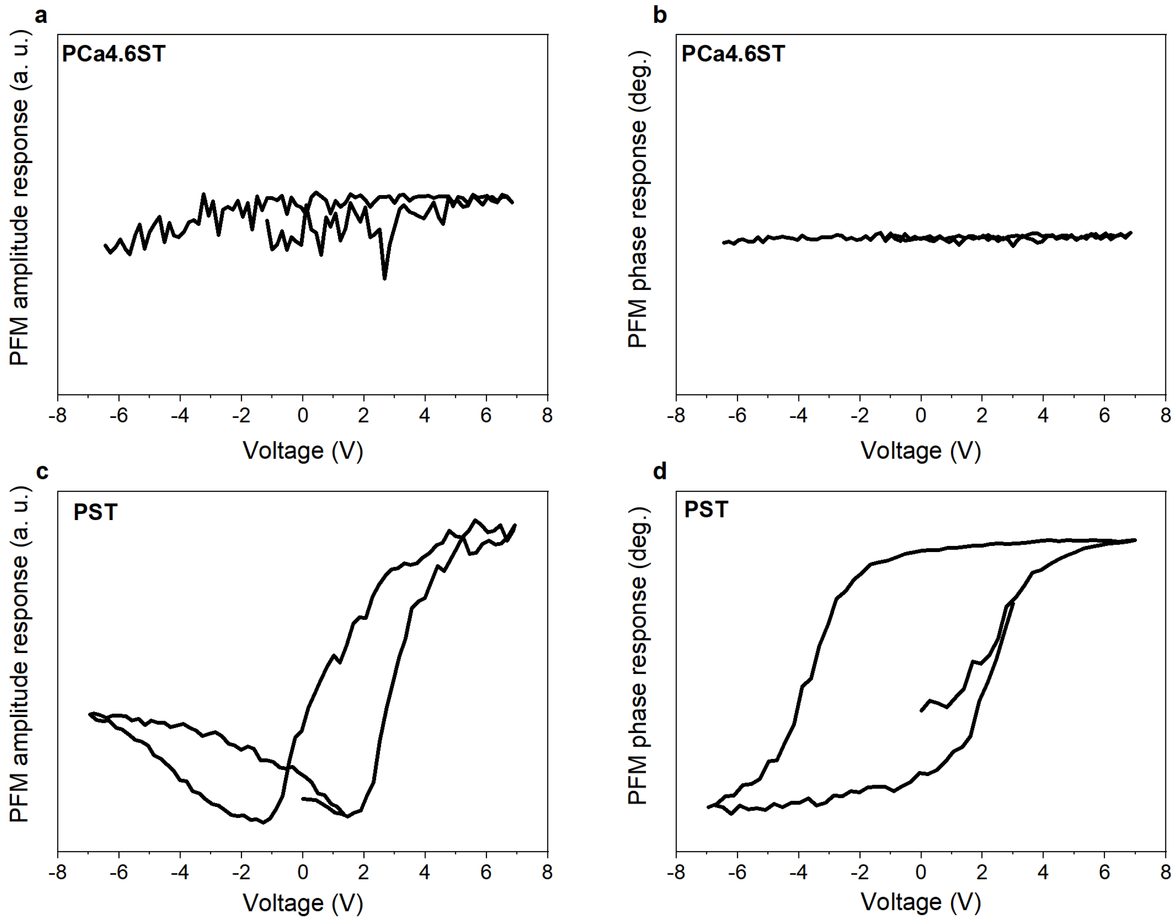

Supplementary Figure 24: **OFF-electric field measurements**. Local PFM amplitude hysteresis loops (a and c), and PFM phase hysteresis loops (b and d). Panels (a-b) correspond to PCa4.6ST, while panels (c-d) correspond to PST

Supplementary Note 11: Density Functional Theory Calculations

Structure of pure and Ca-doped PST

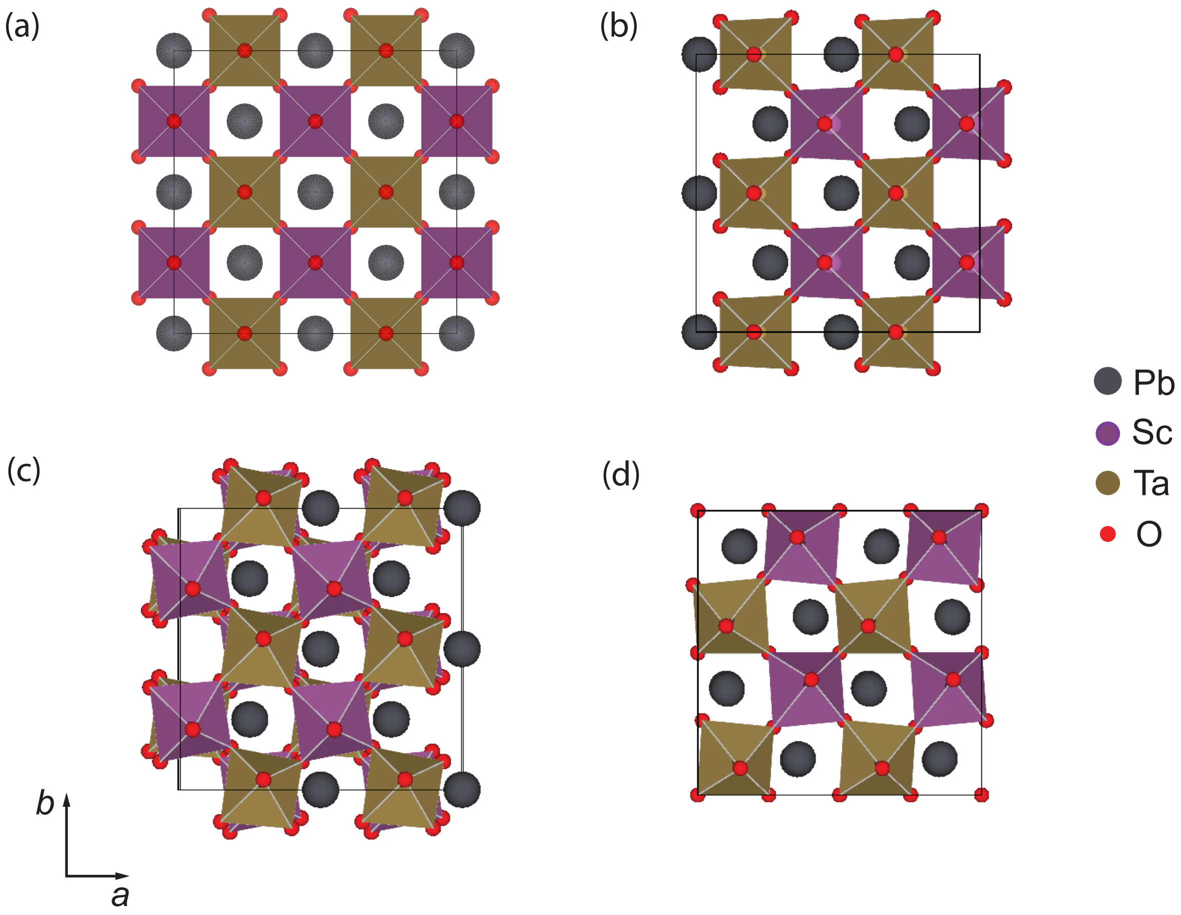

Supplementary Figure 25: **Structural polymorphs of pure PST considered in this work.** Panels (a), (b), (c) and (d) show c, rI, rII and AFE polymorphs, respectively. The lattice vectors a_{sc}, b_{sc} and c_{sc} of the 80 atom cells are related to the a_p, b_p and c_p lattice vectors of 5-atom perovskite cell as follows: $a_{sc} = 2a_p + 2b_p$; $b_{sc} = -2a_p + 2b_p$; $c_{sc} = 2c_p$.

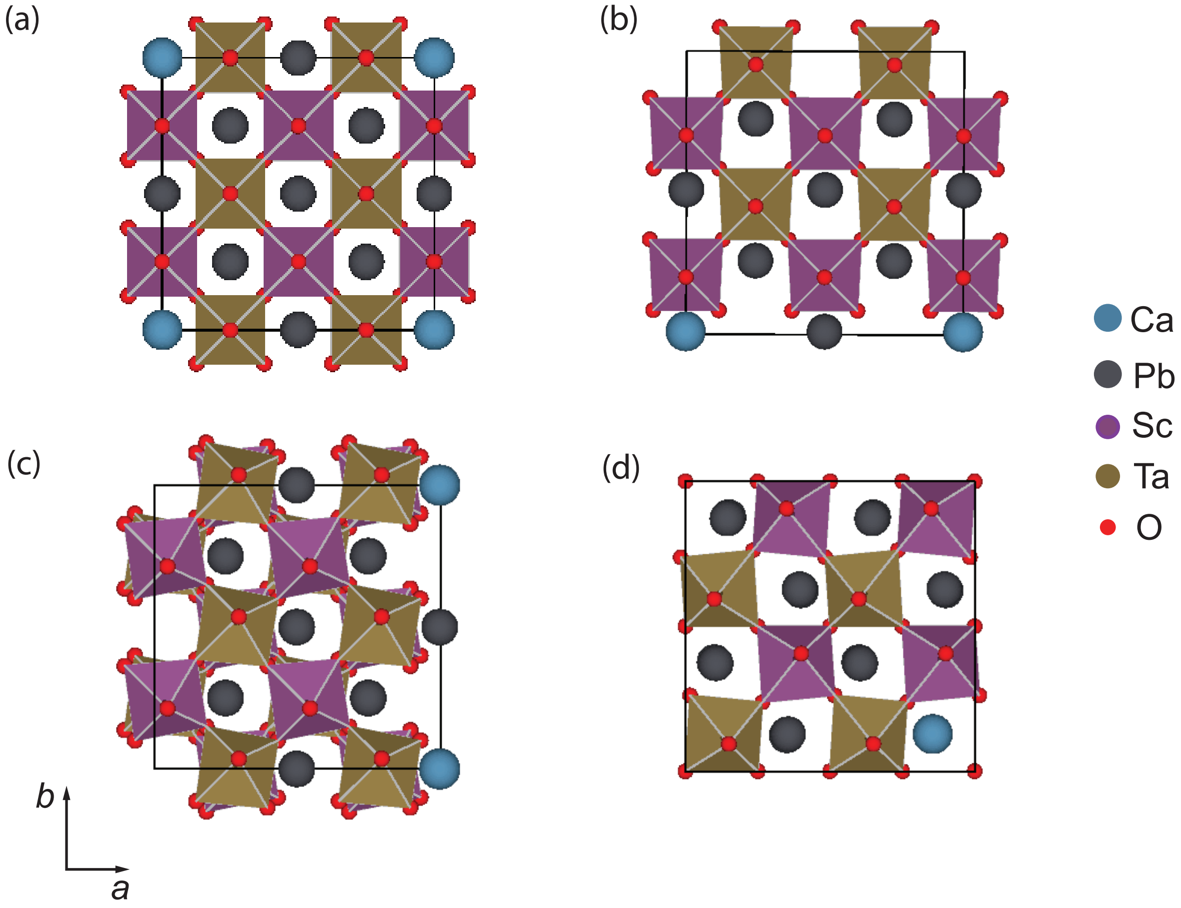

Supplementary Figure 26: **Structural polymorphs of PST doped with 6.25% of Ca considered in this work.** Panels (a), (b), (c) and (d) show c, rI, rII and AFE polymorphs, respectively.

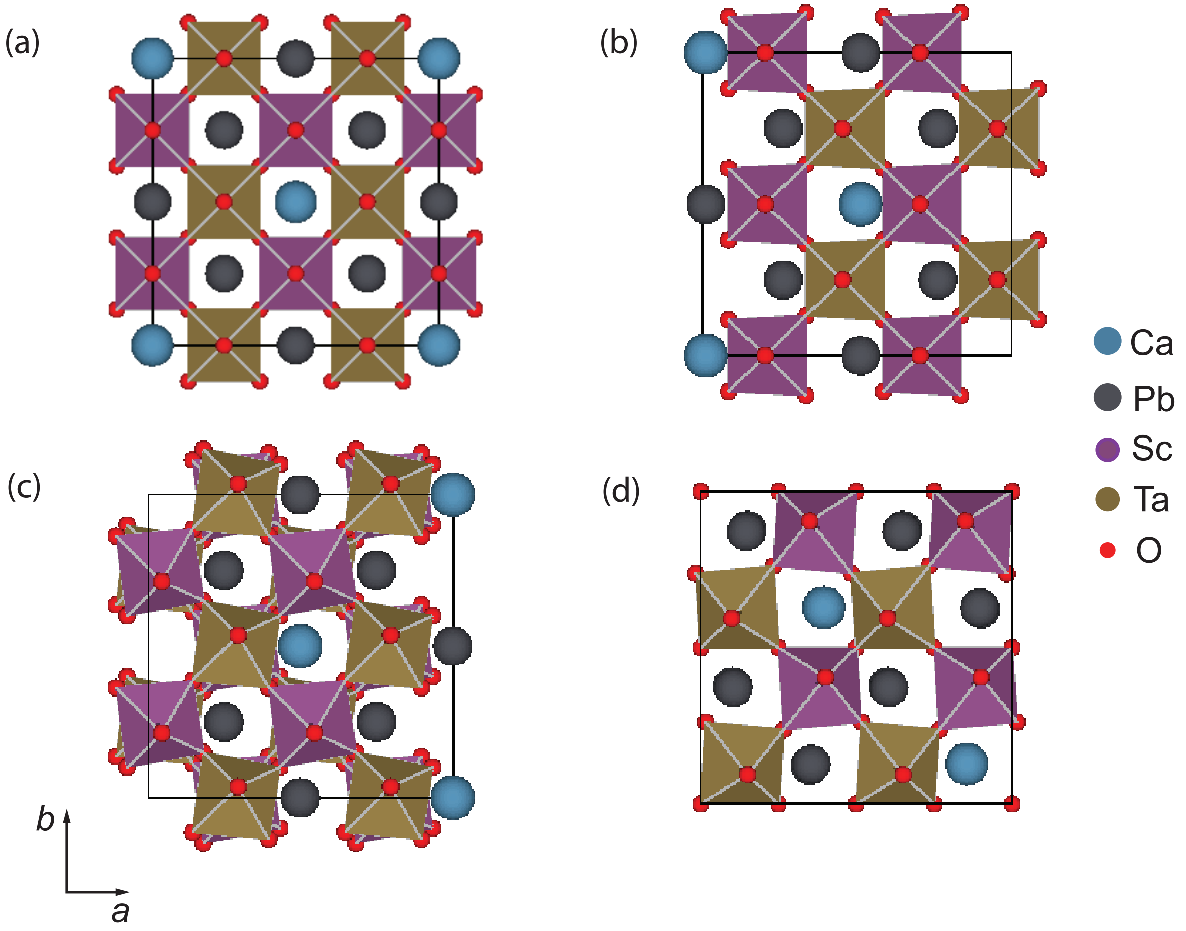

Supplementary Figure 27: **Structural polymorphs of PST doped with 12.5% of Ca considered in this work.** Panels (a), (b), (c) and (d) show c, rI, rII and AFE polymorphs, respectively.

Simulated X-ray diffraction pattern of pure PST

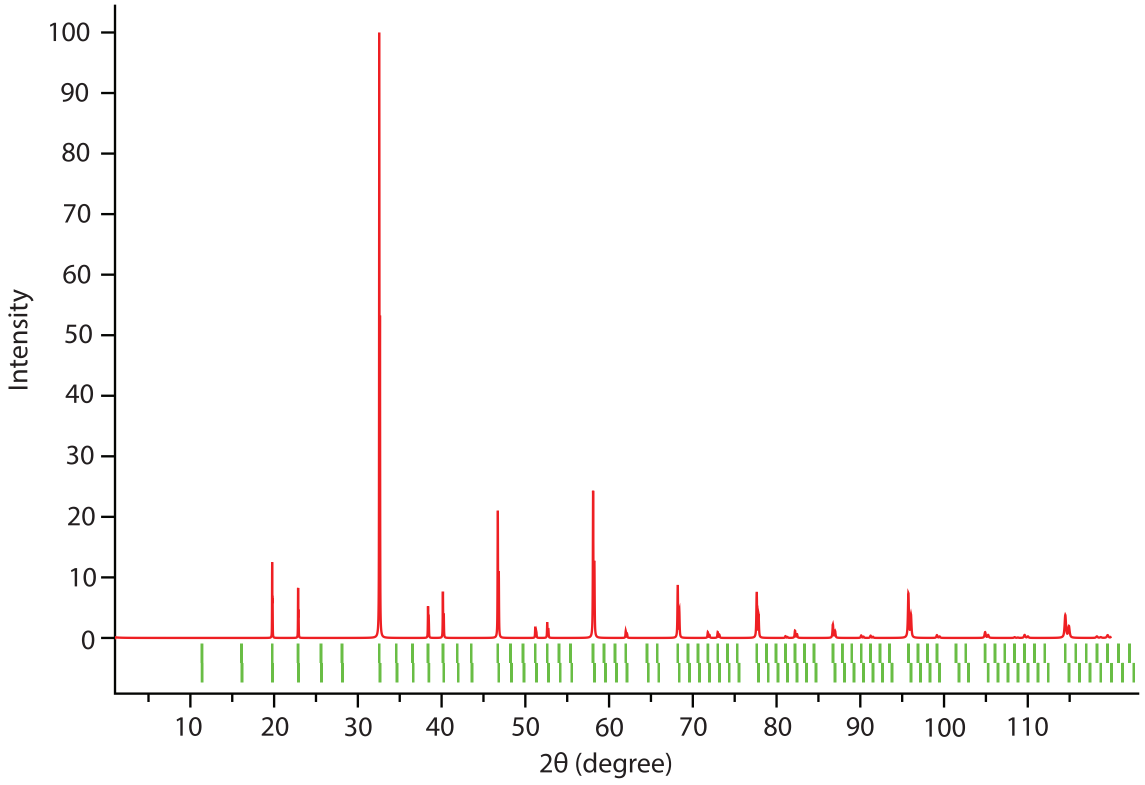

Supplementary Figure 28: **Theoretical X-ray diffraction (XRD) pattern obtained for cubic phase of pure PST with perfect Sc/Ta ordering.**

Supplementary Table 4: **Intensity ratio I_{111}/I_{200} calculated for cubic phase of pure and Ca-doped PST with perfect Sc/Ta order.**

Ca Concentration	I_{111}/I_{200}
0%	1.37
1.56%	1.47
3.125%	1.59
12.5%	2.60

Energies calculations

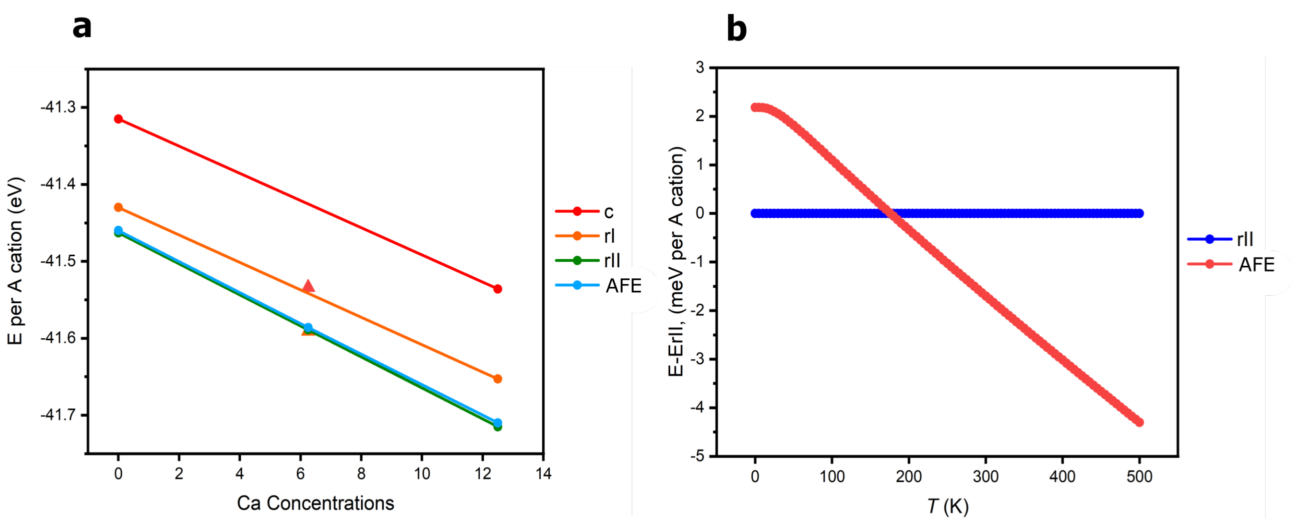

Supplementary Figure 29: **The calculated energies of pure and Ca-doped PST polymorphs.** Panel (a) shows the DFT energies of Ca-doped PST polymorphs as functions of Ca concentration. For 6.25% Ca doping, the c and rI starting configurations relax to lower symmetry phases whose energies are shown with red and orange triangles, respectively (see the main text for details). The phase indicated by the orange triangle is identical (within numerical error) to rII phase. Panel (b) shows the free energies of rII and AFE phases of pure PST as functions of temperature.

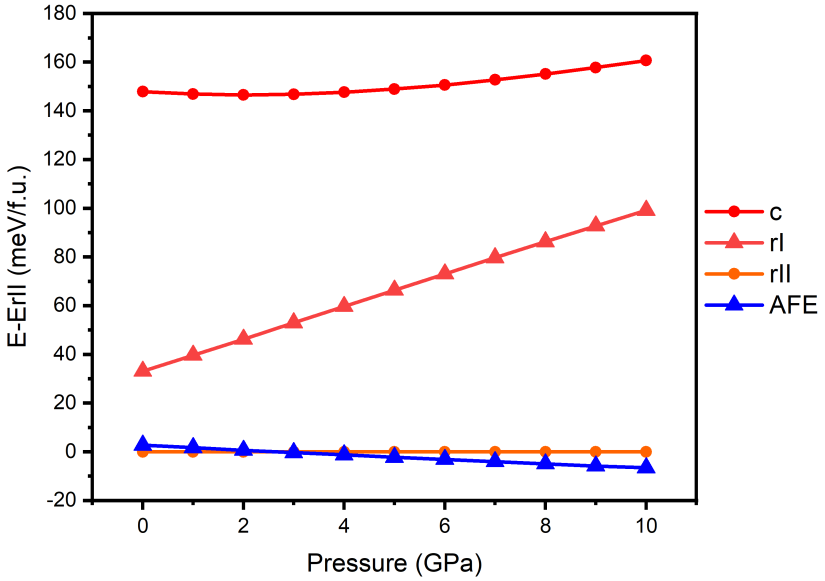

Supplementary Figure 30: **The energies of c, rI, rII and AFE phases as functions of hydrostatic pressure, calculated using DFT.** The energy of rII phase is taken as zero.

Phonons and zero-point contributions

Since the difference in the DFT energies of rII and AFE phases of pure and Ca-doped PST are very small (rII is the lowest energy phase), we check whether the polymorph hierarchy can be affected by zero-point energy (E_{ZP}) contributions. Here we focus on the case of pure PST.

First, we compute phonon dispersion for rII and AFE phases. The results are shown in Supplementary Figure 31. One can see that rII phase shows no instabilities, while AFE phase has one unstable phonon branch in the vicinity of the B point with q=(0,0,0.5). Then, we compute E_{ZP} for both polymorphs (see Methods for details). The resulting values together with the Kohn-Sham (E_{KS}) energies and the total energies (E_{tot} = E_{KS} + E_{ZP}) are summarized in Supplementary Table 5. We find that the addition of E_{ZP} to E_{KS} does not change the hierarchy of these polymorphs, and rII remains to be the lowest energy state.

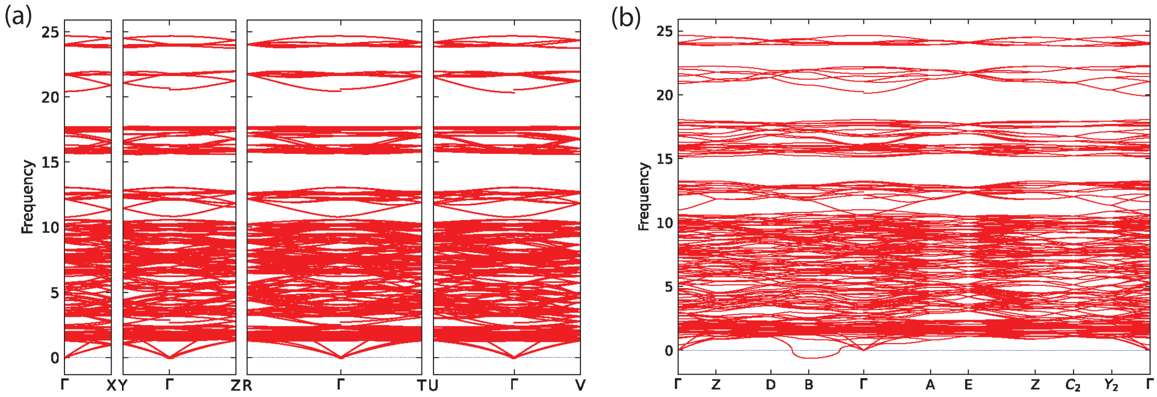

Supplementary Figure 31: **Phonon dispersions of (a) rII and (b) AFE phases of PST.** Phonon frequencies are given in THz.

Supplementary Table 5: **Energy values for different configurations.** The Kohn-Sham energies (E_{KS}), the zero-point energies (E_{ZP}) and the total energies (E$_{tot}$) computed for rII and AFE phases of PST. All energy values are given for 80 atom cell and are in eV.

	$E_{\textbf{KS}}$	$E_{\textbf{ZP}}$	$E_{\textbf{tot}}$
**rII**	-663.4100	4.2659	-659.1441
**afe**	-663.3667	4.2576	-659.1091

Effect of Ca doping and hydrostatic pressure on the crystal structure and relative stability of $PbSc_{0.5}Ta_{0.5}O_3$ polymorphs.

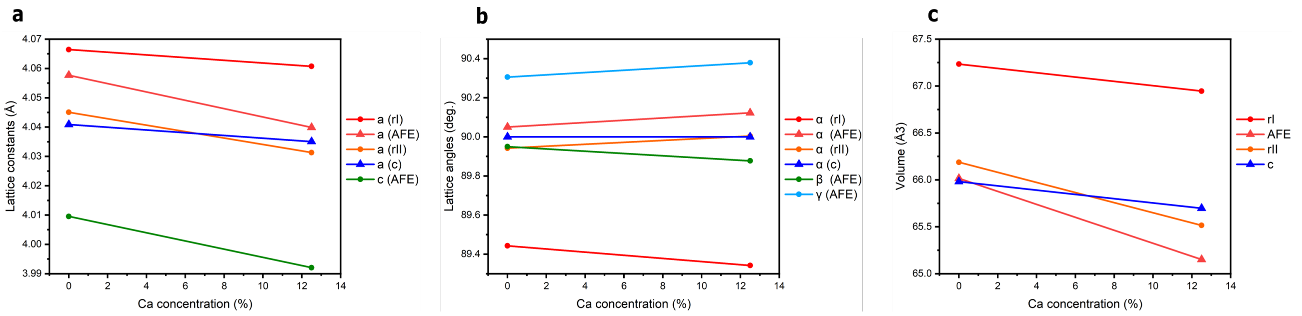

Supplementary Figure 32: **Lattice parameters and volumes of the 5-atom pseudocubic perovskite cell for the c, rI, rII, and AFE phases as functions of Ca doping concentration, as computed using DFT.** Panel (a) shows a, b(a=b) and c lattice constants; panel (b) - α, β, and γ angles; panel (c) - the volume.

We summarize the lattice parameters of c, rI, rII, and AFE phases of pure and 12.5% Ca-doped PST in Supplementary Table 6 and Supplementary Figure 32. One can see that 12.5% Ca doping leads to an approximately 0.14% reduction in the lattice constants of the c and rI phases (corresponding to a 0.43% reduction in the 5-atom cell volume). For the rII and AFE phases, the lattice parameter reduction is more pronounced: 0.34% and 0.44%, respectively (corresponding to 1.02% and 1.31% reductions in volume, respectively).

The results for the pure PST under hydrostatic pressure are presented in Supplementary Table 7 and Supplementary Figure 33. One can see that an application of P=10GPa reduces the lattice constants of the rI, rII and AFE phases by approximately 1.96% (about 5.7% reduction in volume), while the cubic phase shows a 1.62% reduction in lattice constants (about 4.8% in volume).

By comparing the evolution of the cell volumes under Ca doping and hydrostatic pressure, we estimate that the effect of 12.5% Ca doping corresponds to applying

Supplementary Table 6: Lattice parameters and volumes of the 5-atom pseudocubic perovskite cell for the c, rI, rII, and AFE phases of pure and 12.5% Ca-doped $PbSc_{0.5}Ta_{0.5}O_3$, computed using DFT. a, b, and c are in Å; α, β, and γ are in degrees; V is in Å^3.

	c	rI	rII	AFE
**Pure PST**				
a	4.0409	4.0665	4.0451	4.0577
b	4.0409	4.0665	4.0451	4.0577
c	4.0409	4.0665	4.0451	4.0095
α	90.000	89.443	89.942	90.050
β	90.000	89.443	89.942	89.950
γ	90.000	89.443	89.942	90.306
V	65.981	67.234	66.188	66.015
**12.5% Ca-doped PST**				
a	4.0350	4.0607	4.0313	4.0399
b	4.0350	4.0607	4.0313	4.0399
c	4.0350	4.0607	4.0313	3.9921
α	90.000	89.342	90.004	90.123
β	90.000	89.342	90.004	89.877
γ	90.000	89.342	90.004	90.379
V	65.697	66.946	65.515	65.151

0.8 GPa in the c phase, 0.65 GPa in rI, 1.45 GPa in rII, and 1.92 GPa in the AFE phase.

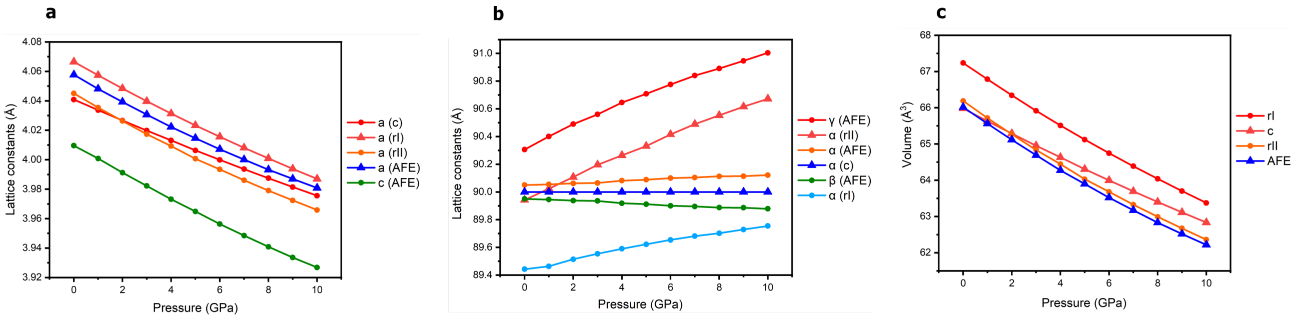

Supplementary Figure 33: **Lattice parameters and volumes of the 5-atom pseudocubic perovskite cell for the c, rI, rII, and AFE phases as functions of hydrostatic pressure, computed using DFT.** Panel (a) shows a, b (a=b) and c lattice constants; panel (b) - α, β, and γ angles; panel (c) - the volume.

Supplementary Table 7: Lattice parameters and volumes of the 5-atom pseudocubic perovskite cell for the c, rI, rII, and AFE phases of pure PST under 5 GPa and 10 GPa hydrostatic pressure, computed using DFT. a, b, and c are in Å; α, β, and γ are in degrees; V is in Å^3.

	**5 GPa**			
	c	rI	rII	AFE
a	4.0063	4.0233	4.0007	4.0146
b	4.0063	4.0233	4.0007	4.0146
c	4.0063	4.0233	4.0007	3.9649
α	90.000	89.622	90.331	90.088
β	90.000	89.622	90.331	89.912
γ	90.000	89.622	90.331	90.708
V	64.305	65.121	64.028	63.898
	**10 GPa**			
a	3.9755	3.9870	3.9658	3.9808
b	3.9755	3.9870	3.9658	3.9808
c	3.9755	3.9870	3.9658	3.9268
α	90.000	89.755	90.672	90.122
β	90.000	89.755	90.672	89.878
γ	90.000	89.755	90.672	91.004
V	62.833	63.374	62.361	62.217

In Supplementary Figure 29a and 30, we show the relative stability of the considered phases under Ca doping and hydrostatic pressure, as predicted by our DFT calculations. We find that AFE phase becomes more energetically favorable relative to the rII phase at pressures around 3 GPa, whereas rII remains the ground state up to at least 12.5% Ca concentration.

These results indicate that the experimentally observed AFE phase in Ca-doped PST samples (with 2 to 4.6% Ca concentration) cannot be explained solely by the volume reduction caused by doping. It is likely that local relaxation around Ca cations, along with temperature effects, plays a significant role in stabilizing the AFE phase.

Effect of Ca doping and hydrostatic pressure on the distortion mode amplitudes of $PbSc_{0.5}Ta_{0.5}O_3$ polymorphs

To study the effect of Ca doping and hydrostatic pressure on distortion mode amplitudes in the lowest-energy rII and AFE phases of PST, we performed the following analysis. We start with pure PST and consider fully relaxed structures of the c, rII, and AFE polymorphs. For a clearer symmetry-mode decomposition, we replace Sc and Ta cations with a single cation type—Zr. All distortions were referenced to the parent 5-atom cubic perovskite cell of $PbZrO_3$, with lattice constants taken from the fully relaxed cubic PST phase.

Using the ISODISTORT tool [12, 13], we identify the dominant symmetry-adapted distortions that transform the ideal cubic reference into the rII and AFE phases. In the rII phase, these distortions are: 1) Γ_4^- — polar displacement of Zr cations with respect to O anions along the $\langle 111 \rangle$ direction; 2) R_5^- — antiphase tilting ($a^-a^-a^-$) of oxygen octahedra.

In the AFE phase, the dominant modes are: 1) Σ_2 — antipolar displacements of Pb cations along $[11\bar{1}0]$, accompanied by deformation of the oxygen octahedra; 2) R_5^- — antiphase $a^-a^-c^0$ oxygen octahedral rotations.

In Supplementary Table 8, we present the parent-cell-normalized amplitudes A_p of these distortion modes. Here:

$$A_p = A_s \sqrt{\frac{V_p}{V_s}}, \qquad (1)$$

where V_p and V_s are the primitive parent and supercell volumes, respectively, and A_s is the supercell-normalized amplitude of the mode (root-summed-squared mode-induced displacements within the primitive supercell).

Next, we repeat the same analysis for 12.5% Ca-doped PST and for pure PST under a hydrostatic pressure of 10 GPa. The results are summarized in Supplemen-

tary Tables 8 and 9.

The analysis reveals that the polar distortion (Γ_4^-) in the rII phase is relatively unaffected by Ca doping but is notably suppressed under hydrostatic pressure. The antipolar displacements of the Pb cations (Σ_2 mode) are strongly reduced by both Ca doping and pressure. In turn, the octahedral tilt amplitude increases in the rII and AFE phases under both conditions.

Supplementary Table 8: Computed distortion mode amplitudes (in Å) for the rII phase of pure PST under 0 GPa and 10 GPa hydrostatic pressure, as well as for 12.5% Ca-doped PST. Γ_4^- is the polar distortion mode, and R_5^- is associated with the antiphase $a^-a^-a^-$ oxygen octahedral tilts.

**Distortion mode**	**PST**	**12.5% Ca-doped PST**	**PST under 10 GPa**
Γ_4^-	0.67126	0.62871	0.34079
R_5^-	0.47156	0.54817	0.55960

Supplementary Table 9: Computed distortion mode amplitudes (in Å) for the antiferroelectric (AFE) phase of pure PST under 0 and 10 GPa hydrostatic pressure, as well as for 12.5% Ca-doped PST. The Σ_2 mode corresponds to antipolar displacements of the A-site cations and distortions of the oxygen octahedra, while the R_5^- mode is associated with antiphase $a^-a^-c^0$ oxygen octahedral tilts.

**Distortion mode**	**PST**	**12.5% Ca-doped PST**	**PST under 10 GPa**
Σ_2	0.31328	0.19755	0.11943
R_5^-	0.46695	0.57780	0.58762

References

[1]N. Novak, F. Weyland, S. Patel, H. Guo, X. Tan, J. Rödel, and J. Koruza, "Interplay of conventional with inverse electrocaloric response in $(\mathrm{Pb,Nb})(\mathrm{Zr,Sn,Ti})\mathrm{O}_3$ antiferroelectric materials", Phys. Rev. B **97**, 094113 (2018).

[2]Z. Xu, D. Viehland, and D. A. Payne, "An incommensurate–commensurate phase transformation in antiferroelectric tin-modified lead zirconate titanate", J. Mater. Res. **10**, 453–460 (1995).

[3]Z. Xu, X. Dai, and D. Viehland, "Incommensuration in la-modified antiferroelectric lead zirconate titanate ceramics", Appl. Phys. Lett. **65**, 3287–3289 (1994).

[4]W. Pan, Q. Zhang, A. Bhalla, and L. E. Cross, "Field-forced antiferroelectric-to-ferroelectric switching in modified lead zirconate titanate stannate ceramics", Journal of the American Ceramic Society **72**, 571–578 (1989).

[5]Z. Liu and B.-X. Xu, "Insight into perovskite antiferroelectric phases: landau theory and phase field study", Scr. Mater. **186**, 136–141 (2020).

[6]X. J. Ding, L. P. Xu, Z. G. Hu, X. F. Chen, G. S. Wang, X. L. Dong, and J. H. Chu, "Phase diagram and incommensurate antiferroelectric structure in ceramics discovered by band-to-band optical transitions", Applied Physics Letters **105**, 131909 (2014).

[7]U. Bismayer, V. Devarajan, and P. Groves, "Hard-mode raman spectroscopy and structural phase transition in the relaxor ferroelectric lead scandium tantalate, pb (sc0. 5ta0. 5) o3", Journal of Physics: Condensed Matter **1**, 6977 (1989).

[8]B. Mihailova, U. Bismayer, B. Güttler, M. Gospodinov, and L. Konstantinov, "Local structure and dynamics in relaxor-ferroelectric $Pb(Sc_{0.5}Nb_{0.5})O_3$ and $Pb(Sc_{0.5}Ta_{0.5})O_3$ single crystals", Journal of Physics: Condensed Matter **14**, 1091 (2002).

[9]J. Petzelt, E. Buixaderas, and A. V. Pronin, "Infrared dielectric response of ordered and disordered ferroelectric Pb($Sc_{1/2}Ta_{1/2}$)O_3 ceramics", Materials Science and Engineering: B **55**, 86–94 (1998).

[10]H. Lu, S. Glinsek, P. Buragohain, E. Defay, J. Iñiguez, and A. Gruverman, "Probing antiferroelectric–ferroelectric phase transitions in $PbZrO_3$ capacitors by piezoresponse force microscopy", Advanced Functional Materials **30**, 2003622 (2020).

[11]D. Chen, C. T. Nelson, X. Zhu, C. R. Serrao, J. D. Clarkson, Z. Wang, Y. Gao, S.-L. Hsu, L. R. Dedon, Z. Chen, et al., "A strain-driven antiferroelectric-to-ferroelectric phase transition in la-doped $BiFeO_3$ thin films on Si", Nano Letters **17**, 5823–5829 (2017).

[12]H. T. Stokes, D. M. Hatch, and B. J. Campbell, *ISODISTORT, ISOTROPY Software Suite*, `http://iso.byu.edu`, Accessed: 2025-08-11.

[13]B. J. Campbell, H. T. Stokes, D. E. Tanner, and D. M. Hatch, "Isodisplace: an internet tool for exploring structural distortions", Journal of Applied Crystallography **39**, 607–614 (2006).
