# Supplementary material for: Tunable electrocaloric effect in lead scandium tantalate through calcium doping
Source: Nat Commun. 2026 Apr 15;17:5250. doi: 10.1038/s41467-026-71911-0 (PMC13260448; doi:10.1038/s41467-026-71911-0)
Supplement: Supplementary file 2 — Transparent Peer Review File [file 41467_2026_71911_MOESM2_ESM.pdf]

nature portfolio

Peer Review File

Tunable Electrocaloric Effect in Lead Scandium Tantalate through Calcium Doping

Corresponding Author: Dr Youri Nouchokgwe

Parts of this Peer Review File have been redacted as indicated to remove third-party material.

This file contains all reviewer reports in order by version, followed by all author rebuttals in order by version.

Attachments originally included by the reviewers as part of their assessment can be found at the end of this file.

Version 0:

Reviewer comments:

Reviewer #1

(Remarks to the Author)
See attached PDF file

Reviewer #2

(Remarks to the Author)
The manuscript "Tunable Electrocaloric Effect in Lead Scandium Tantalate through Calcium Doping" by Nouchokgwe et al. presents a comprehensive and compelling study on tailoring the electrocaloric response of PST via A-site Ca doping. The combination of extensive experimental characterization (calorimetry, electrical measurements, PFM, Raman) with DFT calculations provides strong evidence for the stabilization of an antiferroelectric phase and the resultant tuning of the electrocaloric effect (ECE). The work is of high quality and significance to the field. However, several points require clarification and minor additional analysis to strengthen the manuscript before publication.
1. The phase diagram for PCa2ST (Fig. 3c) predicts an inverse ECE at the AFE-PE transition under low fields, yet this was not experimentally observed. The authors attribute this to the effect being too small. This point warrants a more detailed discussion.
2. The investigation of the inverse ECE in PCa4.6ST is focused on the high-temperature region near the AFE-PE transition. It would be highly informative to measure the EC response at lower temperatures (e.g., down to 230-260 K). This could reveal if a larger inverse ΔT_adiab occurs at lower temperatures, potentially near a putative AFE-FE transition induced by higher electric fields, providing a more complete picture of its caloric portfolio.
3. The EC effect in PCa4.6ST was measured using a thermistor, with a maximum $|\Delta T\_adiab|$ of ~0.7 K. Since this magnitude is within the detection capability of a modern IR camera, it is recommended to perform comparative measurements using the IR camera setup on PCa4.6ST. This would serve as a valuable cross-validation of the two direct measurement techniques used in the manuscript.
4. The thermistor method employs a correction factor of 1.8 to account for thermal losses. The derivation and justification for this specific factor should be explicitly detailed in the Methods section to ensure reproducibility and transparency.
5. The PFM data in Supplementary Note 9 provides good evidence for the AFE state. To further solidify these findings, it is recommended to: Present a more complete set of PFM switching spectroscopy data, including both on-field and off-field measurements for both PST and PCa4.6ST under identical DC voltage conditions. To further enhance the reliability of the microstructural analysis and minimize potential misinterpretation, the authors should address the issue of topography crosstalk (provide topography images in the supplementary data). This is a well-known artifact in PFM where the phase and amplitude signals can be strongly influenced by local surface topography, potentially mimicking or obscuring genuine electromechanical contrast.
6. The quality and clarity of several figures, particularly in the Supplementary Information (e.g., Supplementary Figure 13 on DSC measurements), should be improved. Please ensure all figures have consistently high resolution, clear axis labels, distinct data points, and legible legends to meet the journal's publication standards

Reviewer #3

(Remarks to the Author)
This manuscript reports a comprehensive study on highly ordered A-site calcium-doped lead scandium tantalate ($PbSc_{0.5}Ta_{0.5}O_3$, PST) ceramics, in which the phase transition behavior is modified to enable a tunable electrocaloric response across a broad temperature range. By introducing calcium dopants, the authors successfully stabilized an intermediate antiferroelectric (AFE) phase between the ferroelectric and paraelectric states for compositions with Ca ≥ 2%, allowing for the observation of both conventional electrocaloric effects ($\triangle T_{adiab}$ ≈ 4.6 K in pure PST) and inverse electrocaloric responses in heavily doped samples. The study systematically correlates macroscopic thermal and electrical measurements with microscopic piezoresponse force microscopy (PFM) and density functional theory (DFT) calculations to elucidate that the AFE stabilization arises from combined local lattice distortions and thermal effects rather than simple volume reduction. The work presents a generalizable strategy for expanding the operational window of PST-based regenerators to sub-ambient temperatures (down to 263 K), offering scientific significance and promising potential for cascaded electrocaloric cooling devices. However, there are several lacks in the manuscript that need to be solved or answered in its current state.

1. The explanation for the mechanism by which Ca doping stabilizes the AFE phase requires further elucidation. Currently, the attribution relies primarily on DFT energy comparisons and qualitative descriptions of local structural relaxations. Given the minute energy difference between the FE and AFE phases, zero-temperature calculations based on a single configuration are insufficient to fully support the experimental observations. It is recommended to provide statistical results from multiple Ca configurations, along with analyses of local Ca-O and Pb-O bond lengths or local volume variations, to more clearly demonstrate how Ca doping stabilizes the AFE phase in real materials.
2. The performance of PST is highly dependent on the degree of Sc/Ta ordering. While the authors achieved high ordering through extended annealing, further explanation is needed regarding whether Ca doping influences the kinetics of B-site cation ordering. Although XRD data indicate a high degree of order, a brief discussion would be beneficial: if the degree of ordering were lower, would the Ca-induced AFE phase still be stable?
3. For phase-change cooling materials, particularly those involving AFE-FE transitions associated with significant volume changes, cycling stability is a critical issue. Could the authors provide relevant experimental data or an explanation to demonstrate that the AFE-FE phase transition remains stable after repeated cycling?
4. The FE to AFE transition in PCa2ST is not discernible in the zero-field DSC curves but manifests as a distinct peak under iso-field conditions. This discrepancy requires further explanation. For instance, does this transition possess weak first-order or diffuse characteristics, or is it influenced by intragranular stress and energy barriers?
5. The quantitative analysis of the electrocaloric effect should be strengthened. While the maximum $\triangle T_{adiab}$ values are presented, the lack of comparison regarding specific entropy change, thermal conductivity, sample dimensions (mass/thickness), and measurement techniques makes objective comparison within the system difficult. Furthermore, since a thermistor was used for PCa4.6ST while an IR camera was used for other samples, it is recommended to include a discussion on the consistency between these two thermometry methods, or provide a calibration comparison in the Supplementary Information.
6. The manuscript reports an inverse electrocaloric effect in the 4.6% Ca-doped sample with a $\triangle T_{adiab}$ ≈ -0.7 K. While this represents an interesting physical phenomenon, the magnitude is relatively small. Given that the authors propose the potential of these materials for cascaded devices in the Conclusion, it is recommended to provide a more objective evaluation in the Discussion section regarding the actual efficiency contribution of this "inverse electrocaloric effect" in a practical refrigeration cycle. Alternatively, the authors should explicitly clarify whether the primary significance of this specific composition lies in elucidating the scientific mechanism rather than in direct high-efficiency cooling performance.

Version 1:

Reviewer comments:

Reviewer #1

(Remarks to the Author)
The authors have satisfactorily addressed all my concerns. I therefore recommend acceptance in its present form.

Reviewer #2

(Remarks to the Author)
The authors have addressed all reviewer comments well. The new experiments—low-field ECE measurements on PCa2ST, PFM switching spectroscopy with topography analysis, and IR camera measurements on PCa4.6ST—have improved the manuscript. All concerns have been resolved. The manuscript is now suitable for publication in Nature Communications.

Reviewer #3

(Remarks to the Author)
The authors have satisfactorily addressed all comments.

Open Access This Peer Review File is licensed under a Creative Commons Attribution 4.0 International License, which permits use, sharing, adaptation, distribution and reproduction in any medium or format, as long as you give appropriate credit to the original author(s) and the source, provide a link to the Creative Commons license, and indicate if changes were made.
In cases where reviewers are anonymous, credit should be given to 'Anonymous Referee' and the source.
The images or other third party material in this Peer Review File are included in the article's Creative Commons license, unless indicated otherwise in a credit line to the material. If material is not included in the article's Creative Commons license and your intended use is not permitted by statutory regulation or exceeds the permitted use, you will need to obtain permission directly from the copyright holder.
To view a copy of this license, visit https://creativecommons.org/licenses/by/4.0/

Responses to the referees:

We thank the reviewers for their time and constructive evaluation of our manuscript. We have carefully addressed all reviewer comments and provide a detailed, point-by-point response.

The revised manuscript has been substantially improved and now includes four new experiments. The main revisions are summarized as follows:

- We **performed new electrocaloric characterization** measurements using an infrared camera to **confirm the inverse electrocaloric effect in 2% Ca-doped PST**. A thorough analysis of these results is now presented in the revised manuscript.
- We **reassessed and confirmed the electrocaloric effect of 4.6% Ca-doped PST** using a more reliable technique based on infrared thermography, replacing the initial measurements performed with a thermistor.
- We **provide a complete set of Piezoresponse Force Microscopy (PFM) switching spectroscopy data**, including both on-field and off-field measurements. In addition, **we address the issue of topography crosstalk** by presenting new Atomic Force Microscopy (AFM) topography images alongside corresponding PFM phase images.
- **New SEM (scanning electron microscopy) imaging and EDS (energy-dispersive spectroscopy) mapping** of the 1% Ca-doped PST sample were performed to discuss and confirm the compositional homogeneity of the material.
- Finally, **we added** a section entitled **"First-principles calculations"** to the manuscript, describing our extensive density functional theory (DFT) results.

Reviewer #1 (Remarks to the Author):

This manuscript presents an interesting approach to broadening the electrocaloric operating window of PST through Ca doping. The authors show that Ca doping stabilizes an antiferroelectric phase and enables a tunable electrocaloric effect spanning roughly 263–353 K, with peak ΔT_{adiab} values of ~2 K under high electric fields. The combined use of DSC, dielectric spectroscopy, Raman scattering, PFM, and DFT calculations provides a rich dataset. Nevertheless, several points require further clarification or expansion before the manuscript can be considered for publication.

Answer: We appreciate the reviewer for the positive comments on our manuscript, and we have revised the manuscript accordingly based on your detailed comments.

Major comments

1. Given the diffuse dielectric and P–E responses observed in the Ca-doped samples, it would be helpful for the authors to discuss whether increasing Ca content could lead to local coexistence of AFE and FE regions near the phase boundaries. For PCa4.6ST, the dielectric anomaly observed around 15 °C (Supplementary Fig. 11d) is not clearly explained in the manuscript. It would be important for the authors to clarify whether this feature is associated with an intrinsic phase transition, with a possible incommensurate–commensurate transition commonly reported in antiferroelectrics?

Answer: We thank the reviewer for pointing this out and helping us improve our manuscript.

As observed in the dielectric measurements, calcium doping broadens (smears out) the phase transition. This behaviour cannot be attributed to Sc/Ta disorder in the Ca-doped samples, as we verified that they remain highly ordered (see Table 1). Therefore, we assume that the effect arises from the local coexistence of antiferroelectric (AFE) and ferroelectric (FE) regions. The diffuse nature of the transition is thus attributed to calcium inducing competition between AFE and FE phases. It is worth noting that similar diffuse phase transitions induced by doping have been reported in other Pb-based perovskites exhibiting antiferroelectricity [1-2].

Indeed, the dielectric measurements carried out in the antiferroelectric PCa4.6ST shows an anomaly at 15°C. Such an anomaly has been reported [3-6] in lead-based antiferroelectrics. So, we can say that similar anomaly has been observed in other Pb-based AFEs and it was attributed to incommensurate AFE to a commensurate AFE, which suggests that in our case the same occurs.

[1] N. Novak, F. Weyland, S. Patel, H. Guo, X. Tan, J. Rodel, and J. Koruza, "Interplay of conventional with inverse electrocaloric response in (Pb, Nb)(Zr, Sn, Ti)O3 antiferroelectric materials", Phys. Rev. B 97, 094113 (2018).
[2] W. Pan, Q. Zhang, A. Bhalla, and L. E. Cross, "Field-forced antiferroelectric-to-ferroelectric switching in modified lead zirconate titanate stannate ceramics", Journal of the American Ceramic Society 72, 571–578 (1989).

[3] Xu, Z., Viehland, D. & Payne, D. A. An incommensurate–commensurate phase transformation in antiferroelectric tin-modified lead zirconate titanate. *J. Mater. Res.* 10, 453–460 (1995).
[4] Xu, Z., Dai, X. & Viehland, D. Incommensuration in La-modified antiferroelectric lead zirconate titanate ceramics. *Appl. Phys. Lett.* 65, 3287–3289 (1994).
[5] Liu, Z. & Xu, B.-X. Insight into perovskite antiferroelectric phases: Landau theory and phase field study. *Scr. Mater.* 186, 136–141 (2020).
[6] Ding, X. J. *et al.* Phase diagram and incommensurate antiferroelectric structure in $(Pb_{1-1.5x}La_x)(Zr_{0.42}Sn_{0.40}Ti_{0.18})O_3$ ceramics discovered by band-to-band optical transitions. *Appl. Phys. Lett.* 105, 131909 (2014).

Modification:

To clarify the diffusiveness of the dielectric constant with Ca-doping and the anomaly observed in PCa4ST, we added the following text and references in Supplementary Note 6 as follows:

In this Supplementary Note, we present dielectric measurements collected at 1 kHz for Ca-doped PST samples during both heating and cooling, performed at a rate of 1 K/min. The extracted transition temperatures are consistent with those obtained from DSC measurements. More specifically, for PCa2ST, we observe a shoulder around −17 °C, which coincides with the field-induced FE–AFE transition identified by in situ field-dependent DSC (Figures 1c and 3c). In addition, dielectric measurements of the antiferroelectric PCa4.6ST sample reveal an anomaly at approximately 15 °C. Such anomalies are commonly observed in lead-based antiferroelectric materials [1–4] and are attributed to a transition from an incommensurate AFE phase to a commensurate AFE phase. Finally, the dielectric constant exhibits an increasingly diffuse transition with rising Ca content. This diffuse behaviour is attributed to calcium doping, which induces the coexistence of competing AFE and FE phases, a phenomenon reported [5-6] in doped Pb-based perovskites.

Revised references:

[1] N. Novak, F. Weyland, S. Patel, H. Guo, X. Tan, J. Rodel, and J. Koruza, “Interplay of conventional with inverse electrocaloric response in (Pb, Nb)(Zr, Sn, Ti)O3 antiferroelectric materials”, Phys. Rev. B 97, 094113 (2018).
[2] W. Pan, Q. Zhang, A. Bhalla, and L. E. Cross, “Field-forced antiferroelectric-to-ferroelectric switching in modified lead zirconate titanate stannate ceramics”, Journal of the American Ceramic Society 72, 571–578 (1989).
[3] Xu, Z., Viehland, D. & Payne, D. A. An incommensurate–commensurate phase transformation in antiferroelectric tin-modified lead zirconate titanate. *J. Mater. Res.* 10, 453–460 (1995).
[4] Xu, Z., Dai, X. & Viehland, D. Incommensuration in La-modified antiferroelectric lead zirconate titanate ceramics. *Appl. Phys. Lett.* 65, 3287–3289 (1994).
[5] Liu, Z. & Xu, B.-X. Insight into perovskite antiferroelectric phases: Landau theory and phase field study. *Scr. Mater.* 186, 136–141 (2020).
[6] Ding, X. J. *et al.* Phase diagram and incommensurate antiferroelectric structure in $(Pb_{1-1.5x}La_x)(Zr_{0.42}Sn_{0.40}Ti_{0.18})O_3$ ceramics discovered by band-to-band optical transitions. *Appl. Phys. Lett.* 105, 131909 (2014).

2. The Tc shows a non-monotonic trend with Ca doping, reaching a minimum at 1% Ca. What is the mechanism that causes Tc to decrease initially and then increase again with higher Ca content?

Answer: Thank you for giving us the opportunity to clarify this point. T_c shows a non-monotonic trend with Ca doping because the nature of the transition changes across the composition range.

For pure PST and 1% Ca-doped PST, T_c corresponds to the paraelectric (PE)–ferroelectric (FE) transition. In this regime, Ca substitution destabilizes the FE phase, leading to a decrease in the transition temperature from 297 K (0% Ca) to 282 K (1% Ca).

At higher Ca concentrations, the system undergoes a different transition. The antiferroelectric (AFE) phase emerges and becomes the stable low-temperature phase. In this case, T_c corresponds to the PE–AFE transition, and the associated transition temperature increases with further Ca doping, reaching 317 K for 4.6% Ca. This evolution is summarized in the phase diagram shown in Figure 3e of the main text and in the figure below.

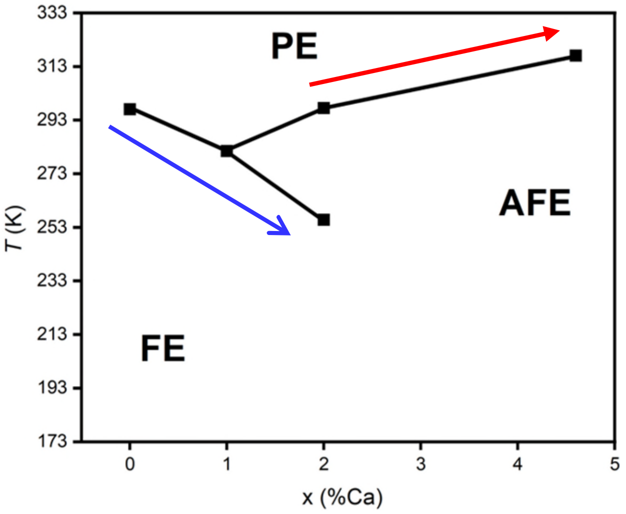

Figure: Transition temperature as a function of calcium content. The blue arrow indicates the shift of the FE peak to lower temperatures at low calcium content (≤2%). The red arrow indicates the shift of the AFE peak to higher temperatures at higher content (≥2%). FE peak vanishes at higher calcium content (>2%).

Modification:

For clarity, we added the following text in the manuscript to explain the non-monotonic behaviour of the transition temperature with calcium content. (pg. 13)

Finally, Figure 3e presents the zero-field phase diagram of highly ordered calcium-doped PST, where the transition temperatures T are shown as a function of calcium content. The non-monotonic behavior of T arises from the fact that calcium stabilizes the antiferroelectric (AFE) phase while destabilizing the ferroelectric (FE) phase. At low calcium concentrations (≤ 2% Ca), the FE phase is destabilized, leading to a monotonic decrease in the transition temperature. Specifically, the FE phase is shifted from 297 K (0% Ca) to 282 K (1% Ca) and further to 256 K (2% Ca). At higher calcium contents, the AFE phase appears and is shifted to higher temperatures. In this regime, the AFE-to-PE peak increases from 297 K (2% Ca) to 317 K (4.6% Ca).

3. The explanation invoking Ca-rich and Ca-poor regions at low doping levels is speculative, as no experimental evidence or literature reference is provided to support this claim. The authors should either substantiate this statement with appropriate data (e.g., compositional mapping) or clearly indicate it as a hypothesis.

Answer: We thank the reviewer for this comment. In the initial version of the manuscript, we hypothesized that the slight increase in ECE around 298 K in 1% Ca-doped PST sample could be related to the presence of Ca-rich and Ca-poor regions. To verify this hypothesis, we performed SEM (scanning electron microscopy) imaging and EDS (energy-dispersive spectroscopy) mapping on the 1% Ca-doped PST sample. After examining a large number of regions across the sample surface, we detected only a single Ca-rich region, indicating a high degree of compositional homogeneity. Thus, we conclude that the observed increase in ECE cannot be attributed to the sample inhomogeneity. Therefore, we have removed our initial explanation from the manuscript.

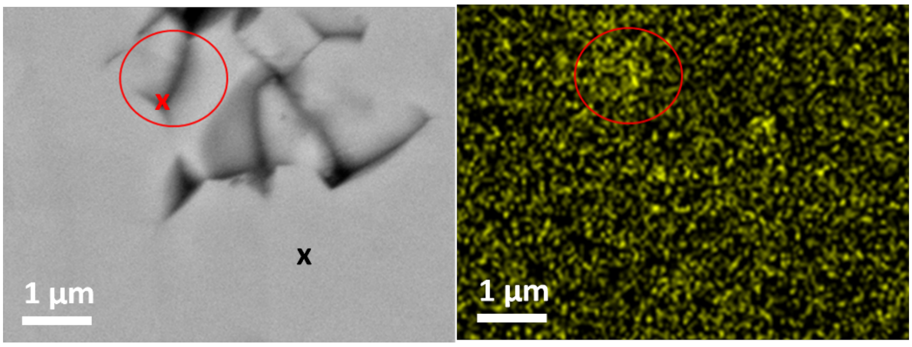

Figure: SEM (on the left) and EDS (on the right) of 1%Ca-doped PST. The areas of analysis are marked by black (matrix) and red (Ca-rich area) crosses in the SEM image.

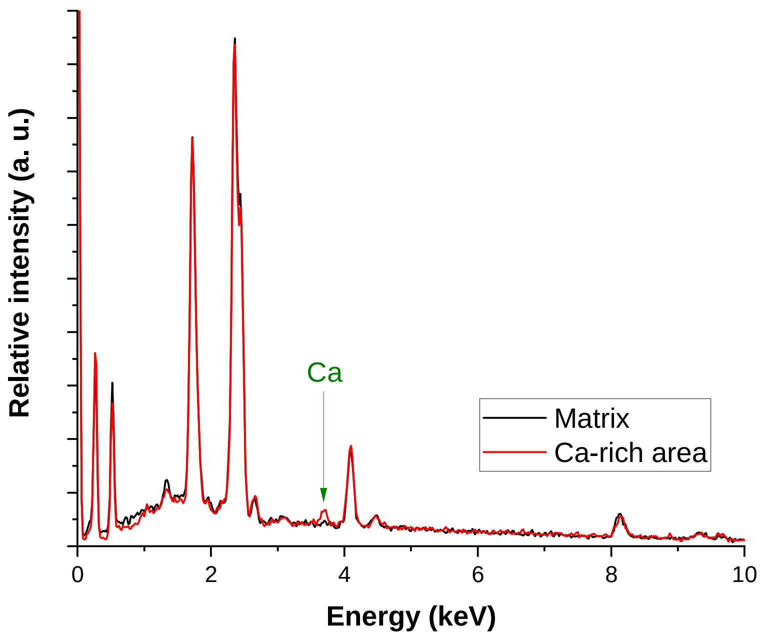

Modification:

- We removed from the main text the explanation invoking Ca rich and Ca-poor regions at low doping levels.

4. *The cooling curves in Fig. 2 exhibit unusually large noise and distorted features, which cast doubt on the physical meaning of these data.*

Answer: We thank the reviewer for this comment. The distorted features observed in Figure 2c on the cooling curves at 23 kV/cm and 17 kV/cm are artifacts caused by a spike during the isofield measurements, which led to a sudden jump in the DSC signal. These features do not have any physical significance.

5. It should be noted that the apparent broadening of the operating temperature range depends strongly on the chosen ΔT_{adiab} threshold. If one requires $\Delta T_{adiab} > 2$ K, Ca doping does not significantly extend the usable span; the broadening becomes evident only when a lower threshold (e.g., >1 K) is considered. To more rigorously support the claim of an expanded operating window, a systematic comparison of ΔT_{adiab} with key literature PST compositions would be helpful.

Answer: We thank the reviewer for the comment. A maximum adiabatic temperature change of 1.8 K in PST, observed between 293 K and 313 K, has been employed in one of our state-of-the-art electrocaloric cooling devices [J. Li et al. Science 382, 801–805 (2023).] Based on this benchmark, we decided to consider $\Delta T_{adiab} \geq 1.8$ K as a threshold to discuss the operating temperature range. As highlighted in the figure below, PST operates between 295 K and 353 K with a 1.8 K temperature change (see red vertical bars in figure below). With Ca-doping of 2% we shift the operating temperature range to lower temperatures. We measure ΔT_{adiab} =1.8 K between 263 K and 323 K (see blue vertical bars below), this is 32K lower than in PST.

For a fair comparison, we compared our results to recent work [Yin, R., Hou, Y., Lv, X. *et al. J. Adv. Ceram.* 14, 9221088 (2025)] on PST bulk. The authors could cover a temperature range between 288K and 318K with 1.8K of adiabatic temperature change. 2%Ca doping enables going down to 263K.

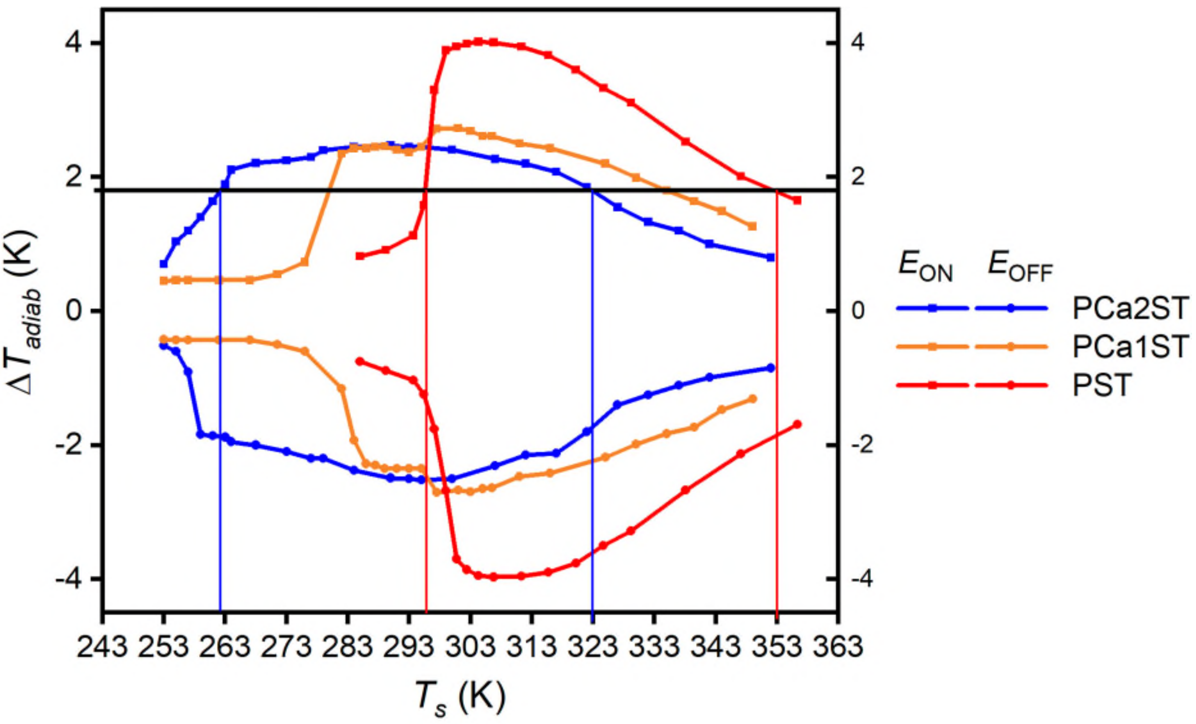

Figure: Adiabatic temperature change of PCaxST compounds as a function of starting temperature. The black horizontal line sets the threshold of 1.8 K. The blue and red vertical bars limit the operating temperature of respectively PCa2ST and PST.

Modification:
Based on the reviewers' comment we have revised the following sentences:

Figure 5 illustrates how the conventional electrocaloric (EC) effect of calcium-doped PST (undoped PST, PCa1ST, and PCa2ST) can be exploited to extend the operating temperature range of PST toward lower temperatures. A maximum adiabatic temperature change (ΔT_{adiab}) of 1.8 K in PST, observed between 293 K and 313 K, has been implemented in a state-of-the-art electrocaloric cooling device [4]. Based on this benchmark, we adopt $\Delta T_{adiab} \geq 1.8$ K as a threshold criterion to define the practical operating temperature range. Undoped PST operates between 295 K and 353 K with $\Delta T_{adiab} \geq 1.8$ K. Upon Ca doping, the operating window shifts toward lower temperatures: PCa1ST (1% Ca) operates between 280 K and 336 K, while PCa2ST (2% Ca) covers the range from 263 K to 323 K. In an electrocaloric regenerator, combining PCa2ST with PST would enable coverage of a broad temperature range from 263 K to 353 K while maintaining $\Delta T_{adiab} \geq 1.8$ K.
For comparison, the electrocaloric effect reported for less ordered bulk PST [32] reaches approximately 1.8 K over a temperature interval of 288–318 K. The 4.6% Ca-doped composition is not considered here, as its small negative temperature change under applied electric field would counteract the positive EC response of the other compositions.

In addition, the device-oriented statements should be moderated unless supported by realistic modelling of multilayer architectures or cycling performance.

Answer: We thank the reviewer for the comment and for giving us the opportunity to improve our manuscript. It is well known in the literature [L. Shebanovs et al., Ferroelectrics 273:1 (2002); Nair et al. Nature 575 (2019)] that applied electric field enables expanding the operating temperature range of electrocaloric materials. In addition, multilayer

capacitors can sustain higher electric fields than bulk ceramics due to their reduced dielectric layer thickness and improved breakdown strength. This allows to apply stronger electric fields and to expand the temperature range even further.

To illustrate this, we compare the electrocaloric effect of PST bulk ceramic [Nouchokgwe at al., Nat.Comm 12(1) (2021)] and PST multilayer capacitor [Nouchokgwe et al., Scripta Materiala 219 (2022)] measured under different applied electric fields as reported in our previous works, see figure below. In both cases, the temperature range increases with increased electric field. However, the accessible temperature range is larger for PST MLC since it can withstand larger electric fields than the bulk sample.

Based on this, we propose that the fabrication of multilayer capacitors using calcium-doped PST could enable the application of higher electric fields compared to bulk ceramics and potentially expand the material's operational temperature range.

[Figure Redacted]

Figure: Electrocaloric effect of PST bulk ceramics (left) and PST multilayers capacitors (right). These figures were taken from Nouchokgwe at al., Nat.Comm (2021) (left) and Nouchokgwe et al., Scripta Materiala (2022) (right).

Modification:
We corrected our statement as follows:

In future work, the fabrication of multilayer capacitors [30] using calcium-doped PST could enable the application of higher electric fields and potentially further expand the material's operational temperature range as demonstrated for pure PST in Ref. 7.

6. The manuscript states that the inverse EC effect at low fields is too small to be detected. Could the authors clarify whether highly precise thermistor measurements were attempted to resolve this effect?

Answer: We thank the reviewer for pointing this out, it helps us improve our manuscript. Indeed, the phase diagram of 2%Ca-PST suggests that an inverse electrocaloric effect should occur near the AFE-PE transition at low electric fields (below 30 kV/cm). Now, we have investigated this region (indicated by the blue rectangle in Supplementary Figure 14 and in the figure below) in greater detail using an infrared camera.

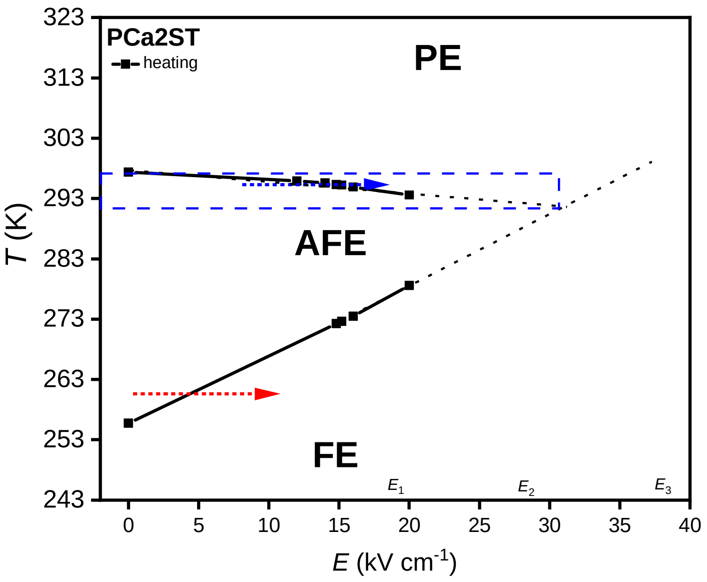

Figure: Phase diagram of PCa2ST. Transition temperature as a function of electric field in 2%Ca-doped PST. FE, AFE and PE refer to the ferroelectric, antiferroelectric and paraelectric phases, respectively. The red arrow describes the conventional electrocaloric effect from the AFE to the FE. The blue arrow describes the inverse effect. The blue rectangle is the region at the vicinity of the AFE-to-PE transition where an inverse electrocaloric is expected.

- At 292 K, within the antiferroelectric (AFE) phase, we applied a series of electric fields (19, 23, 27, 28, 30, 34, and 38 kV/cm). As shown in the Figure below, an inverse electrocaloric effect is observed for electric fields below 30 kV/cm as the material is moved from a low entropy phase (AFE) to a higher entropy phase (PE). The magnitude of the inverse electrocaloric temperature change is -0.1K at 19 kV/cm and increases to a maximum of -0.25 K at 23 kV/cm. With further increasing electric field, the magnitude of the inverse EC effect decreases and vanishes at 30 kV/cm. For electric fields exceeding 30 kV/cm, the EC response becomes conventional. This behaviour arises because high electric fields drive the material into a ferroelectric phase, which possesses a lower entropy than the AFE phase.

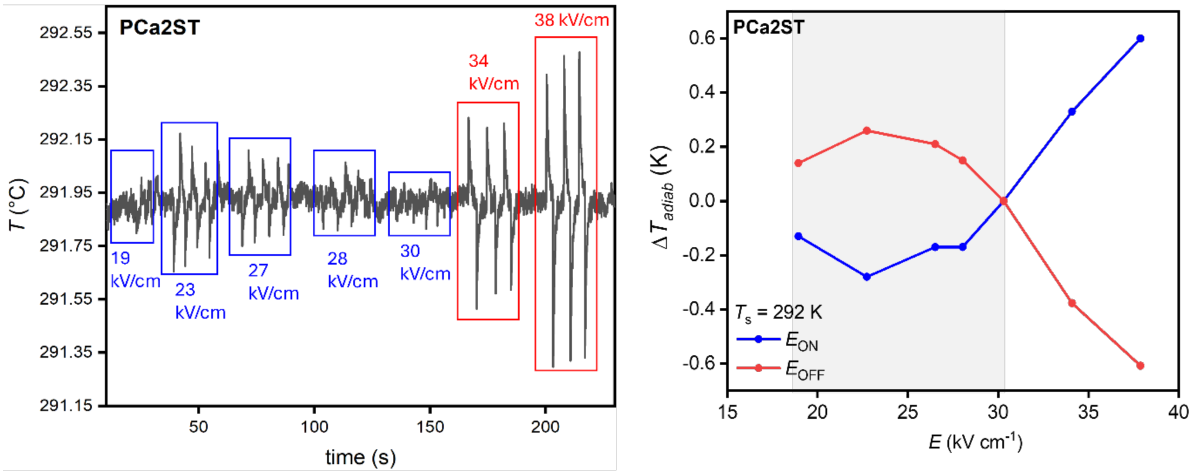

Figure: Direct electrocaloric measurements of PCa2ST at low electric fields and around the AFE-to-PE transition. On the left, the temperature change under different electric bias is shown. On the right, the adiabatic temperature change is shown as a function of an applied electric field. The grey area highlights the region where an inverse electrocaloric effect was observed.

- We collected direct adiabatic temperature change at various electric fields and different starting temperatures, and the results are presented in the Figure below. Below the AFE-PE transition temperature, an inverse electrocaloric effect is observed. At 293K, we measure a maximum inverse electrocaloric effect of -0.25K. This peak shift towards lower temperatures with increasing field, as observed in the phase diagram (see Figure 3c). This response becomes conventional upon crossing the AFE-PE transition temperature and when the applied electric field exceeds 30 kV/cm. In this regime, the material is in the paraelectric phase, and the application of a strong electric field immediately induces a transition to the ferroelectric phase, thereby resulting in a conventional electrocaloric effect. For the electrocaloric effect at higher electric fields refer to Figure 4c of the main text.

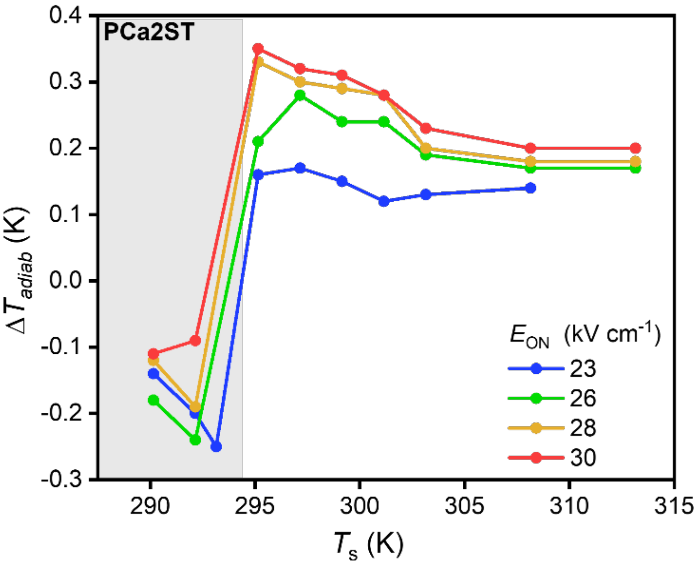

Figure: Electrocaloric effect of PCa2ST at different starting temperatures upon application of different electric fields. The grey area shows the region where an inverse electrocaloric effect was observed.

- The following figure describes the electrocaloric effect of PCa2ST upon application and removal of an electric field.

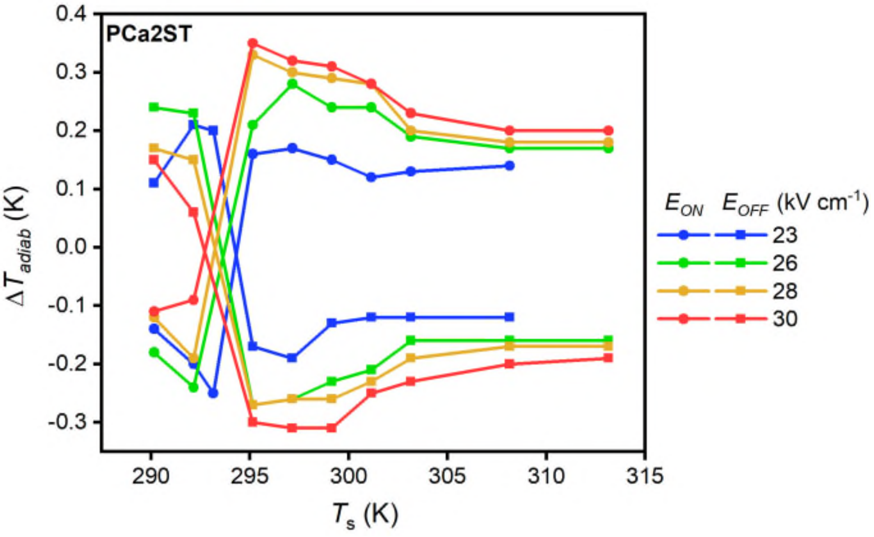

Figure: Electrocaloric effect of PCa2ST as function of starting temperature, at different electric fields.

To better understand the tunable electrocaloric effect in PCaST, three scenarios can be described from the phase diagram, corresponding to three different starting temperatures (T_1, T_2, and T_3) and three applied electric fields (E_1, E_2, and E_3). See Figure below.

- The vertical dashed black lines define the three electric fields (E_1 = 18, E_2 = 27, and E_3 = 39 kV cm^{-1}).
- The horizontal short dot arrows indicate the phase transition for different electric fields applied at a given starting temperature.
- The blue and red colors represent the inverse and conventional electrocaloric effects, respectively.

First case. At **T_1 = 260 K**, close to the AFE–FE transition, applying E_1, E_2, or E_3 drives the material into the stable FE phase. A conventional electrocaloric effect is expected, as the AFE phase possesses a higher entropy than the FE phase.

Second case. At **T_2 = 294 K**, within the AFE region, applying E_1 or E_2 drives the material from the AFE phase to the PE phase, resulting in an inverse electrocaloric effect since the AFE phase has a lower entropy than the PE phase. When the higher electric field E_3 is applied, the material transitions to the FE phase, producing a conventional electrocaloric effect because the AFE phase has a higher entropy than the FE phase.

Third case. At **T_3 = 297 K**, applying E_1 or E_2 keeps the material in the PE phase, resulting in a conventional electrocaloric effect. At the higher electric field E_3, the material transitions into the FE phase, also exhibiting a conventional electrocaloric effect.

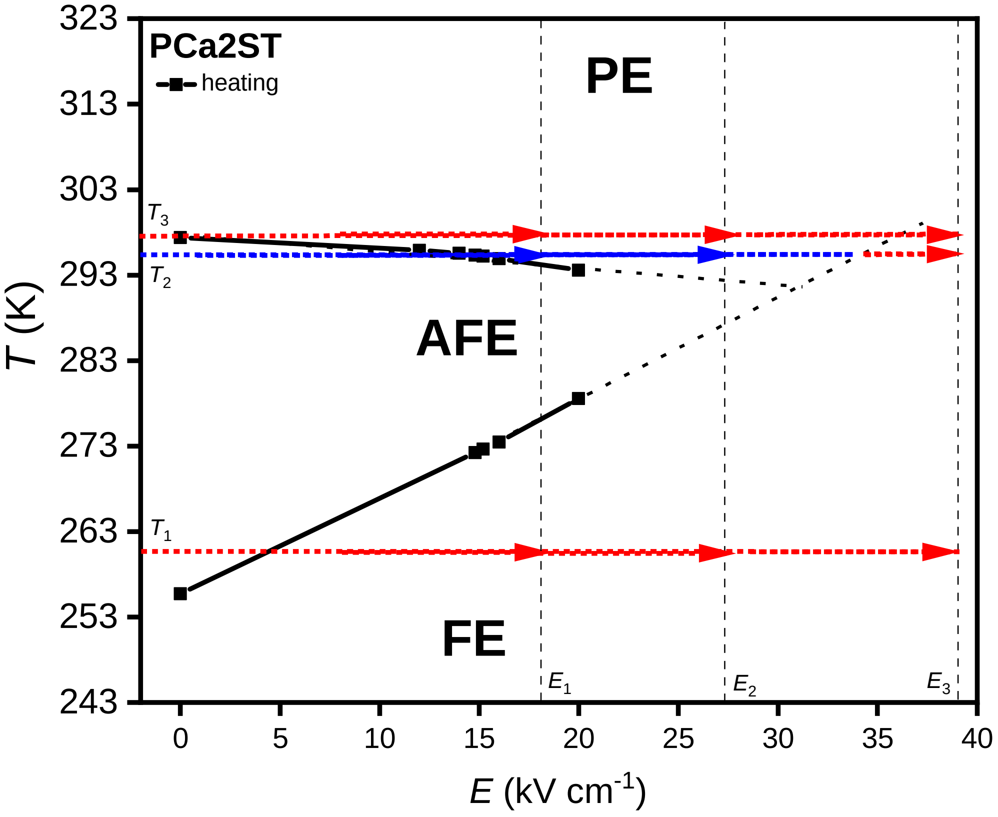

Figure: Phase diagram of PCa2ST. Transition temperature T as a function electric field E. FE, AFE and PE are respectively the ferroelectric, antiferroelectric and paraelectric phases. T_1, T_2, and T_3 denotes three starting temperatures from which three applied electric fields E_1, E_2, and E_3 are applied. The vertical dashed black lines define the three electric fields (E_1 = 18, E_2 = 27, and E_3 = 39 kV cm^{-1}). The horizontal short dot arrows indicate the phase transition for different electric fields applied at a given starting temperature. The blue and red colors represent the inverse and conventional electrocaloric effects, respectively.

Modification:

- We added a new section into the Supplementary Information: Electrocaloric effect in PCa2ST. The answer provided above was added to this section.

- We modified the Figure 4 of the manuscript. We added the new data on the direct electrocaloric measurements of PCa2ST at low electric fields.

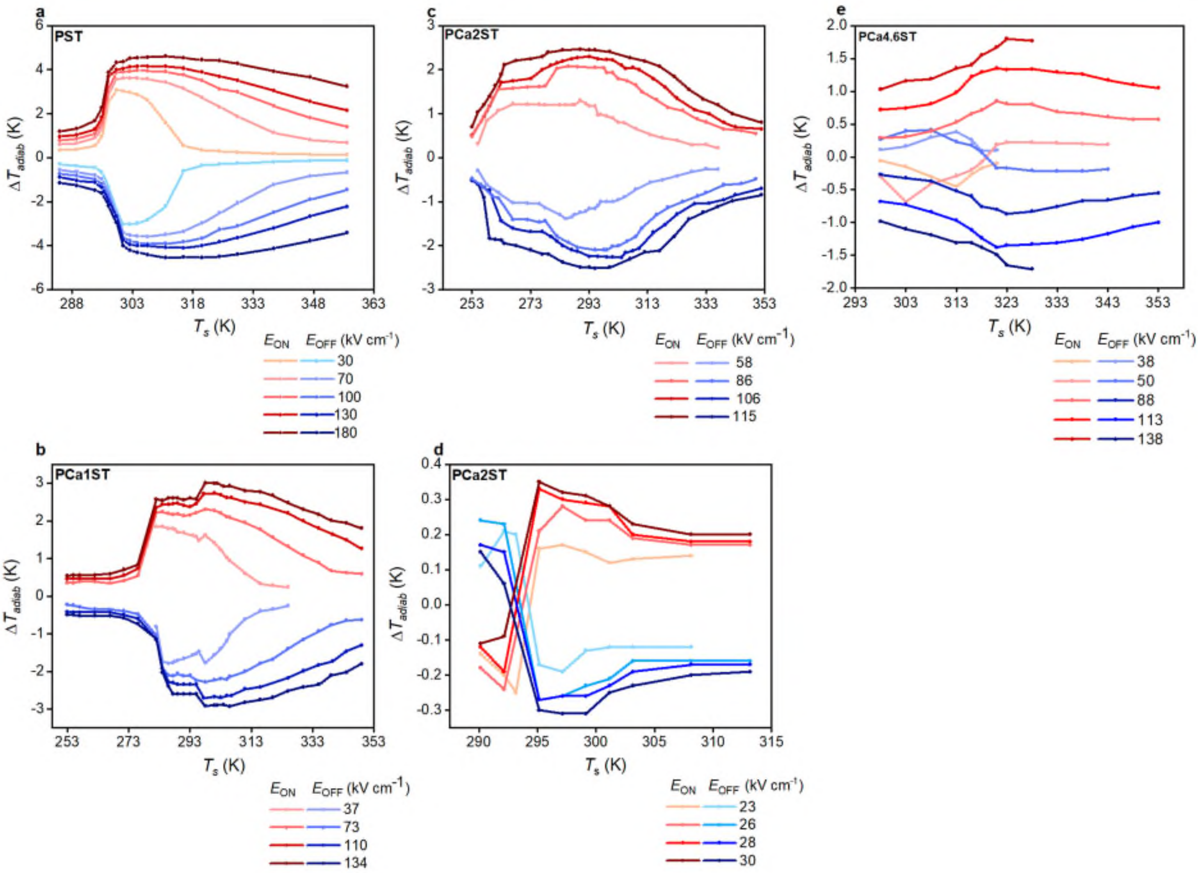

Figure: Electrocaloric effect of calcium-doped PST. Adiabatic temperature change, ΔT_{adiab}, of the different compositions measured at various starting temperatures (T_s) and electric fields (E). A conventional EC effect is observed in PST (a) and PCa1ST (b). In PCa2ST, a conventional EC effect is obtained at high electric fields (> 30 kV/cm) (c), whereas an inverse EC effect is observed near the AFE–PE transition at low electric fields (≤ 30 kV/cm) (d). PCa4.6ST exhibits an inverse electrocaloric effect (e).

- We added the following text in the main text, in the section "Electrocaloric effect of calcium-doped PST".

  We further examine the electrocaloric (EC) response of PCa2ST in detail. PCa2ST exhibits both conventional (Figure 4c) and inverse (Figure 4d) EC effects, depending on the applied electric field and temperature.

  A conventional EC effect is observed upon application of high electric fields (> 30 kV/cm) (see Figure 4c). At 115 kV cm^{-1}, the maximum adiabatic temperature changes (ΔT_{adiab}) reach 2.0 K and 2.5 K at the FE–to–AFE (264 K) and AFE–to–PE (297 K) phase transitions, respectively. The conventional EC response at the FE–to–AFE transition originates from the positive slope of dT/dE (Figure 3c). For the AFE–to–PE transition, when the electric field exceeds 30 kV cm^{-1}, the material is driven into the ferroelectric (FE) phase. Since the FE phase has a lower entropy than the antiferroelectric (AFE) phase, the field-induced transition results in a conventional EC effect.

In contrast, the inverse electrocaloric effect in PCa2ST (Figure 4d) appears near the AFE–to–PE phase transition at low electric fields (≤ 30 kV/cm). Below 295 K, a persistent but low-magnitude inverse EC response is observed. The maximum inverse adiabatic temperature change reaches −0.25 K, with the peak shifting toward lower temperatures as the electric field increases. This behavior is consistent with the PCa2ST phase diagram, which shows a negative dT/dE slope at the AFE–to–PE transition. Moreover, the magnitude of the inverse EC effect decreases with increasing electric field and transitions to a conventional response above the triple point at 30 kV cm^{-1} (see Supplementary Note 7). Above 295 K, the EC response becomes entirely conventional, as the material is stabilized in the paraelectric (PE) phase.

- We added the following text in the main text, in the section "Introduction".

Using an infrared camera, we measured the electrocaloric response under applied electric fields in Ca-doped PST compounds. Conventional EC effect was observed for Ca contents ≤ 2%, while inverse EC effect appeared for Ca contents ≥ 2%. For the ferroelectric compounds PST and 1%Ca-PST, maximum adiabatic temperature changes (ΔT_{adiab}) of 4.6 K at 313 K and 2.6 K at 285 K, respectively, were obtained at their FE–to–PE phase transitions. In 2%Ca-doped PST, which exhibits two successive phase transitions (FE–to–AFE and AFE–to–PE), both conventional and inverse EC effects were measured. At 115 kV cm^{-1}, maximum ΔT_{adiab} values of 2.0 K and 2.5 K were obtained at the FE–to–AFE and AFE–to–PE transitions, respectively. At low applied electric fields (< 30 kV cm^{-1}), a negligible inverse EC effect of −0.25 K was observed at the AFE–to–PE transition.

The antiferroelectric 4.6%Ca-PST compound exhibits a pronounced inverse EC effect at its AFE–to–PE transition, reaching a maximum ΔT_{adiab} of −0.6 K at 303 K. With increasing electric field and temperature, this inverse effect progressively transforms into a conventional EC effect, with a maximum ΔT_{adiab} of 1.8 K at 323 K.

In addition, for a fair comparison of measurement reliability, the infrared camera and thermistor methods should ideally be applied to the same sample under identical experimental conditions.

Answer: We thank the reviewer for the constructive comment. As suggested, we characterized PCa4.6ST using an infrared camera to measure the adiabatic temperature change during the application and removal of the electric field. The values obtained are in very good agreement with those measured using the thermistor. The corresponding data are presented in the figure below.

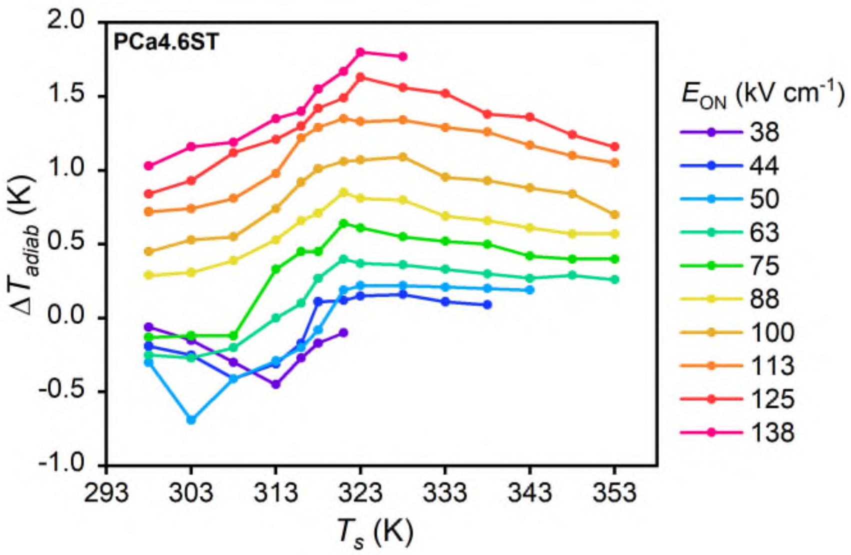

Figure: Electrocaloric effect of PCa4.6ST using an IR camera. The data were collected at different electric fields and starting temperatures. The data shown here are upon application of an electric field.

At the lowest applied electric field of 38 kV/cm, we observe an inverse EC effect of -0.5 K. With increasing electric field, the peak shifts toward lower temperatures, reaching a maximum value of -0.6 K at 303 K under 50 kV/cm. This behavior is consistent with the phase diagram of PCa4.6ST. At higher temperatures and electric fields, the inverse electrocaloric effect turns into a conventional one. A maximum conventional EC temperature change of 1.8 K is measured at 323 K under an electric field of 138 kV/cm. Note that here, in contrast to the thermistor characterization, we were able to apply a higher electric field of 138kV/cm close to the material's breakdown field.

Modification:

- We added the direct characterization with IR camera to the manuscript. Figure 4 was updated. For clarity, the adiabatic temperature change for five different electric fields was added in the manuscript.

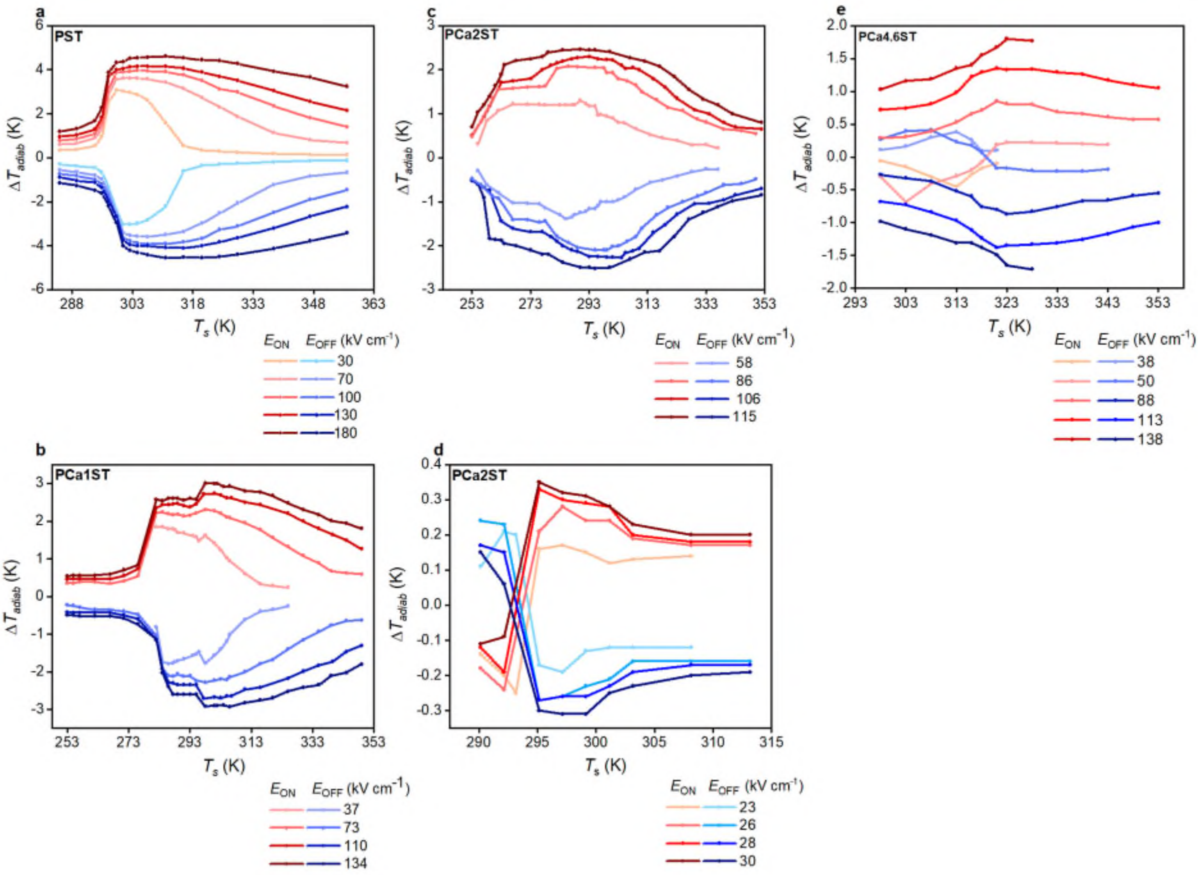

Figure: Electrocaloric effect of calcium-doped PST. Adiabatic temperature change, ΔT_{adiab}, of the different compositions measured at various starting temperatures (T_s) and electric fields (E). A conventional EC effect is observed in PST (a) and PCa1ST (b). In PCa2ST, a conventional EC effect is obtained at high electric fields (> 30 kV/cm) (c), whereas an inverse EC effect is observed near the AFE–PE transition at low electric fields (≤ 30 kV/cm) (d). PCa4.6ST exhibits an inverse electrocaloric effect (e).

- In Supplementary Information 12, we present a thorough analysis of the adiabatic temperature change of PCa4.6ST at all the electric fields studied.

- We added the following text in the main text, in the section “Electrocaloric effect of calcium-doped PST”.

Finally, we investigate the electrocaloric (EC) effect in the antiferroelectric material PCa4.6ST. As shown in Figure 4e, an inverse electrocaloric effect is observed in the vicinity of the AFE–to–PE transition. At 313 K under an applied electric field of 38 kV/cm, an inverse EC temperature change of -0.5 K is measured. With increasing electric field, the EC peak shifts toward lower temperatures, reaching a maximum inverse temperature change of -0.6 K at 303 K under 50 kV/cm.

At higher temperatures and electric fields, the EC response transitions to a conventional effect. A maximum conventional EC temperature change of 1.8 K is recorded at 323 K under 138 kV/cm (Figure 4e). As indicated in Figure 3d, an inverse EC response is expected in this region because dT/dE is negative. Moreover, the P–E loops of PCa4.6ST (Figure 1d and Supplementary Figure~10) reveal an increase in polarization with temperature, corresponding to a positive ∂P/∂T and therefore a negative ΔTadiab, consistent with the Maxwell relation [5]. At sufficiently high electric fields

and temperatures, the paraelectric phase becomes energetically favored, resulting in a positive (conventional) EC effect.

- We added the following text in the main text, in the section "Introduction".
  The antiferroelectric 4.6%Ca-PST compound exhibits a pronounced inverse EC effect at its AFE–to–PE transition, reaching a maximum ΔT_{adiab} of −0.6 K at 303 K. With increasing electric field and temperature, this inverse effect progressively transforms into a conventional EC effect, with a maximum ΔT_{adiab} of 1.8 K at 323 K.

7. Supplementary Note 11 provides extensive DFT data (energies, phonons, pressure dependence, distortion-mode amplitudes). However, the main text compresses these results too aggressively. For clarity, the authors should explicitly state the rII–AFE energy differences (Supplementary Fig. 21a), summarize how these energies evolve with Ca doping (Supplementary Tables 3–6), and clearly indicate that the stabilization of the AFE phase cannot be explained by volume changes alone, consistent with the DFT results.

Answer: We thank the referee for this comment. In the revised version of the manuscript, we added a section describing our main theoretical results.

Modification:

- We added the following section "First-principles calculations" to the main text.

First-principles calculations.

In order to gain further understanding of the antiferroelectric behavior observed experimentally, we study the stability of different structural polymorphs of pure and Ca-doped PST using density functional theory (see Methods for the computational details). We start by considering an 80-atom supercell of pure PST and restrict ourselves to four structural polymorphs (see Supplementary Figure 4): (i) paraelectric cubic $Fm\bar{3}m$ (#225, we denote it further as "c"); (ii) rhombohedral *R3m* (#160) which has a polar distortion along a <111> direction (we will denote it as "rI" phase); (iii) rhombohedral *R3* (#146) which has both a polar distortion along a <111> direction and an antiphase oxygen octahedra tilt pattern $a^-a^-a^-$ in Glazer notation about the same axis ("rII" phase); (iv) monoclinic $P2_1/c$ (#14) which is derived from the antiferroelectric *Pbam* phase of $PbZrO_3$ (PZO) by replacing Zr atoms with Sc and Ta cations in the perfectly rocksalt-ordered way; it includes an $a^-a^-c^0$ octahedra tilt pattern alongside antipolar displacements of the Pb cations ("AFE" phase). We fully optimize the crystal structures of these polymorphs and compute their energies using DFT. The optimized lattice parameters are listed in Supplementary Table 3, and the computed energies are shown in Supplementary Figure 21a. We find that the rII phase has the lowest energy; however, the AFE polymorph is just 2.7 meV per A cation higher in energy. A similar result has been reported by Paściak *et al.* for $PbSc_{0.5}Nb_{0.5}O_3$ [1]. Additionally, for PZO, the AFE *Pbam* and FE *R3c* phases (where the latter is analogous to the *R3* phase but with only one type of B cation) were found to be very close in energy, with AFE *Pbam* being the ground state [2]. Note that the addition of zero-point energy

contributions does not alter the hierarchy of these polymorphs (see Supplementary Note 11 and Supplementary Table 2 for details).

Next, we extend this analysis to Ca-doped PST with Ca concentrations of 6.25% and 12.5%. For this, we start from the same four polymorphs of pure PST described above (structural polymorhps c, rI, rII and AFE in our 80 atom supercell) and replace one or two Pb atoms, respectively, by Ca atoms as illustrated in Supplementary Figures 5 and 6. We fully optimize all considered structures and compute their energies using DFT. As shown in Supplementary Figure 21a, within the studied range of concentrations, Ca doping does not affect the relative stability of considered polymorphs compared to pure PST. rII remains the lowest energy configuration and the AFE phase is higher in energy by a few meV per A cation. For 6.25% Ca-doped PST, the c and rI configurations relax to lower symmetry phases. Namely, the c phase develops the octahedral tilts of the pattern similar to that of the AFE phase, while rI relaxes to the rII phase. In Supplementary Figure 21a we show the energies of these polymorphs with triangular symbols. In turn, the interpolated energy values between 0% and 12.5% Ca concentrations are shown with lines. Structurally, 12.5% Ca doping leads to a volume reduction of approximately 1% in the rII phase and 1.3% in the AFE phase (see Supplementary Table 3 and Supplementary Figure 24). It also increases the amplitude of oxygen octahedral tilts in both rII (by ~14%) and AFE (by ~19%) phases, while reducing the polar distortion in the rII phase (by ~6%) and the antipolar Pb displacements in the AFE phase (by ~37%).

At the next step, we investigate how the relative stability of rII and AFE polymorphs is affected by other factors, such as hydrostatic pressure and increasing temperature. Since the Ca concentration does not have a significant effect on the stability of these phases in our simulations, we restrict the analysis to the case of pure PST. First, we study how the relative stability of the rII and AFE phases of PST evolves with increasing hydrostatic pressure. For that, we fully optimize the structures of both phases for the pressure values in the range from 0 GPa to 10 GPa (see Supplementary Figure 25 and Supplementary Table 4 for the structural data) and compute the energies of the relaxed structures. As shown in Supplementary Figure 22, the rII phase is favored at low pressures, while the AFE phase becomes more stable above ~3 GPa. At this pressure, the cell volume is reduced by ~3% in the rII phase and by ~2% in the AFE phase compared to the zero-pressure structure. In addition, the distortion mode amplitudes are affected as follows: the oxygen octahedral tilts are increased in both rII (by 5.3%) and AFE (by 7.2%) phases, polar distortion in the rII phase is reduced by 14.8%, and antipolar displacements of Pb cations in the AFE phase are reduced by 18.6%.

Finally, temperature effects are examined by computing the free energies F (within the harmonic approximation as described in Methods) of the rII and AFE phases of PST in the range between 0 K and 500 K. The results Supplementary Figure 21b show that the ferroelectric rII phase is favored at low temperatures, and the AFE phase becomes more stable above 170 K. Note that, in our experiments, we did not observe the transition from FE to AFE phase in pure PST. However, in PCa2ST, this transition occurs at approximately 263 K.

Overall, our first-principles analysis reveals that ferroelectric (rII) and antiferroelectric (AFE) polymorphs of PST are nearly degenerate in energy (at T=0 K), with energy differences of only a few meV per A-site cation, both in pure and Ca-doped systems. Ca substitution up to 12.5% does not invert the energetic hierarchy, although it induces local structural changes, including enhanced oxygen octahedral tilts and reduced polar (in rII phase) and antipolar (in AFE phase) distortions. In contrast, hydrostatic pressure and finite-temperature effects are found to modify the relative stability of the rII and AFE phases, with pressure above ~3 GPa or temperatures above ~170 K favoring the AFE phase in pure PST. Taken together, these results indicate that the experimentally observed stabilization of the AFE phase in Ca-doped PST likely arises from the combination of having near-degenerate FE and AFE phases, Ca-induced local structural relaxations, and thermal effects, which collectively control the delicate balance between competing instabilities and enable antiferroelectricity.

[1] M. Paściak, T. R. Welberry, and J. Hlinka, Phase Transitions, 89, 777 (2016).
[2] J. Íñiguez, M. Stengel, S. Prosandeev, and L. Bellaiche, Phys. Rev. B 90, 220103R (2014).

8. Several figures in the main text appear to be drawn too roughly, which affects their readability. In particular, the axis labels in Figures 1–4 are too small and should be enlarged to improve clarity.

Answer: We thank the reviewer for the comment. We have updated and improve the quality of all the figures in the manuscript. Please, find attached along with this report the individual figures in pdf.

9. The claim that the FE–AFE–PE phase sequence differs from $PbZrO_3$-based systems is not accurate. This sequence is actually common in $PbZrO_3$-derived antiferroelectrics (see early work by Wuyi Pan and colleagues). The authors should revise this statement and cite appropriate literature.

Answer: Thank you for constructive feedback. Indeed, Pan et al. observed a transition phase sequence from FE-to-AFE-to-PE in $Pb_{0.99}La_{0.02}(Zr_{0.66},Sn_{0.25},Ti_{0.09})O_3$. By replacing La with Nb, Novak et al. also reported the same phase sequence.

Modification:
The following text was added to the discussion as well as the reference suggested by the reviewer:

An antiferroelectric phase between the ferroelectric and paraelectric phases as observed in PCa2ST, has also been reported in other lead-based perovskite materials [Tan et al., PRL, 105(25), 255702]. The archetypal AFE material, $PbZrO_3$ (short form -PZO) [Tagantsev et al., Nat Commun. 4, 2229 (2013)], when doped on the A-site with Nb [Novak et al., PRB 97 094113] or La [Pan et al., J. Am. Ceram. Soc., 72 [4] 571-78 (1989)] and on the B-site with Sn and Ti, exhibits a sequence of phase transitions from FE to AFE to PE, which contrasts with the more typical phase sequence, AFE to FE to PE, observed for example in pure PZO [Vales-Castro et al. PRB 103, 054112 (2021)]. In PZO, an electric field induces a stable FE phase between the AFE and PE states, and the temperature range of this FE phase broadens with increasing electric field [Vales-Castro et al. PRB

103, 054112 (2021)]. In contrast, in PCa2ST, the electric field stabilizes the FE phase at lower temperatures while progressively suppressing the AFE region as the field strength increases (see Figure 3c).

Minor comments

1. In Supplementary Figure 1, the scale bars are difficult to read. Please clearly indicate the scale bar values (e.g., add the length in the caption or enlarge the labels).

Answer: We thank the reviewer for spotting this.

Modification:
As suggested, we included in the caption, the length of the scale bar.

Supplementary Figure 1: **Scanning electron miscrocope (SEM) Images of calcium-doped PST**. The SEM images of PST (a, b), PCa1ST (c,d), PCa2ST(e,f), and PCa4.6ST(g,h). The length of the scale bar is 1um.

2. In Supplementary Fig. 13, two red points appear on the right side of the zero-field heating curve, but their meaning is not explained.

Answer: We thank the reviewer for their comment. We have updated the Supplementary Figure 13.

3. "PST" should be defined upon first use in the abstract.

Answer: We thank the reviewer for the suggestion. 'PST' has been defined in the abstract as requested by the reviewer.

Modification:
In the abstract we defined PST.
State-of-the-art electrocaloric cooling prototypes rely on the conventional electrocaloric effect of ferroelectric lead scandium tantalate $PbSc_{0.5}Ta_{0.5}O_3$ (PST), which peaks near room temperature.

4. In Supplementary Fig. 12c, the loss tangent during heating shows two abrupt jumps at low temperatures that are not seen in the cooling curve. These features look like measurement artifacts and the authors should clarify their origin.

Answer: We thank the reviewer for mentioning this. Indeed, these two points are artifacts. The plot has been updated.

5. In Supplementary Fig. 19c, two red arrows are shown on the PFM phase loop, but their meaning is not explained in either the figure caption or the text.

Answer: We thank the reviewer for spotting this. The arrows were indicating the four maxima measured on PCa4.6ST. Following the reviewer's comment and reviewer 2's comment, the figure has been revised (see Supplementary Figure 24).

6. *The references are not formatted according to the journal's guidelines, and several citations (e.g., Ref. 16) are used inappropriately. Please revise the reference list and ensure accurate citation throughout the manuscript.*

Answer: We thank the reviewer for the comment. We have revised the references according to the journal's guidelines and ensure accurate citation.

Reviewer #2 (Remarks to the Author):

The manuscript "Tunable Electrocaloric Effect in Lead Scandium Tantalate through Calcium Doping" by Nouchokgwe et al. presents a comprehensive and compelling study on tailoring the electrocaloric response of PST via A-site Ca doping. The combination of extensive experimental characterization (calorimetry, electrical measurements, PFM, Raman) with DFT calculations provides strong evidence for the stabilization of an antiferroelectric phase and the resultant tuning of the electrocaloric effect (ECE). The work is of high quality and significance to the field. However, several points require clarification and minor additional analysis to strengthen the manuscript before publication.

Answer: We would like to thank the reviewer for the positive comments on our manuscript, and we have revised the manuscript accordingly based on your detailed comments.

1. The phase diagram for PCa2ST (Fig. 3c) predicts an inverse ECE at the AFE-PE transition under low fields, yet this was not experimentally observed. The authors attribute this to the effect being too small. This point warrants a more detailed discussion.

Answer: We thank the reviewer for pointing this out, it helps us improve our manuscript. Indeed, the phase diagram of 2%Ca-PST suggests that an inverse electrocaloric effect should occur near the AFE-PE transition at low electric fields (below 30 kV/cm). Now, we have investigated this region (indicated by the blue rectangle in Supplementary Figure 14 and in the figure below) in greater detail using an infrared camera.

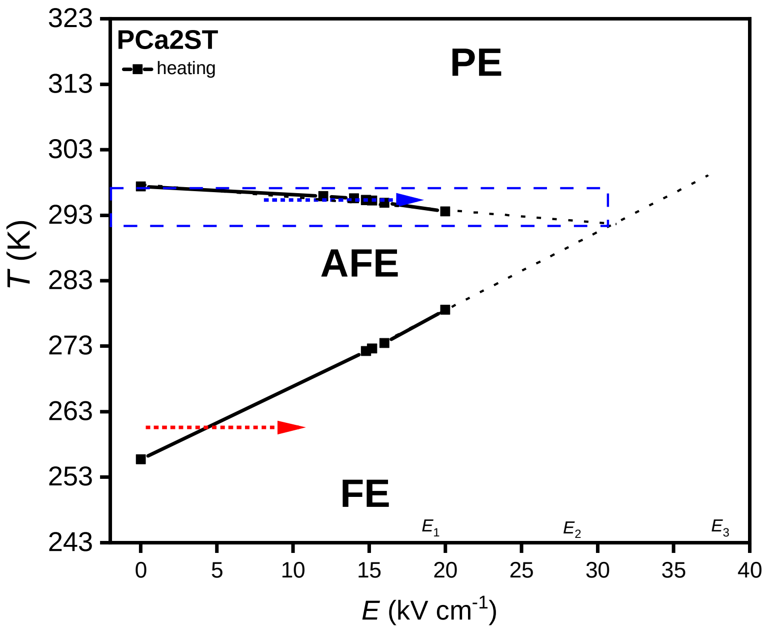

Figure: Phase diagram of PCa2ST. Transition temperature as a function of electric field in 2%Ca-doped PST. FE, AFE and PE refer to the ferroelectric, antiferroelectric and paraelectric phases, respectively. The red arrow describes the conventional electrocaloric effect from the AFE to the FE. The blue arrow describes the inverse effect. The blue rectangle is the region at the vicinity of the AFE-to-PE transition where an inverse electrocaloric is expected.

- At 292 K, within the antiferroelectric (AFE) phase, we applied a series of electric fields (19, 23, 27, 28, 30, 34, and 38 kV/cm). As shown in the Figure below, an inverse electrocaloric effect is observed for electric fields below 30 kV/cm as the material is moved from a low entropy phase (AFE) to a higher entropy phase (PE). The magnitude of the inverse electrocaloric temperature change is -0.1K at 19 kV/cm and increases to a maximum of -0.25 K at 23 kV/cm. With further increasing electric field, the magnitude of the inverse EC effect decreases and vanishes at 30 kV/cm. For electric fields exceeding 30 kV/cm, the EC response becomes conventional. This behaviour arises because high electric fields drive the material into a ferroelectric phase, which possesses a lower entropy than the AFE phase.

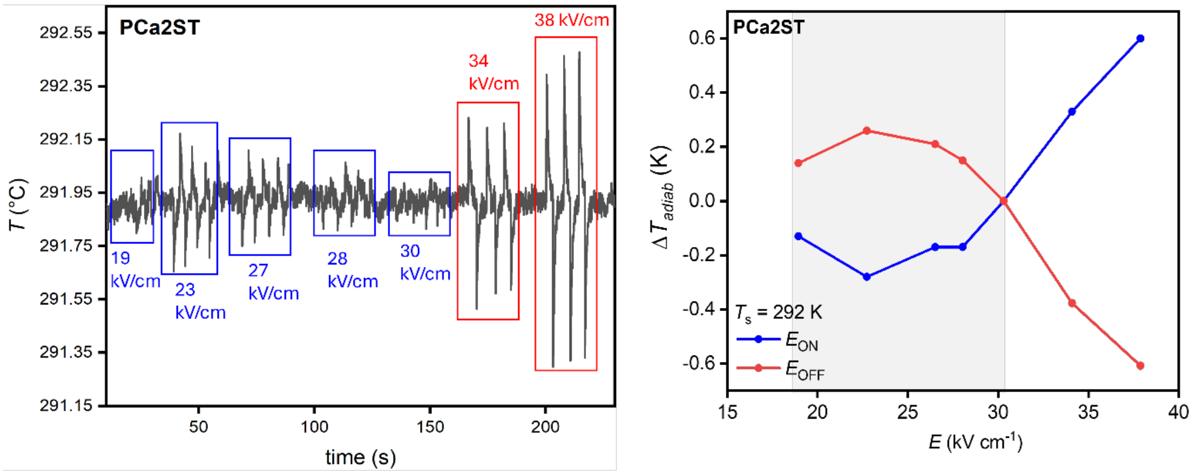

Figure: Direct electrocaloric measurements of PCa2ST at low electric fields and around the AFE-to-PE transition. On the left, the temperature change under different electric bias is shown. On the right, the adiabatic temperature change is shown as a function of an applied electric field. The grey area highlights the region where an inverse electrocaloric effect was observed.

- We collected direct adiabatic temperature change at various electric fields and different starting temperatures, and the results are presented in the Figure below. Below the AFE-PE transition temperature, an inverse electrocaloric effect is observed. At 293K, we measure a maximum inverse electrocaloric effect of -0.25K. This peak shift towards lower temperatures with increasing field, as observed in the phase diagram (see Figure 3c). This response becomes conventional upon crossing the AFE-PE transition temperature and when the applied electric field exceeds 30 kV/cm. In this regime, the material is in the paraelectric phase, and the application of a strong electric field immediately induces a transition to the ferroelectric phase, thereby resulting in a conventional electrocaloric effect. For the electrocaloric effect at higher electric fields refer to Figure 4c of the main text.

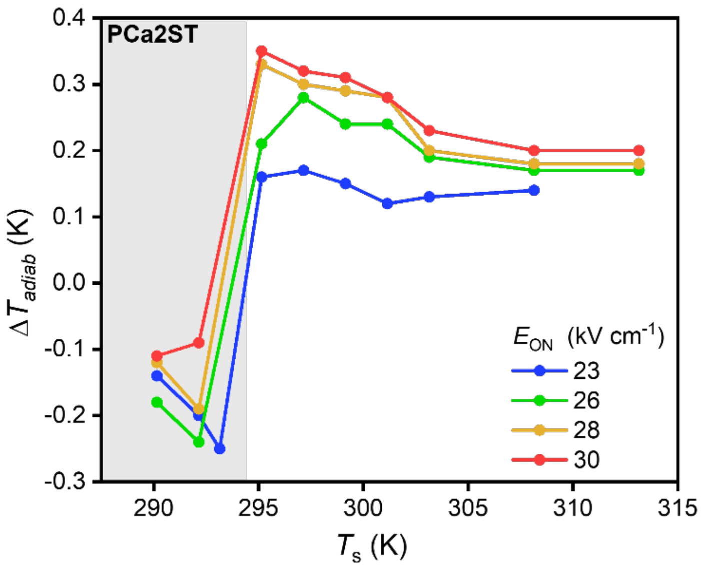

Figure: Electrocaloric effect of PCa2ST at different starting temperatures upon application of different electric fields. The grey area shows the region where an inverse electrocaloric effect was observed.

- The following figure describes the electrocaloric effect of PCa2ST upon application and removal of an electric field.

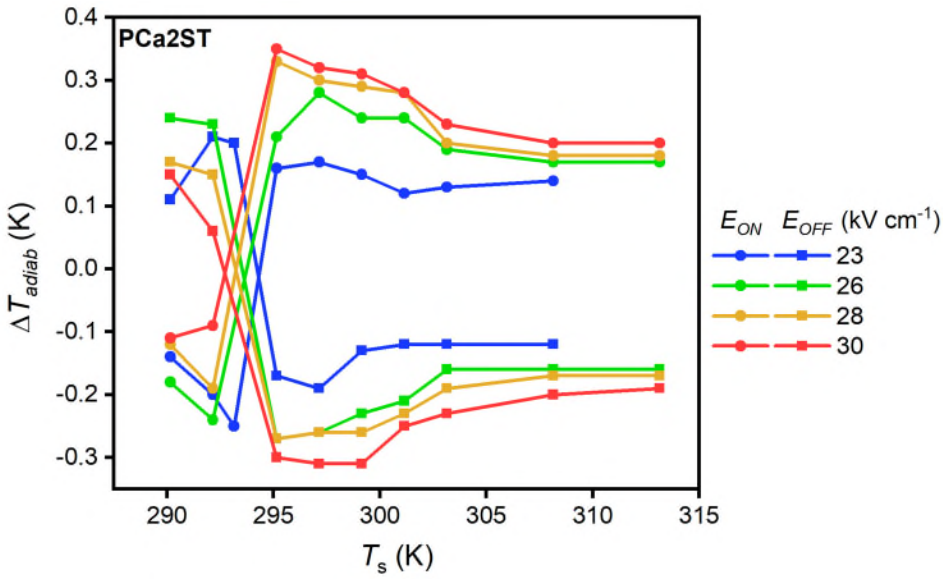

Figure: Electrocaloric effect of PCa2ST as function of starting temperature, at different electric fields.

To better understand the tunable electrocaloric effect in PCaST, three scenarios can be described from the phase diagram, corresponding to three different starting temperatures (T_1, T_2, and T_3) and three applied electric fields (E_1, E_2, and E_3). See Figure below.

- The vertical dashed black lines define the three electric fields (E_1 = 18, E_2 = 27, and E_3 = 39 kV cm^{-1}).
- The horizontal short dot arrows indicate the phase transition for different electric fields applied at a given starting temperature.

- The blue and red colors represent the inverse and conventional electrocaloric effects, respectively.

First case. At **T_1 = 260 K**, close to the AFE–FE transition, applying E_1, E_2, or E_3 drives the material into the stable FE phase. A conventional electrocaloric effect is expected, as the AFE phase possesses a higher entropy than the FE phase.

Second case. At **T_2 = 294 K**, within the AFE region, applying E_1 or E_2 drives the material from the AFE phase to the PE phase, resulting in an inverse electrocaloric effect since the AFE phase has a lower entropy than the PE phase. When the higher electric field E_3 is applied, the material transitions to the FE phase, producing a conventional electrocaloric effect because the AFE phase has a higher entropy than the FE phase.

Third case. At **T_3 = 297 K**, applying E_1 or E_2 keeps the material in the PE phase, resulting in a conventional electrocaloric effect. At the higher electric field E_3, the material transitions into the FE phase, also exhibiting a conventional electrocaloric effect.

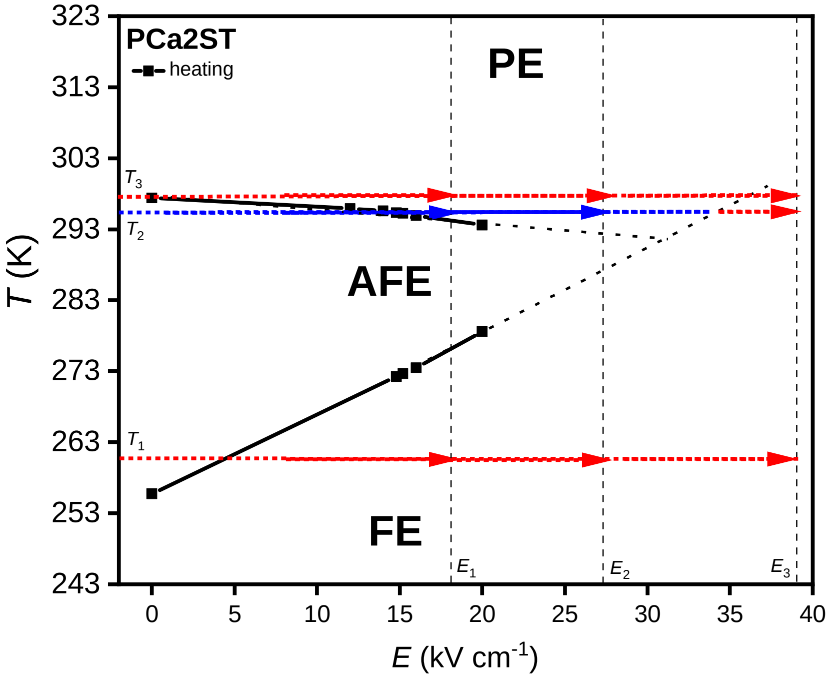

Figure: Phase diagram of PCa2ST. Transition temperature T as a function electric field E. FE, AFE and PE are respectively the ferroelectric, antiferroelectric and paraelectric phases. T_1, T_2, and T_3 denotes three starting temperatures from which three applied electric fields E_1, E_2, and E_3 are applied. The vertical dashed black lines define the three electric fields (E_1 = 18, E_2 = 27, and E_3 = 39 kV cm^{-1}). The horizontal short dot arrows indicate the phase transition for different electric fields applied at a given starting temperature. The blue and red colors represent the inverse and conventional electrocaloric effects, respectively.

Modification:

- We added a new section into the Supplementary Information: Electrocaloric effect in PCa2ST. The answer provided above was added to this section.

- We modified the Figure 4 of the manuscript. We added the new data on the direct electrocaloric measurements of PCa2ST at low electric fields.

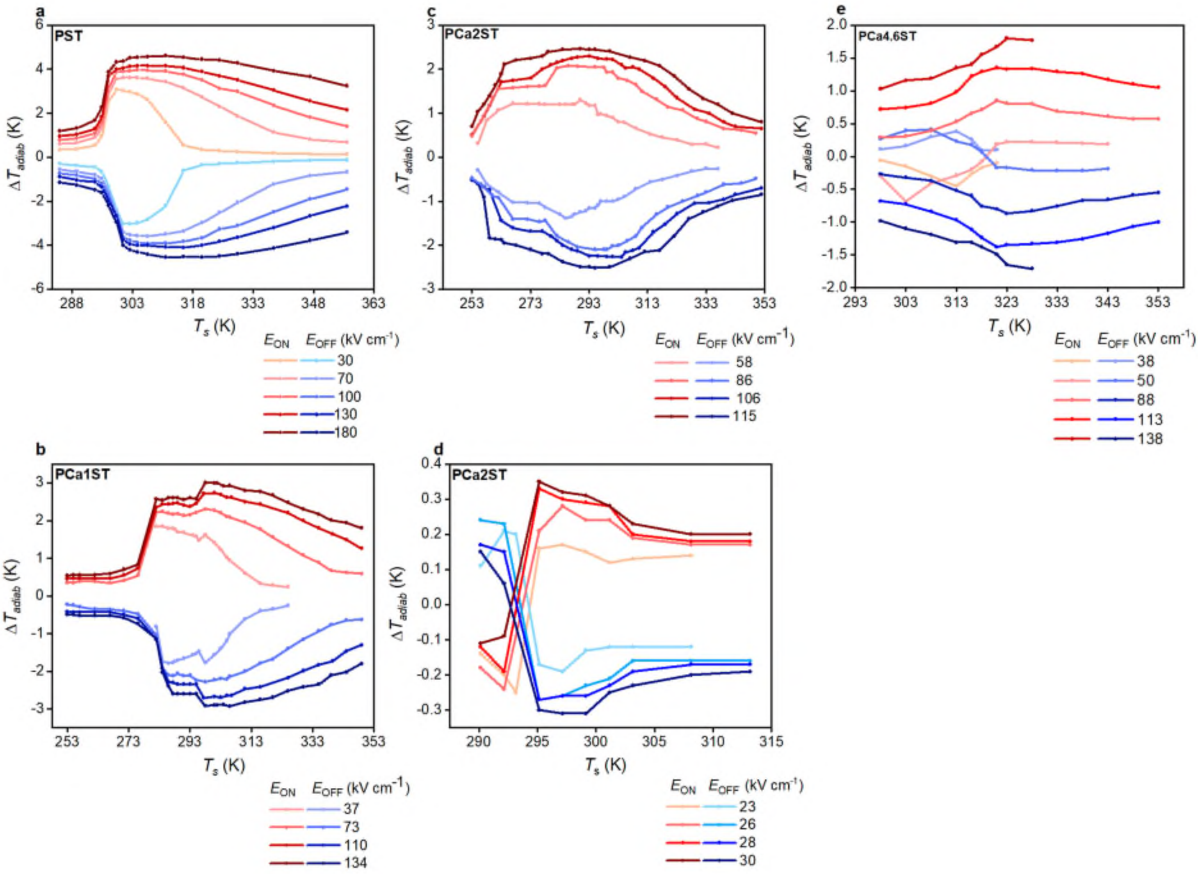

Figure: Electrocaloric effect of calcium-doped PST. Adiabatic temperature change, ΔT_{adiab}, of the different compositions measured at various starting temperatures (T_s) and electric fields (E). A conventional EC effect is observed in PST (a) and PCa1ST (b). In PCa2ST, a conventional EC effect is obtained at high electric fields (> 30 kV/cm) (c), whereas an inverse EC effect is observed near the AFE–PE transition at low electric fields (≤ 30 kV/cm) (d). PCa4.6ST exhibits an inverse electrocaloric effect (e).

- We added the following text in the main text, in the section "Electrocaloric effect of calcium-doped PST".

We further examine the electrocaloric (EC) response of PCa2ST in detail. PCa2ST exhibits both conventional (Figure 4c) and inverse (Figure 4d) EC effects, depending on the applied electric field and temperature.

A conventional EC effect is observed upon application of high electric fields (> 30 kV/cm) (see Figure 4c). At 115 kV cm^{-1}, the maximum adiabatic temperature changes (ΔT_{adiab}) reach 2.0 K and 2.5 K at the FE–to–AFE (264 K) and AFE–to–PE (297 K) phase transitions, respectively. The conventional EC response at the FE–to–AFE transition originates from the positive slope of dT/dE (Figure 3c). For the AFE–to–PE transition, when the electric field exceeds 30 kV cm^{-1}, the material is driven into the ferroelectric (FE) phase. Since the FE phase has a lower entropy

than the antiferroelectric (AFE) phase, the field-induced transition results in a conventional EC effect.

In contrast, the inverse electrocaloric effect in PCa2ST (Figure 4d) appears near the AFE–to–PE phase transition at low electric fields (≤ 30 kV/cm). Below 295 K, a persistent but low-magnitude inverse EC response is observed. The maximum inverse adiabatic temperature change reaches −0.25 K, with the peak shifting toward lower temperatures as the electric field increases. This behavior is consistent with the PCa2ST phase diagram, which shows a negative dT/dE slope at the AFE–to–PE transition. Moreover, the magnitude of the inverse EC effect decreases with increasing electric field and transitions to a conventional response above the triple point at 30 kV cm^{-1} (see Supplementary Note 7). Above 295 K, the EC response becomes entirely conventional, as the material is stabilized in the paraelectric (PE) phase.

- We added the following text in the main text, in the section "Introduction".

Using an infrared camera, we measured the electrocaloric response under applied electric fields in Ca-doped PST compounds. Conventional EC effect was observed for Ca contents ≤ 2%, while inverse EC effect appeared for Ca contents ≥ 2%. For the ferroelectric compounds PST and 1%Ca-PST, maximum adiabatic temperature changes (ΔT_{adiab}) of 4.6 K at 313 K and 2.6 K at 285 K, respectively, were obtained at their FE–to–PE phase transitions. In 2%Ca-doped PST, which exhibits two successive phase transitions (FE–to–AFE and AFE–to–PE), both conventional and inverse EC effects were measured. At 115 kV cm^{-1}, maximum ΔT_{adiab} values of 2.0 K and 2.5 K were obtained at the FE–to–AFE and AFE–to–PE transitions, respectively. At low applied electric fields (< 30 kV cm^{-1}), a negligible inverse EC effect of −0.25 K was observed at the AFE–to–PE transition.
The antiferroelectric 4.6%Ca-PST compound exhibits a pronounced inverse EC effect at its AFE–to–PE transition, reaching a maximum ΔT_{adiab} of −0.6 K at 303 K. With increasing electric field and temperature, this inverse effect progressively transforms into a conventional EC effect, with a maximum ΔT_{adiab} of 1.8 K at 323 K.

2. The investigation of the inverse ECE in PCa4.6ST is focused on the high-temperature region near the AFE-PE transition. It would be highly informative to measure the EC response at lower temperatures (e.g., down to 230-260 K). This could reveal if a larger inverse ΔT_adiab occurs at lower temperatures, potentially near a putative AFE-FE transition induced by higher electric fields, providing a more complete picture of its caloric portfolio.

Answer: We thank the reviewer for this insightful question. The electrocaloric effect in PCa4.6ST was not investigated in the 230–260 K temperature range because our experimental setup (infrared camera and thermistor) does not allow EC measurements at such low temperatures.

However, complementary characterizations provide strong evidence regarding the phase transitions of this composition in 230-260K. Dielectric measurements and polarization–electric field (P–E) hysteresis loops were performed down to 173 K, and Raman spectroscopy under applied electric field was conducted down to 4 K. None of these measurements revealed any indication of a ferroelectric phase in PCa4.6ST.
Therefore, since no AFE–to–FE transition is observed, the electrocaloric response is expected to remain negligible in the 230–260 K range. The only phase transition identified in PCa4.6ST is the AFE–to–PE transition, where we measured an inverse electrocaloric effect.

3. The EC effect in PCa4.6ST was measured using a thermistor, with a maximum |ΔT_adiab| of ~0.7 K. Since this magnitude is within the detection capability of a modern IR camera, it is recommended to perform comparative measurements using the IR camera setup on PCa4.6ST. This would serve as a valuable cross-validation of the two direct measurement techniques used in the manuscript.

Answer: We thank the reviewer for the constructive comment. As suggested, we characterized PCa4.6ST using an infrared camera to measure the adiabatic temperature change during the application and removal of the electric field. The values obtained are in very good agreement with those measured using the thermistor. The corresponding data are presented in the figure below.

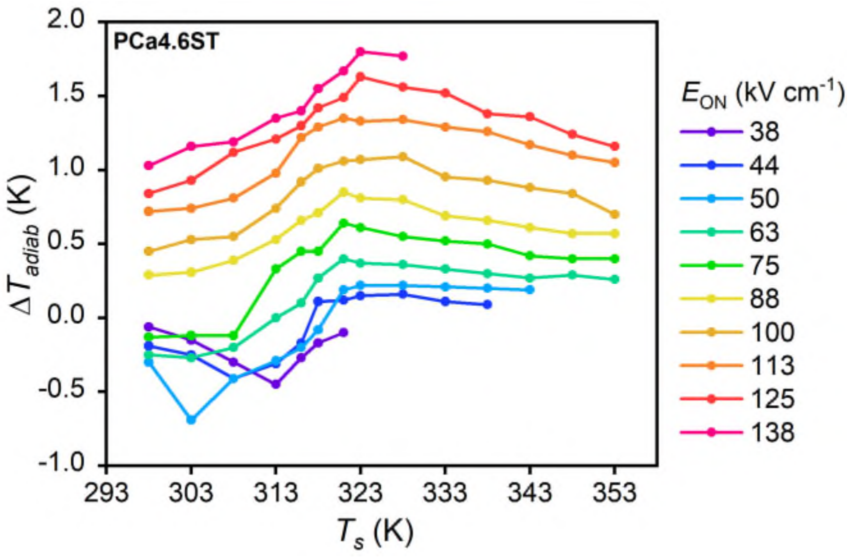

At the lowest applied electric field of 38 kV/cm, we observe an inverse EC effect of -0.5 K. With increasing electric field, the peak shifts toward lower temperatures, reaching a maximum value of -0.6 K at 303 K under 50 kV/cm. This behavior is consistent with the phase diagram of PCa4.6ST. At higher temperatures and electric fields, the inverse electrocaloric effect turns into a conventional one. A maximum conventional EC temperature change of 1.8 K is measured at 323 K under an electric field of 138 kV/cm. In contrast to

the thermistor characterization, here we were able to apply a higher electric field of 138kV/cm close to the material's breakdown field.

Modification:

- We added the direct characterization with IR camera to the manuscript. Figure 4 was updated. For clarity, the adiabatic temperature change for five different electric fields was added in the manuscript.

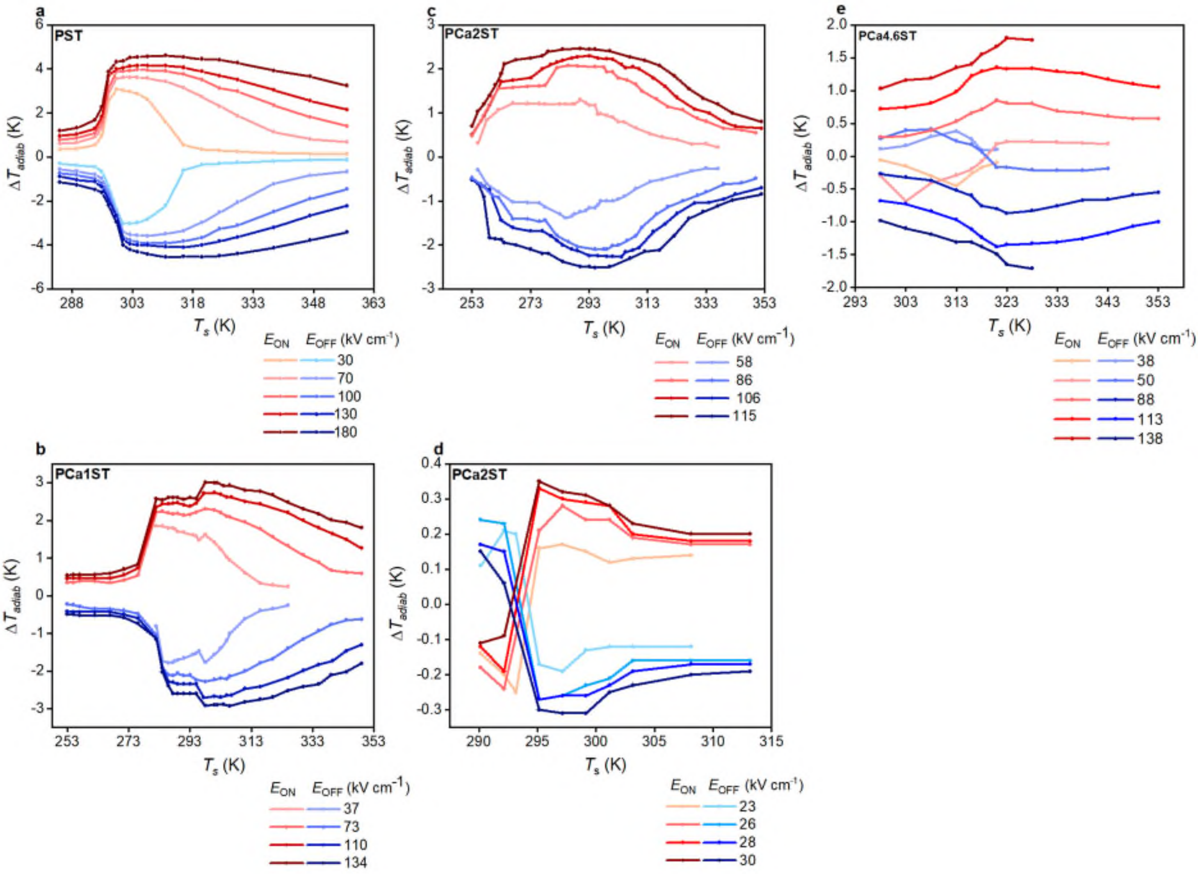

Figure: Electrocaloric effect of calcium-doped PST. Adiabatic temperature change, ΔT_{adiab}, of the different Ca-doped compounds measured at various starting temperatures (T_s) and electric fields (E). A conventional EC effect is observed in PST (a) and PCa1ST (b). In PCa2ST, a conventional EC effect is obtained at high electric fields (> 30 kV/cm) (c), whereas an inverse EC effect is observed near the AFE–PE transition at low electric fields (≤ 30 kV/cm) (d). PCa4.6ST exhibits an inverse electrocaloric effect (e).

- In Supplementary Information 12, we present a thorough analysis of the adiabatic temperature change of PCa4.6ST at all the electric fields studied.

- We added the following text in the main text, in the section "Electrocaloric effect of calcium-doped PST".

Finally, we investigate the electrocaloric (EC) effect in the antiferroelectric material PCa4.6ST. As shown in Figure 4e, an inverse electrocaloric effect is observed in the vicinity of the AFE–to–PE transition. At 313 K under an applied electric field of 38 kV/cm, an inverse EC temperature change of -0.5 K is measured. With increasing electric field, the EC peak shifts toward lower temperatures, reaching a maximum inverse temperature change of -0.6 K at 303 K under 50 kV/cm.

At higher temperatures and electric fields, the EC response transitions to a conventional effect. A maximum conventional EC temperature change of 1.8 K is recorded at 323 K under 138 kV/cm (Figure 4e). As indicated in Figure 3d, an inverse EC response is expected in this region because dT/dE is negative. Moreover, the P–E loops of PCa4.6ST (Figure 1d and Supplementary Figure~10) reveal an increase in polarization with temperature, corresponding to a positive ∂P/∂T and therefore a negative ΔTadiab, consistent with the Maxwell relation [5]. At sufficiently high electric fields and temperatures, the paraelectric phase becomes energetically favored, resulting in a positive (conventional) EC effect.

- We added the following text in the main text, in the section "Introduction".
  The antiferroelectric 4.6%Ca-PST compound exhibits a pronounced inverse EC effect at its AFE–to–PE transition, reaching a maximum ΔT_{adiab} of −0.6 K at 303 K. With increasing electric field and temperature, this inverse effect progressively transforms into a conventional EC effect, with a maximum ΔT_{adiab} of 1.8 K at 323 K.

4. The thermistor method employs a correction factor of 1.8 to account for thermal losses. The derivation and justification for this specific factor should be explicitly detailed in the Methods section to ensure reproducibility and transparency.

Answer: We thank the reviewer for their comment. We have performed measurements using the infrared camera (see answer to question 3) and we observe a good agreement with those obtained using the thermistor, thereby confirming the validity of the applied correction factor of 1.8.

In addition, we specified in the Methods section (Thermistor) the correction factor value of 1.8. The derivation and justification of this specific factor have been detailed in "Kutnjak, Z. & Rožič, B. Indirect and direct measurements of the electrocaloric effect. In *Electrocaloric Materials: New Generation of Coolers* (Springer, 2013), pp. 147–182 in Kutnjak, Z. & Rožič, B". This article is cited in the Methods Section.

5. The PFM data in Supplementary Note 9 provides good evidence for the AFE state. To further solidify these findings, it is recommended to: Present a more complete set of PFM switching spectroscopy data, including both on-field and off-field measurements for both PST and PCa4.6ST under identical DC voltage conditions. To further enhance the reliability of the microstructural analysis and minimize potential misinterpretation, the authors should address the issue of topography crosstalk (provide topography images in the supplementary data). This is a well-known artifact in PFM where the phase and amplitude signals can be strongly influenced by local surface topography, potentially mimicking or obscuring genuine electromechanical contrast.

Answer: We thank the reviewer for raising the point and giving us the opportunity to improve our manuscript.

As suggested by the reviewer, we have measured the local PFM hysteresis loops for PST and PCa4.6ST samples, including both on-field and off-field measurements under identical DC voltage conditions. The results are presented in the figures below. The off-electric field measurements confirm that PCa4.6ST is not ferroelectric. Moreover, the bias-on PFM amplitude loop of PCa4.6ST displays four maxima, which aligns well with the features observed in the polarization–electric field loops. This is further confirmed with a double hysteresis-like loop in the PFM phase response). The PFM amplitude loop of PST exhibits a butterfly-type hysteresis with two minima and a non-zero amplitude at zero bias, indicating a stable remanent ferroelectric polarization. This is confirmed by the PFM phase response, which shows a hysteresis loop. These features are consistent with ferroelectric behavior and confirm the polarization hysteresis loops previously measured in PST (see Figure 1a).

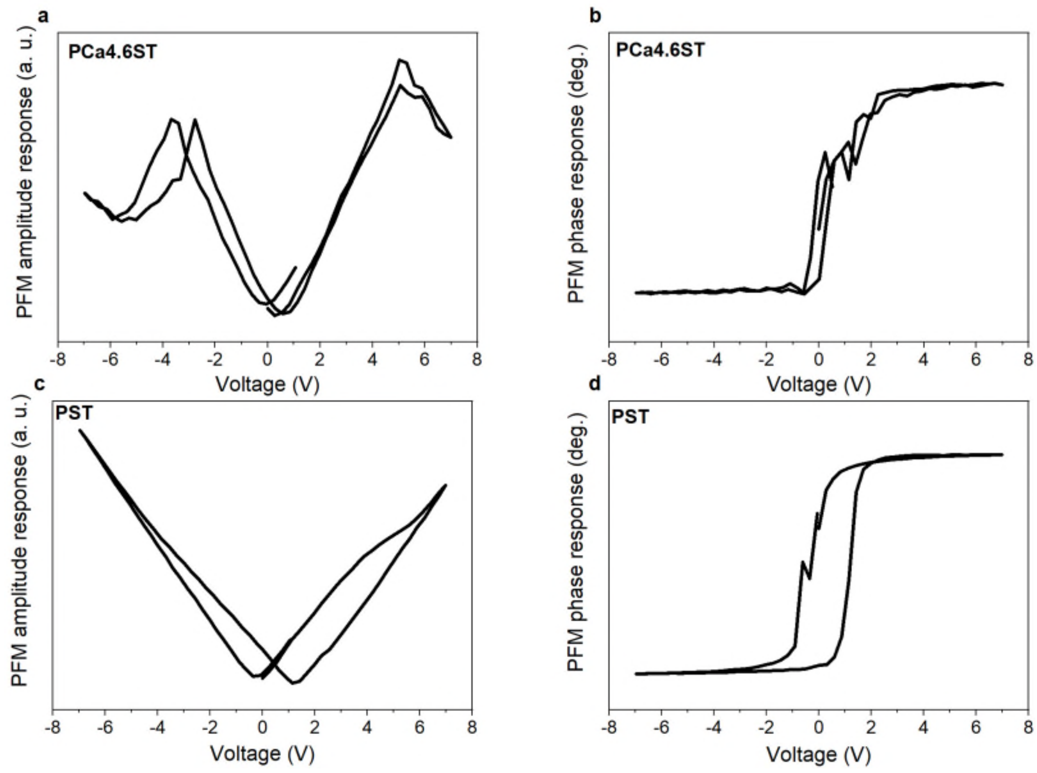

Figure: ON-electric field measurements. Local PFM amplitude hysteresis loops (a and c), and PFM phase hysteresis loops (b and d). Panels (a-b) correspond to PCa4.6ST, while panels (c-d) correspond to PST

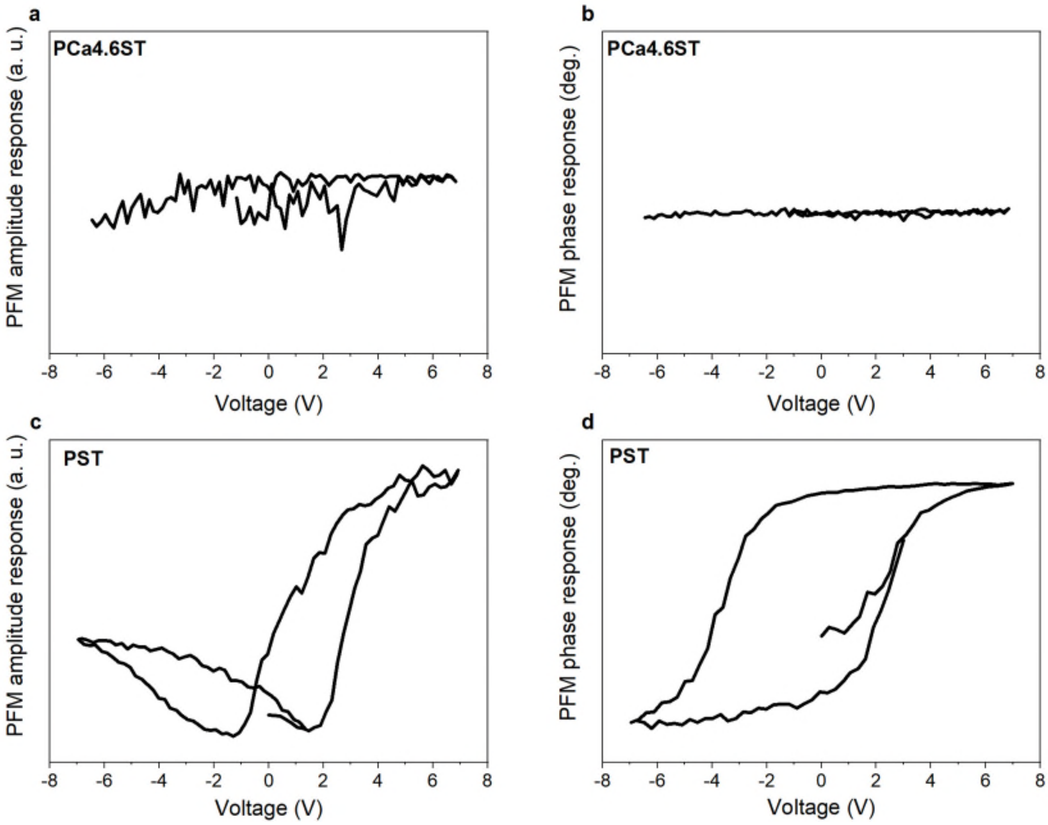

Figure: OFF-electric field measurements. Local PFM amplitude hysteresis loops (a and c), and PFM phase hysteresis loops (b and d). Panels (a-b) correspond to PCa4.6ST, while panels (c-d) correspond to PST.

To address the concern on topography crosstalk, AFM topography images of PCa4.6ST and PST samples were carried out along with the corresponding PFM phase images. From the comparison of topography and PFM phase images, we can conclude that the topography crosstalk in negligible. The ferroelectric domains features in PFM phase images (b, d) show no direct correlation with any topographic features. The results are described in the figure below:

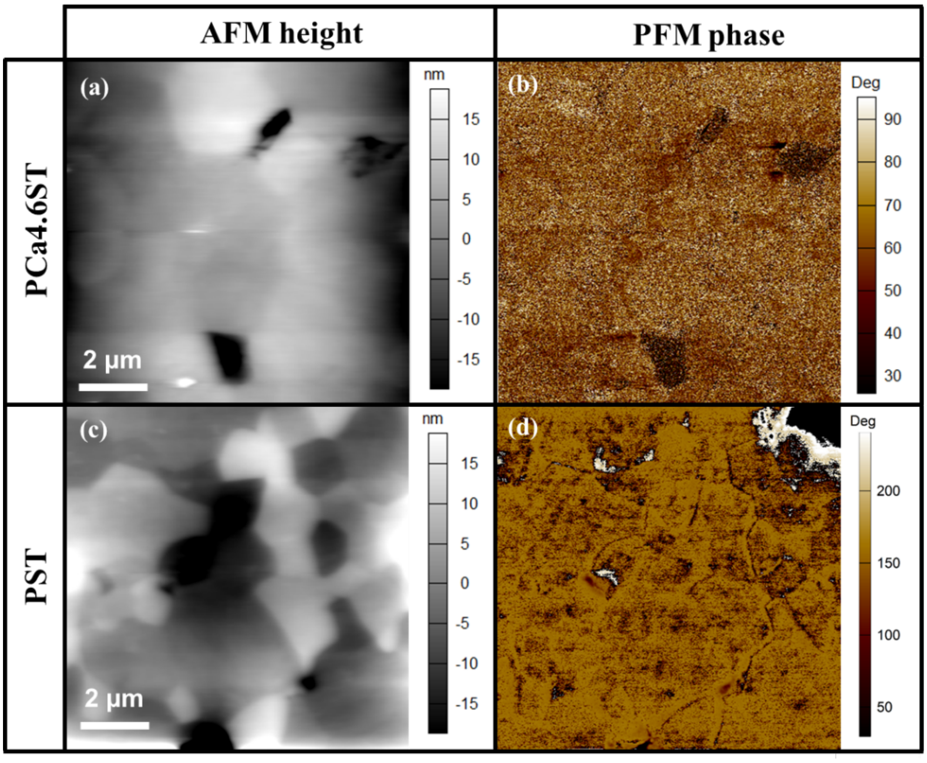

Figure: Piezoresponse force microscopy (PFM) of PST and PCa4.6ST. AFM topography height (a, c) and PFM phase images (b, d) of PST and PCa4.6ST. Panels (a, b) correspond to PCa4.6ST, while panels (c, d) correspond to PST.

Modification:

- We have added the three figures above to the Supplementary Note 9.
- We have revised the text as follows in Supplementary Note 9:

Supplementary Figure 23 shows the AFM topography images and PFM images of PST and PCa4.6ST. For PCa4.6ST, the PFM images under low AC excitation reveal only noise (Supplementary Figure 23b), indicating the absence of piezoelectric activity. In other words, the sample is non-polar. In contrast, the PFM image of undoped PST (Supplementary Figure 23d) shows distinct domain contrast, characteristic of a ferroelectric material. From the comparison of the topography and PFM phase images, we can conclude that the topography crosstalk is negligible. The ferroelectric domains features in PFM phase images (b, d) show no direct correlation with any topographic features.

Supplementary Figure 24 presents the PFM amplitude loops, and PFM phase loops for both PST and PCa4.6ST samples. The PFM amplitude loop of PST (Supplementary Figure 24c) exhibits a butterfly-type hysteresis with two minima and a non-zero amplitude at zero bias, indicating a stable remanent ferroelectric polarization. This is confirmed by the PFM phase response (Supplementary Figure 24d), which shows a hysteresis loop. These features are consistent with ferroelectric behavior and confirm the polarization hysteresis loops previously measured in

PST (see Figure 1a). On the other hand, the off-electric field measurements (Supplementary Figure 25) confirm that PCa4.6ST is not ferroelectric. Moreover, the bias-on PFM amplitude loop of PCa4.6ST (Supplementary Figure 24a) displays four maxima, which aligns well with the features observed in the polarization–electric field loops. This is further confirmed with a double hysteresis-like loop in the PFM phase response (Supplementary Figure 24b). These are clear signatures of antiferroelectric behaviour as demonstrated in [4] and [5].

- We have revised the PFM methods section:

  The local PFM amplitude and phase hysteresis loops were measured in the PFM switching spectroscopy (SS) mode with the pulsed DC step signal and the superimposed AC drive signal as described in [43]. The measurements were performed in the on-electric-field SS mode and off-electric-field SS mode for Ca-PST and PST samples, respectively. The waveform parameters were: the sequence of the rising DC step signal was driven at 20 Hz with a maximum amplitude of 8 V; the frequency of the triangular envelope was 0.2 Hz; a superimposed sinusoidal AC signal with an amplitude of 2 V and a frequency of ~ 320 kHz was used. One cycle was recorded in an on-electric field mode and off-electric-field mode.

6. The quality and clarity of several figures, particularly in the Supplementary Information (e.g., Supplementary Figure 13 on DSC measurements), should be improved. Please ensure all figures have consistently high resolution, clear axis labels, distinct data points, and legible legends to meet the journal's publication standards

Answer: We appreciate the reviewer's comment.

Modification:

We improved the quality of the 5 figures in the main text. Please find attached the high-resolution figures. Besides, the Supplementary Figures 1, 2, 8 and 13 have been improved for better clarity.

Reviewer #3 (Remarks to the Author):

This manuscript reports a comprehensive study on highly ordered A-site calcium-doped lead scandium tantalate (PbSc0.5Ta0.5O3, PST) ceramics, in which the phase transition behavior is modified to enable a tunable electrocaloric response across a broad temperature range. By introducing calcium dopants, the authors successfully stabilized an intermediate antiferroelectric (AFE) phase between the ferroelectric and paraelectric states for compositions with Ca ≥ 2%, allowing for the observation of both conventional electrocaloric effects ($\triangle T_{adiab}$ ≈ 4.6 K in pure PST) and inverse electrocaloric responses in heavily doped samples. The study systematically correlates macroscopic thermal and electrical measurements with microscopic piezoresponse force microscopy (PFM) and density functional theory (DFT) calculations to elucidate that the AFE stabilization arises from combined local lattice distortions and thermal effects rather than simple volume reduction. The work presents a generalizable strategy for expanding the operational window of PST-based regenerators to sub-ambient temperatures (down to 263 K), offering scientific significance and promising potential for cascaded electrocaloric cooling devices. However, there are several lacks in the manuscript that need to be solved or answered in its current state.

1. The explanation for the mechanism by which Ca doping stabilizes the AFE phase requires further elucidation. Currently, the attribution relies primarily on DFT energy comparisons and qualitative descriptions of local structural relaxations. Given the minute energy difference between the FE and AFE phases, zero-temperature calculations based on a single configuration are insufficient to fully support the experimental observations. It is recommended to provide statistical results from multiple Ca configurations, along with analyses of local Ca-O and Pb-O bond lengths or local volume variations, to more clearly demonstrate how Ca doping stabilizes the AFE phase in real materials.

Answer: We thank the referee for this thoughtful comment and for the suggestion to further clarify the microscopic mechanism by which Ca doping stabilizes the AFE phase. We fully agree that this is an important question. However, in our view, performing additional, highly computationally demanding DFT calculations for multiple Ca configurations in large supercells, would be unlikely to yield a definitive microscopic explanation for the stabilization of the AFE phase for the following reasons.

First, in both pure and Ca-doped PST, the energy differences between the FE (rII) and AFE phases obtained from our DFT calculations are only a few meV per A-site cation, i.e., on the order of the numerical accuracy of DFT for these systems. While sampling multiple Ca configurations in larger supercells would likely introduce some spread in the calculated FE–AFE energy differences, we expect this spread to remain within the same meV range. Consequently, even if certain configurations were found to favor the AFE phase,

such results would not allow us to unambiguously identify a ground-state configuration or to draw definitive conclusions regarding the stabilization mechanism of the AFE phase.

Second, our DFT results indicate that the FE rII phase remains lower in energy than the AFE phase up to at least 12.5% Ca substitution. In contrast, experimentally the transition to the AFE phase occurs already at Ca concentrations as low as ~2%. This strongly suggests that the stabilization of the AFE phase in the experiments is governed by effects not captured in our zero-temperature DFT calculations, such as finite-temperature effects or kinetic factors associated with synthesis. Such effects could of course be tackled by predictive simulations, but they would require the construction of suitable machine-learned interatomic potentials. This is a non-trivial endeavor for a disordered systems like Ca-doped PST – where energies need to be resolved within meV per A cation – and remains for future work.

2. The performance of PST is highly dependent on the degree of Sc/Ta ordering. While the authors achieved high ordering through extended annealing, further explanation is needed regarding whether Ca doping influences the kinetics of B-site cation ordering. Although XRD data indicate a high degree of order, a brief discussion would be beneficial: if the degree of ordering were lower, would the Ca-induced AFE phase still be stable?

Answer: We thank the reviewer for pointing this out. Indeed, it is well established in the literature [Stenger et al. Solid State Communications, Vol 32, 1979] that reducing the degree of Sc/Ta ordering in leads to relaxor behavior in PST. Please see figure below taken from a recent paper from Yin, R., Hou, Y., Lv, X. *et al. J. Adv. Ceram.* 14, 9221088 (2025). With a decreasing B-site cation order, PST loses its ferroelectric behaviour and becomes a relaxor, which in turn, we guess will not turn antiferroelectric with Ca doping.

[Figure Redacted]

Figure: Polarization versus electric field loops of PST bulk ceramic at different B-site cation order. The data were collected at -5°C under 40 kV/cm. This figure was taken from Yin, R., Hou, Y., Lv, X. et al. J. Adv. Ceram. 14, 9221088 (2025).

Modification:

We modified a part of the Discussion and added a brief discussion as suggested by the reviewer:

By doping highly ordered PST on the A-site with calcium, we modify its phase transitions and tune its electrocaloric response. Note that the calcium-doped PST compounds are

as highly ordered as undoped PST. We detect and measure antiferroelectricity in calcium-doped PST for concentrations greater than 1%. Specifically, PST and PCa1ST exhibit a transition from FE-to-PE, PCa2ST shows FE-to-AFE-to-PE, and PCa4.6ST displays an AFE-to-PE transition. Calcium stabilizes the AFE phase and destabilizes the FE. Having little Ca content (≤2%Ca) destabilizes the FE phase and at higher level calcium content (>2%Ca) the FE vanishes and the AFE is more stable. It is worth noting that the degree of B-site cation ordering plays a crucial role. In PST, a high level of disorder leads to relaxor behavior, which may hinder the stabilization of a well-defined antiferroelectric phase upon Ca doping.

3. For phase-change cooling materials, particularly those involving AFE-FE transitions associated with significant volume changes, cycling stability is a critical issue. Could the authors provide relevant experimental data or an explanation to demonstrate that the AFE-FE phase transition remains stable after repeated cycling?

Answer: We thank the reviewer for this comment. We performed electrocaloric measurements at the AFE-to-FE transition in PCa2ST upon multiple electric-field cycling (see Answer to question 1 or Supplementary Figure 15). This is 3 consecutive runs at four different electric fields. Moreover, cycling upon five electric fields in the isofield measurements carried out on PCa2ST (Figure 2e) shows the appearance and stability of the AFE-to-FE transition. We hope that this is convincing to confirm the stability of this transition.

4. The FE to AFE transition in PCa2ST is not discernible in the zero-field DSC curves but manifests as a distinct peak under iso-field conditions. This discrepancy requires further explanation. For instance, does this transition possess weak first-order or diffuse characteristics, or is it influenced by intragranular stress and energy barriers?

Answer: We thank the reviewer for the comment. Indeed, the FE to AFE peak in PCa2ST is not observed in the zero-field DSC curves. In fact, the FE phase is induced by an electric field, a minimum field of 15kV/cm. To measure the transition temperature of PCa2ST under no bias, we first field-cooled the sample at 16kV/cm and then we heated the sample at zero field to measure the transition. We observed two distinct thermal anomalies (see figure below): the first peak appears at 256 K, followed by a second peak at 297 K. The first peak clearly indicates a first-order transition from the FE phase to the AFE phase. Note that the transition temperature from FE to AFE is very close to the value we got from the electrical polarization hysteresis loops (refer to Figure 1c of the main text).

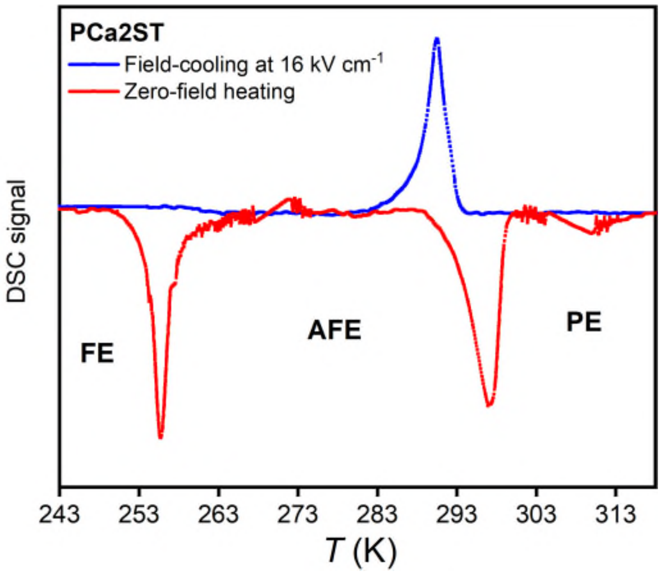

Figure: Field cooling and zero-field heating in PCa2ST. FE, AFE and PE refer to respectively, ferroelectric, antiferroelectric and paraelectric phases. Here, the sample was first field-cooled at 16 kV/cm and subsequently zero-field heated.

Modification:

We added the following text in the "Phase diagram of calcium-doped PST" section in the manuscript:

Note that, at zero field, the transition temperature of PCa2ST from the FE-to-AFE phase, which was not observed in the zero-field measurement (see Figure 2a), was determined by first field-cooling PCa2ST under an applied field of 16 kV cm^{-1}, followed by heating at zero field (see Supplementary Figure 13). The sharpness of the peak observed confirms the first-order character of the transition.

We added the following to the Supplementary Information.

As illustrated in Figure 2a, the differential scanning calorimetry (DSC) measurements taken without any bias don't show a transition from ferroelectric (FE) to antiferroelectric (AFE). This transition only becomes apparent during isofield measurements when an electric field of at least 15 kV/cm is applied. In this experiment, PCa2ST was initially cooled in the presence of an electric field of 16 kV/cm and then heated without bias to pinpoint its transition temperatures. We observe two distinct thermal anomalies (see Supplementary Figure 13): the first peak appears at 256 K, followed by a second peak at 297 K. The first peak clearly indicates a first-order transition from the FE phase to the AFE phase. Note that the transition temperature from FE to AFE is very close to the value we obtain from the electrical polarization hysteresis loops (refer to Figure 1c).

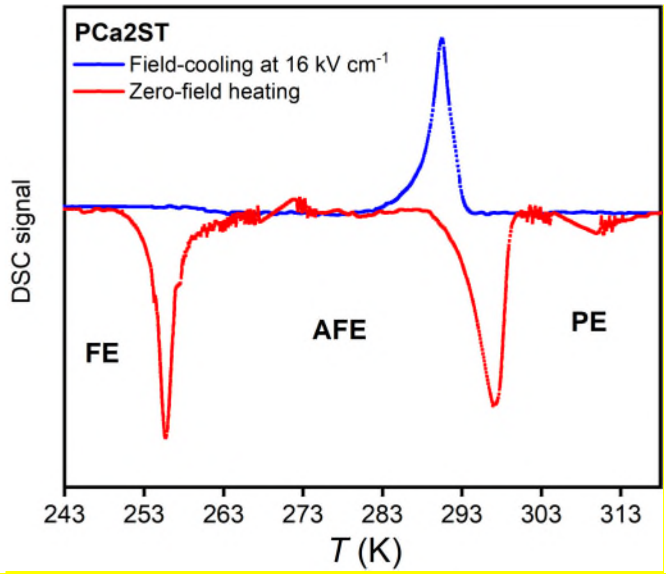

Supplementary Figure 13: Field cooling and zero-field heating in PCa2ST. FE, AFE and PE refer to respectively, ferroelectric, antiferroelectric and paraelectric phases. Here, the sample was first field-cooled at 16 kV cm−1 and subsequently zero-field heated.

5. The quantitative analysis of the electrocaloric effect should be strengthened. While the maximum ΔT_{adiab} values are presented, the lack of comparison regarding specific entropy change, thermal conductivity, sample dimensions (mass/thickness), and measurement techniques makes objective comparison within the system difficult.

Answer: We thank the reviewer for the constructive comment. The thermal conductivity of PST has been reported in several papers [Lheritier et al., Journal of the European Ceramic Society 41, 2021], reaching 1 $Wm^{-1}K^{-1}$.

Modification:

- We have added a table to compare the transition temperatures, latent heat and specific entropy changes (thermally driven) of the PCaxST samples investigated.

	PST		PCa1ST		PCa2ST				PCa4.6ST	
	FE to PE	PE To FE	FE to PE	PE To FE	FE to AFE	AFE to PE	PE to AFE	AFE to FE	AFE to PE	PE to AFE
T_0 (K)	297	294	282	276	256	297	294	-	317	317
Q_0 (J/kg)	932	620	544	422	109	275	182	-	261	277
ΔS_0 ($J/kg^{-1}K^{-1}$)	3.1	2.1	1.9	1.5	0.4	0.9	0.6	-	0.8	0.9

Supplementary Table 2: Thermal properties of Ca-doped PST. Transition temperatures T_0, latent heat Q_0, and entropy change ΔS_0 for PCaxST compositions at different transitions. FE, AFE and PE refer to respectively ferroelectric, antiferroelectric and paraelectric phases. The orange columns are on heating and the blue upon cooling. Note that the AFE to FE peak is a field induced transition (see Supplementary Note 7) expected below the temperature range of our equipment.

- We have added the following text to the manuscript:

Supplementary Table 2 summarizes the measured entropy change, ΔS_0, associated with thermally driven phase transitions in the different Ca-doped PST compounds. For PST and PCa1ST, thermally driving the ferroelectric (FE) to paraelectric (PE) transition yields

ΔS_0 values of 3.1 J K^{-1} kg^{-1} and 2.1 J K^{-1} kg^{-1}, respectively, indicating a decrease in entropy change with increasing Ca content.

In PCa2ST, two successive thermally driven transitions are observed. The FE to antiferroelectric (AFE) transition exhibits a ΔS_0 of 0.8 J K^{-1} kg^{-1}, while the AFE to PE transition yields a ΔS_0 of 0.9 J K^{-1} kg^{-1}. Finally, PCa4ST undergoes a single thermally driven AFE to PE transition, with an associated entropy change of 0.8 J K^{-1} kg^{-1}.

- We added the sample dimensions to the Supplementary information.

	PST	PCa1ST	PCa2ST	PCa4.6ST
Thickness (um)	86	82	125	80
Mass (mg)	6.96	11.7	33.02	25.06

Supplementary Table 3: Thickness and mass of PCaxST samples

- We added a table for a better comparison of the directly measured adiabatic temperature change of the PCaxST compounds. This table is now referenced in the manuscript.

PCaxST	T (K)	ΔT_{adiab} (K)	E_{on} (kV cm^{-1})
PST	297	4.6	180
PCa1ST	282	3.0	134
PCa2ST	256	2.0	115
PCa2ST	292	−0.25	23
PCa2ST	297	2.5	115
PCa4.6ST	303	−0.6	50
PCa4.6ST	323	1.8	138

Supplementary Table 4: Adiabatic temperature change of PCaxST compounds. ΔT_{adiab} measured at different temperatures and applied electric fields for Ca-doped PST compounds. The adiabatic temperature change was collected with an IR camera.

Furthermore, since a thermistor was used for PCa4.6ST while an IR camera was used for other samples, it is recommended to include a discussion on the consistency between these two thermometry methods, or provide a calibration comparison in the Supplementary Information.

Answer: We thank the reviewer for the constructive comment. As suggested, we characterized PCa4.6ST using an infrared camera to measure the adiabatic temperature change during the application and removal of the electric field. The values obtained are in very good agreement with those measured using the thermistor. The corresponding data are presented in the figure below.

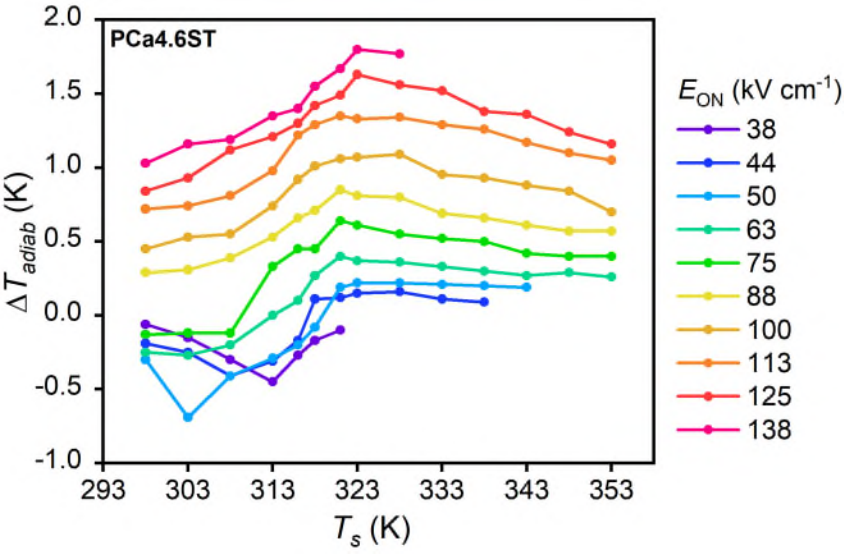

At the lowest applied electric field of 38 kV/cm, we observe an inverse EC effect of -0.5 K. With increasing electric field, the peak shifts toward lower temperatures, reaching a maximum value of -0.6 K at 303 K under 50 kV/cm. This behavior is consistent with the phase diagram of PCa4.6ST. At higher temperatures and electric fields, the inverse electrocaloric effect turns into a conventional one. A maximum conventional EC temperature change of 1.8 K is measured at 323 K under an electric field of 138 kV/cm. In contrast to the thermistor characterization, here we were able to apply a higher electric field of 138kV/cm close to the material's breakdown field.

Modification:

- We added the direct characterization with IR camera to the manuscript. Figure 4 was updated. For clarity, the adiabatic temperature change for five different electric fields was added in the manuscript.

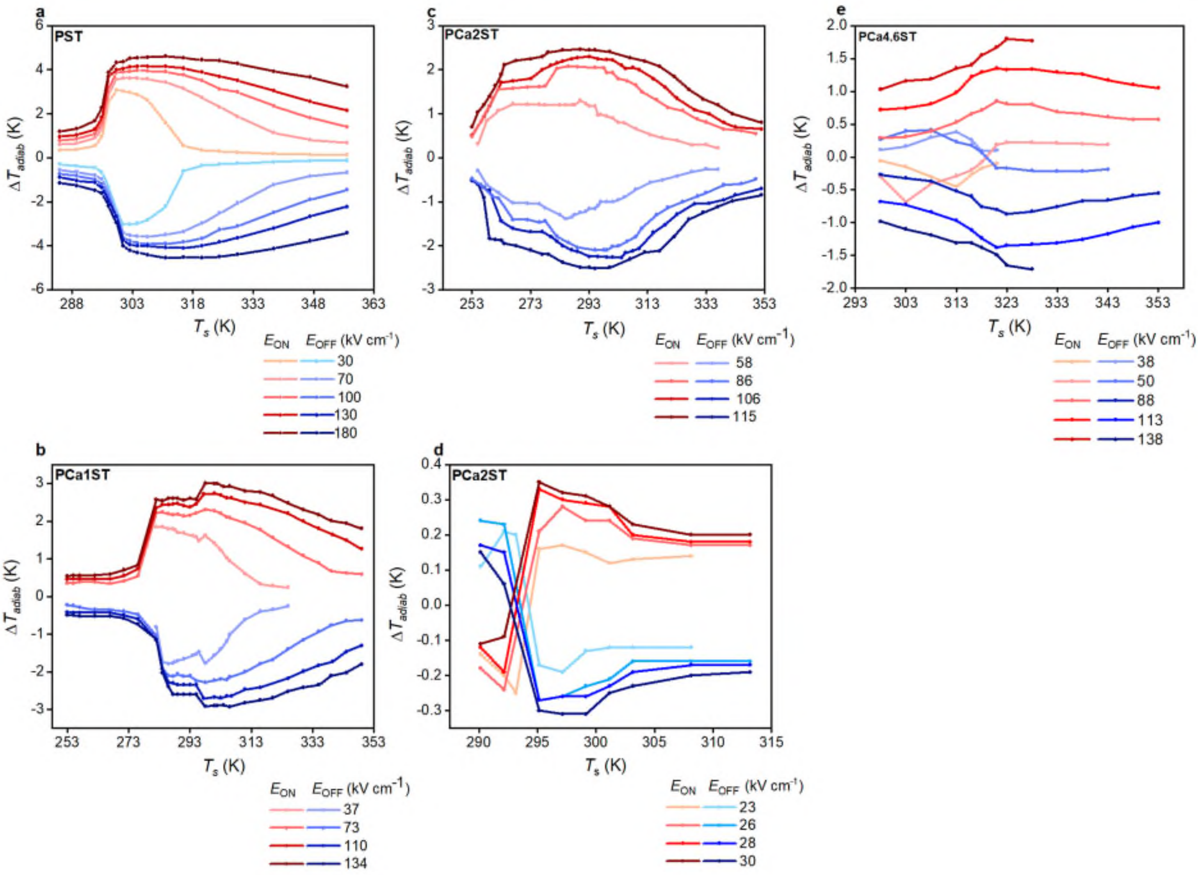

Figure: Electrocaloric effect of calcium-doped PST. Adiabatic temperature change, ΔT_{adiab}, of the different Ca-doped compounds measured at various starting temperatures (T_s) and electric fields (E). A conventional EC effect is observed in PST (a) and PCa1ST (b). In PCa2ST, a conventional EC effect is obtained at high electric fields (> 30 kV/cm) (c), whereas an inverse EC effect is observed near the AFE–PE transition at low electric fields (≤ 30 kV/cm) (d). PCa4.6ST exhibits an inverse electrocaloric effect (e).

- In Supplementary Information 12, we present a thorough analysis of the adiabatic temperature change of PCa4.6ST at all the electric fields studied.

- We added the following text in the main text, in the section "Electrocaloric effect of calcium-doped PST".

Finally, we investigate the electrocaloric (EC) effect in the antiferroelectric material PCa4.6ST. As shown in Figure 4e, an inverse electrocaloric effect is observed in the vicinity of the AFE–to–PE transition. At 313 K under an applied electric field of 38 kV/cm, an inverse EC temperature change of -0.5 K is measured. With increasing electric field, the EC peak shifts toward lower temperatures, reaching a maximum inverse temperature change of -0.6 K at 303 K under 50 kV/cm.

At higher temperatures and electric fields, the EC response transitions to a conventional effect. A maximum conventional EC temperature change of 1.8 K is recorded at 323 K under 138 kV/cm (Figure 4e). As indicated in Figure 3d, an inverse EC response is expected in this region because dT/dE is negative. Moreover, the P–E loops of PCa4.6ST (Figure 1d and Supplementary Figure~10) reveal an increase in polarization with temperature, corresponding to a positive ∂P/∂T and therefore a negative ΔTadiab, consistent with the Maxwell relation [5]. At sufficiently high electric fields

and temperatures, the paraelectric phase becomes energetically favored, resulting in a positive (conventional) EC effect.

- We added the following text in the main text, in the section "Introduction".
  The antiferroelectric 4.6%Ca-PST compound exhibits a pronounced inverse EC effect at its AFE–to–PE transition, reaching a maximum ΔT_{adiab} of −0.6 K at 303 K. With increasing electric field and temperature, this inverse effect progressively transforms into a conventional EC effect, with a maximum ΔT_{adiab} of 1.8 K at 323 K.

6. The manuscript reports an inverse electrocaloric effect in the 4.6% Ca-doped sample with a ΔT_{adiab} ≈ -0.7 K. While this represents an interesting physical phenomenon, the magnitude is relatively small. Given that the authors propose the potential of these materials for cascaded devices in the Conclusion, it is recommended to provide a more objective evaluation in the Discussion section regarding the actual efficiency contribution of this "inverse electrocaloric effect" in a practical refrigeration cycle. Alternatively, the authors should explicitly clarify whether the primary significance of this specific composition lies in elucidating the scientific mechanism rather than in direct high-efficiency cooling performance.

Answer: Thank you for raising this point. Indeed, we agree with the reviewer on the fact that magnitude of ΔT_{adiab} in the 4.6%Ca-doped is relatively small and the negative electrocaloric effect measured is not practical for implementation in a cascading device. As detailed in the Discussion, we do not propose using 4.6%Ca-PST in a cascading device as its negative electrocaloric effect would counteract the positive electrocaloric effect of the adjacent material. Instead, PST and 2%Ca-doped PST are more promising candidates for a potential cascading device.

Modification:
To clarify this point, we added the following text in the Discussion section:

Figure 5 illustrates how the conventional electrocaloric (EC) effect of calcium-doped PST (undoped PST, PCa1ST, and PCa2ST) can be exploited to extend the operating temperature range of PST toward lower temperatures. A maximum adiabatic temperature change (ΔT_{adiab}) of 1.8 K in PST, observed between 293 K and 313 K, has been implemented in a state-of-the-art electrocaloric cooling device [4]. Based on this benchmark, we adopt ΔT_{adiab} ≥ 1.8 K as a threshold criterion to define the practical operating temperature range. Undoped PST operates between 295 K and 353 K with ΔT_{adiab} ≥ 1.8 K. Upon Ca doping, the operating window shifts toward lower temperatures: PCa1ST (1% Ca) operates between 280 K and 336 K, while PCa2ST (2% Ca) covers the range from 263 K to 323 K. In an electrocaloric regenerator, combining PCa2ST with PST would enable coverage of a broad temperature range from 263 K to 353 K while maintaining ΔT_{adiab} ≥ 1.8 K.
For comparison, the electrocaloric effect reported for less ordered bulk PST [32] reaches approximately 1.8 K over a temperature interval of 288–318 K. The 4.6% Ca-doped

composition is not considered here, as its small negative temperature change under applied electric field would counteract the positive EC response of the other compositions.

Responses to the referees:

Reviewer#1 (Remarks to the Author):

The authors have satisfactorily addressed all my concerns. I therefore recommend acceptance in its present form.

Answer: We appreciate the reviewer for the positive comments on our manuscript.

Reviewer #2 (Remarks to the Author):

The authors have addressed all reviewer comments well. The new experiments—low-field ECE measurements on PCa2ST, PFM switching spectroscopy with topography analysis, and IR camera measurements on PCa4.6ST—have improved the manuscript. All concerns have been resolved. The manuscript is now suitable for publication in Nature Communications.

Answer: We appreciate the reviewer for the positive comments on our manuscript.

Reviewer #3 (Remarks to the Author):

The authors have satisfactorily addressed all comments.

Answer: We appreciate the reviewer for the positive comments on our manuscript.

This manuscript presents an interesting approach to broadening the electrocaloric operating window of PST through Ca doping. The authors show that Ca doping stabilizes an antiferroelectric phase and enables a tunable electrocaloric effect spanning roughly 263–353 K, with peak ΔT_{adiab} values of ~2 K under high electric fields. The combined use of DSC, dielectric spectroscopy, Raman scattering, PFM, and DFT calculations provides a rich dataset. Nevertheless, several points require further clarification or expansion before the manuscript can be considered for publication.

Major comments

1. Given the diffuse dielectric and P–E responses observed in the Ca-doped samples, it would be helpful for the authors to discuss whether increasing Ca content could lead to local coexistence of AFE and FE regions near the phase boundaries. For PCa4.6ST, the dielectric anomaly observed around 15 °C (Supplementary Fig. 11d) is not clearly explained in the manuscript. It would be important for the authors to clarify whether this feature is associated with an intrinsic phase transition, with a possible incommensurate–commensurate transition commonly reported in antiferroelectrics?

2. The Tc shows a non-monotonic trend with Ca doping, reaching a minimum at 1% Ca. What is the mechanism that causes Tc to decrease initially and then increase again with higher Ca content?

3. The explanation invoking Ca-rich and Ca-poor regions at low doping levels is speculative, as no experimental evidence or literature reference is provided to support this claim. The authors should either substantiate this statement with appropriate data (e.g., compositional mapping) or clearly indicate it as a hypothesis.

4. The cooling curves in Fig. 2 exhibit unusually large noise and distorted features, which cast doubt on the physical meaning of these data.

5. It should be noted that the apparent broadening of the operating temperature range depends strongly on the chosen ΔT_{adiab} threshold. If one requires $\Delta T_{adiab} > 2$ K, Ca doping does not significantly extend the usable span; the broadening becomes evident only when a lower threshold (e.g., >1 K) is considered. To more rigorously support the claim of an expanded operating window, a systematic comparison of ΔT_{adiab} with key literature PST compositions would be helpful. In addition, the device-oriented statements should be moderated unless supported by realistic modelling of multilayer architectures or cycling performance.

6. The manuscript states that the inverse EC effect at low fields is too small to be detected. Could the authors clarify whether highly precise thermistor measurements were attempted to resolve this effect? In addition, for a fair comparison of measurement reliability, the infrared-camera and thermistor methods should ideally be applied to the same sample under identical experimental conditions.

7. Supplementary Note 11 provides extensive DFT data (energies, phonons, pressure dependence, distortion-mode amplitudes). However, the main text compresses these results too aggressively. For clarity, the authors should explicitly state the rII–AFE energy

differences (Supplementary Fig. 21a), summarize how these energies evolve with Ca doping (Supplementary Tables 3–6), and clearly indicate that the stabilization of the AFE phase cannot be explained by volume changes alone, consistent with the DFT results.

8. Several figures in the main text appear to be drawn too roughly, which affects their readability. In particular, the axis labels in Figures 1–4 are too small and should be enlarged to improve clarity.

9. The claim that the FE–AFE–PE phase sequence differs from $PbZrO_3$-based systems is not accurate. This sequence is actually common in $PbZrO_3$-derived antiferroelectrics (see early work by Wuyi Pan and colleagues). The authors should revise this statement and cite appropriate literature.

Minor comments

1. In Supplementary Figure 1, the scale bars are difficult to read. Please clearly indicate the scale bar values (e.g., add the length in the caption or enlarge the labels).
2. In Supplementary Fig. 13, two red points appear on the right side of the zero-field heating curve, but their meaning is not explained.
3. "PST" should be defined upon first use in the abstract.
4. In Supplementary Fig. 12c, the loss tangent during heating shows two abrupt jumps at low temperatures that are not seen in the cooling curve. These features look like measurement artifacts and the authors should clarify their origin.
5. In Supplementary Fig. 19c, two red arrows are shown on the PFM phase loop, but their meaning is not explained in either the figure caption or the text.
6. The references are not formatted according to the journal's guidelines, and several citations (e.g., Ref. 16) are used inappropriately. Please revise the reference list and ensure accurate citation throughout the manuscript.
